# Expression and prognostic value of long non-coding RNA H19 in glioma via integrated bioinformatics analyses

**DOI:** 10.18632/aging.102819

**Published:** 2020-02-20

**Authors:** Yilei Xiao, Zipeng Zhu, Jianxiong Li, Jie Yao, Haitao Jiang, Ran Ran, Xueyuan Li, Zhiqiang Li

**Affiliations:** 1 Department of Neurosurgery, Liaocheng People’s Hospital, Liaocheng 250000, Shandong Province, P.R. China; 2 Department of Chemotherapy, Chinese PLA General Hospital, Beijing 100036, P.R. China; 3 Department of Biological Repositories, Zhongnan Hospital of Wuhan University, Wuhan 430071, Hubei Province, P.R. China; 4 Department of Neurosurgery, Zhongnan Hospital of Wuhan University, Wuhan 430071, Hubei Province, P.R. China

**Keywords:** H19, bioinformatics, glioma, immune infiltration

## Abstract

Numerous discoveries have elucidated that long noncoding RNAs (lncRNAs) play a critical role in cancer malignant progression. However, their potential involvement in gliomas remains to be explored. Herein, the expression level of lncRNA H19 in glioma tissues, and its relevance with clinical characteristics were analyzed through Oncomine. The results showed that H19 was highly expressed in glioma tissues and its expression increased with the increase of malignancy. Next, GTEx and TCGA data were downloaded for differently expressed genes (DEGs) identification, Gene Ontology (GO) and Kyoto Encyclopedia of Genes and Genomes (KEGG) analyses, and the correlation analyses between H19 expression and clinic features. Radiation therapy had a good effect on glioblastoma multiforme (GBM), but didn’t have a good effect on low grade glioma (LGG). Meanwhile, the expression level of H19 could act as an indicator molecule indicating the effect of radiotherapy. Finally, gene set enrichment analysis (GSEA) and immune infiltration analysis were conducted. It was found that H19 could affect the immune infiltration level of glioma through copy number variations, thus affecting the prognosis of glioma patients. Collectively, H19 may be involved in the occurrence and development of glioma, and has potential reference value for the relief and immunotherapy of glioma.

## INTRODUCTION

Glioma is the most common primary tumor of the brain and spinal cord [1]. Despite advances in surgical techniques and clinical protocols, treatment of high-grade gliomas is still a challenge, with low treatment success rate and overall survival rate. Challenges comprise of the molecular complexity of gliomas and the inconsistencies in histopathological grading, which lead to inaccurate predictions of disease progression and failure of standard treatment [2]. Currently, the standard treatment for glioma patients is postoperative radiotherapy and adjuvant chemotherapy [3]. Because of therapeutic resistance and tumor recurrence, efforts are under way to identify the basic molecules that regulate tumor progression and to provide new approaches for individualized treatment of glioma patients [4].

Long non-coding RNAs (lncRNA) are non-coding RNAs with a length of more than 200 nt, which play an important role in transcriptional silencing, transcriptional activation, chromosome modification and nuclear transport [5]. Mounting evidence revealed that lncRNAs could serve as key molecules in the development and metastasis of tumors [6–9], including gliomas. Some researchers have demonstrated that lncRNAs participated in the progression of gliomas. For instance, a paper from Qi Y et al., reported that lncRNA HOXD-AS2 promoted the progression of gliomas and might act as a promising target for the diagnosis and therapy of gliomas [10]. In addition, lncRNA MT1JP inhibits the proliferation, invasion and migration of glioma cells by activating the PTEN/Akt signaling pathway [11]. However, additional important lncRNAs correlated with gliomas need to be further explored.

**Figure 1:**
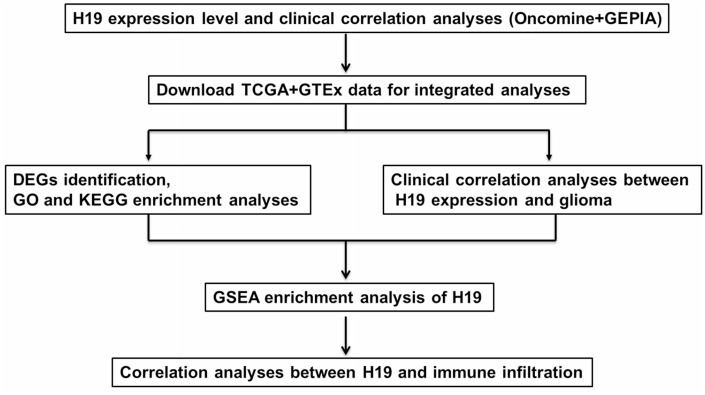
**The pipeline designed for this work.** Abbreviations: TCGA: The Cancer Genome Atlas; DEGs: differentially expressed genes; GO: gene ontology; KEGG: Kyoto Encyclopedia of Genes and Genomes; GSEA: gene set enrichment analysis.

lncRNA H19 was proposed as a regulator in 1990 and was the first lncRNA to be discovered [12]. H19 has been reported to participate in the regulation of a variety of diseases [13], especially in gliomas. Relevant reports exhibited that H19 promoted proliferation and invasion in human glioma cells [14]. H19 interacted with miR-140 to regulate glioma growth [15]. LncRNA H19 promotes tumor growth through its derivative miR-675 in gliomas [16]. Herein, the relationship between the expression level of H19 and glioma was investigated from the perspective of bioinformatics. First, we analyzed the expression of H19 in glioma tissue, the relationship between H19 and glioma patient's clinical characteristics, and the prognostics value of H19 by Oncomine and GEPIA database. Then, TCGA and GTEx data were used to comprehensively analyze the clinical correlations, differentially expressed gene (DEGs), Gene Ontology (GO) and Kyoto Encyclopedia of Genes and Genomes (KEGG) pathways. Finally, gene set enrichment analysis (GSEA) and immune infiltration analysis were performed on H19. The workflow of this study was shown in Figure 1.

## RESULTS

### High expression of H19 in glioma tissues

**Figure 2:**
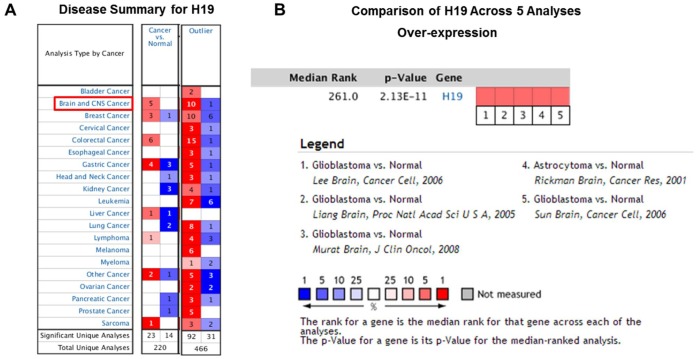
**High expression of H19 in glioma tissues based on Oncomine.** (**A**) In Brain and CNS cancer, H19 was significantly upregulated in five studies. Red represents high expression, blue represents low expression. The darker the color, the greater the significance. (**B**) Comparison of H19 expression across five analyses, and red means high expression. *P*<0.01.

Based on Oncomine, a total of 220 different studies on H19 were included in Cancer vs Normal analysis. Among them, there were five studies on the up-regulation of H19 and no studies on its downregulation in Brain and CNS (central nervous system) cancers, which suggested that H19 was up-regulated in brain and CNS cancers. As for the outlier analysis of H19, 466 different studies on H19 have been included in Oncomine database. Among them, there were 10 studies about the up-regulation of H19 and one study about its downregulation in Brain and CNS cancers (Figure 2A, *P* <0.01*,* Gene Rank<Top 10%).

**Figure 3:**
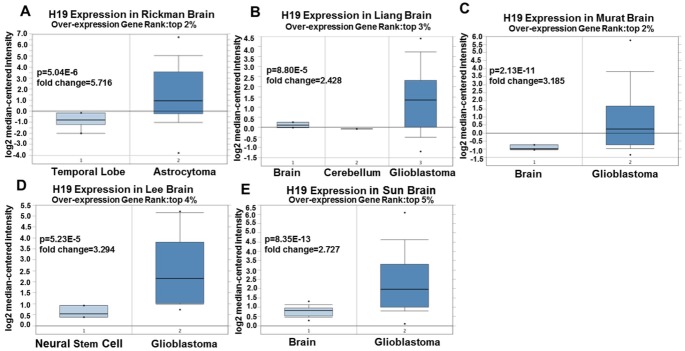
**Expression level of H19 in each study based on Oncomine.** (**A**) Rickman Brain, (**B**) Liang Brain, (**C**) Murat Brain, (**D**) Lee Brain, (**E**) Sun Brain. Top 2%/ (3%)/ (4%) / (5%) refers to the top 2% / (3%)/ (4%)/ (5%) of genes; *P* <0.01.

**Figure 4:**
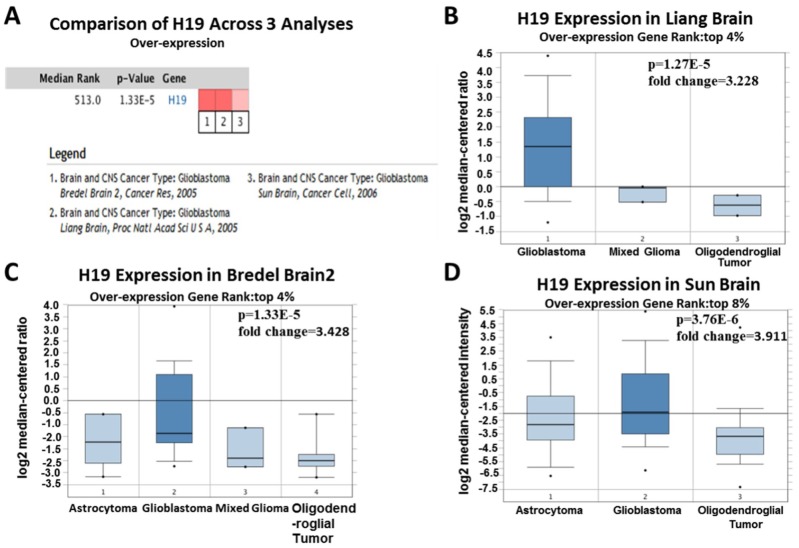
**Analysis of relationship between the H19 expression and the malignant degree of glioma.** (**A**) Comparison of H19 expression across three analyses, and red means high expression. H19 expression in (**B**) Liang Brain, (**C**) Bredel Brain 2, and (**D**) Sun Brain. The expression level is evaluated via the median line; Top 4% / (8%) refers to the top 4%/ (8%) of genes; *P*<0.01.

Since 2001, we have found eight studies involving the differential expression of H19 between glioma and normal tissues in the Oncomine database, with a total of 567 samples. Among them, there were five studies that met the screening criteria, and comprehensive analysis of the five studies showed that Median Rank=261, *P*-value =2.13E-11, indicating that H19 was highly expressed in glioma tissues and was mainly concentrated in GBM (Figure 2B, *P* <0.01). Furthermore, it was found that the expression level of H19 in glioma tissues was clearly higher than that in the normal control group in each study (Figure 3A–3E, *P* <0.01). These findings illustrated that H19 was elevated in gliomas, especially in GBM, indicating that H19 might play an important regulatory role in glioma progression. To further determine whether H19 is related to the malignant degree of gliomas, Cancer vs Cancer analysis was conducted (Figure 4A). The results showed that H19 in GBM was observably higher than that in other types of gliomas, and the expression level of H19 increased with the malignant degree (Figure 4B–4D, *P* <0.01). These results suggested that H19 might be a potential molecular marker for predicting the malignancy of gliomas.

### Clinical correlation analyses based on Oncomine and GEPIA

**Figure 5:**
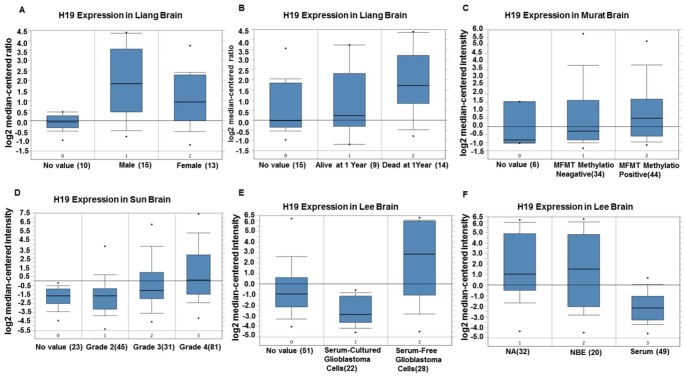
**The correlation between H19 expression level and clinical characteristics of glioma patients by Oncomine.** (**A**) Comparison of the expression level of H19 in men and women. (**B**) Comparison of the expression level of H19 in patients alive or dead after one year. (**C**) The expression level of H19 in patients with MGMT methylation negative was lower than that in patients with MGMT methylation positive. (**D**) The expression of H19 elevated with the increase of staging. (**E**, **F**) H19 expression in serum-free cultured glioma cells was higher than that in serum cultured glioma cells. Abbreviations: NA, No value; NBE, Serum-free culture.

Based on the Oncomine database, we performed a range of correlation analyses between the expression level of H19 and clinical features in glioma patients. The results demonstrated that the expression level of H19 in men was up-regulated compared to that in women (Figure 5A). One year later, the expression level of H19 in living patients was lower than that in the deceased (Figure 5B). The expression level of H19 in patients with negative MGMT methylation was lower than that in patients with positive MGMT methylation (Figure 5C); and H19 expression increased with the increase of stage (Figure 5D). Interestingly, the expression level of H19 in serum-free cultured glioma cells was higher than that in serum cultured ones (Figure 5E, 5F). These results suggested that H19 might be associated with glioma progression.

### Relationship between H19 expression and prognosis of glioma patients

**Figure 6:**
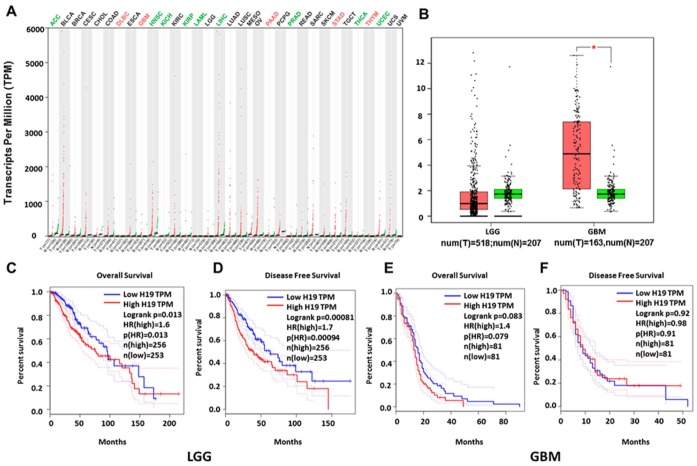
**Relationship between H19 expression and prognosis of glioma patients based on GEPIA database.** (**A**) H19 was significantly upregulated in various tumors, such as GBM, PAAD and STAD. (**B**) Expression level of H19 in LGG and GBM in comparison with the normal control. *, *P* < 0.05. (**C**–**F**) The relationship between H19 expression levels and overall and disease-free survival in LGG and GBM as analyzed by GEPIA database. Abbreviation: LGG, low grade glioma; GBM, glioblastoma; num, number; T, tumor; N, normal.

In order to further determine the correlation between H19 expression and gliomas and verify the results from Oncomine analyses, we analyzed GEPIA data and found that H19 was significantly up-regulated in DLBC (Lymphoid Neoplasm Diffuse Large B-cell Lymphoma), GBM (glioblastoma multiforme), PAAD (pancreatic cancer), STAD (gastric cancer), and THYM (Thymoma) (Figure 6A). And it was markedly downregulated in ACC (adrenocortical carcinoma), KIRP (renal papillary cell carcinoma) and LIHC (liver cancer), etc. Interestingly, the expression of H19 showed no significant difference in LGG compared with that in normal control, but was observably upregulated in GBM (Figure 6B). Prognostic analysis revealed that high expression of H19 would lead to a short overall survival and disease-free survival in patients with LGG (Figure 6C, 6D *P* < 0.05), and the expression level of H19 was not associated with the prognosis of patients with GBM (Figure 6E, 6F, *P* > 0.05). These results indicated that H19 was conspicuously upregulated in GBM and had prognostic value in LGG, indicating that H19 had important regulatory functions in gliomas.

### Low expression of H19 in human brain tissue

**Figure 7:**
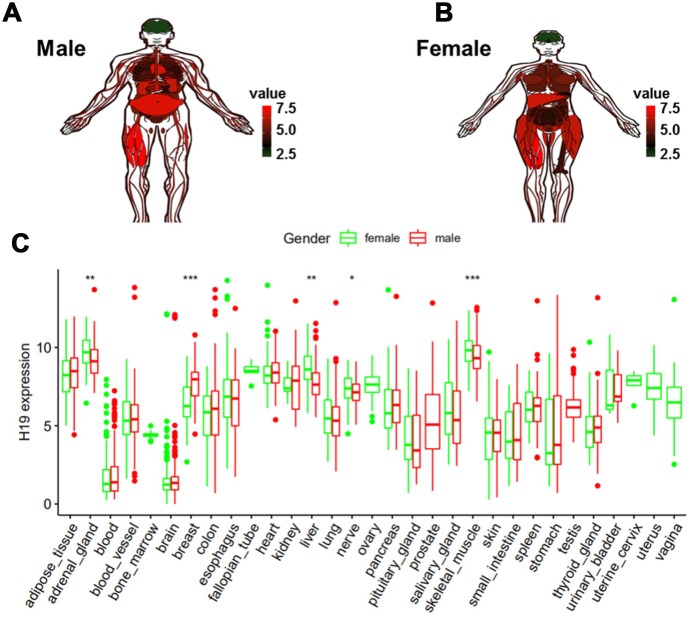
**Expression level of H19 in normal human tissues as analyzed by GTEx data.** (**A**) Compared to other normal tissues, the expression level of H19 in brain tissue in (**A**) men and (**B**) women. (**C**) Expression level of H19 in tissues of different genders.

In order to determine the expression level of H19 in normal human tissues, GTEx data analysis was conducted. Red represented high expression, black represented median expression, and green represented low expression. As shown in Figure 7A and 7B, H19 was highly expressed in most tissues, while underexpressed in the head, and there was no significant difference in brain tissues of different genders (Figure 7C). These results suggested that we could integrate normal brain tissues from men and women, as well as glioma tissues in the subsequent analyses.

### DEGs identification and GO and KEGG pathway analyses

**Figure 8:**
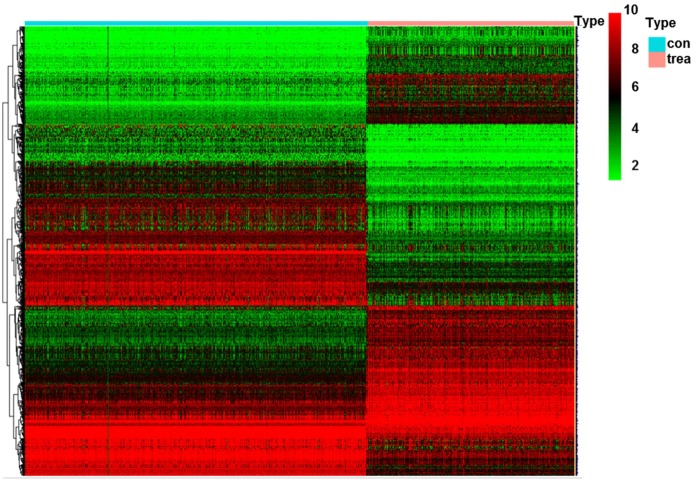
**Heatmap of DEGs in glioma.** Abbreviation: DEGs, differentially expressed genes; con, control; trea, treatment.

**Figure 9:**
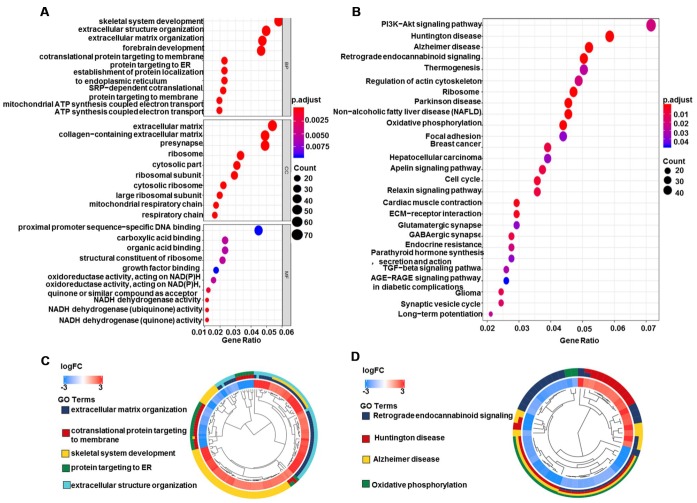
**GO and KEGG pathway analyses of DEGs in glioma.** (**A**) Biological process, cell components, and molecular function enrichment analyses of DEGs. (**B**) KEGG pathway analysis of DEGs. Only pathways with a P-value <0.05 are presented. (**C**) GO and (**D**) KEGG pathway cluster analyses. Abbreviation: DEGs, differentially expressed genes; BP, Biological process; CC, cell components; MF, Molecular function; FC, fold change.

A total of 1,427 DEGs (|logFC|>2, FDR<0.05) were identified in glioma data by differential expression gene analysis (Supplementary Table 1, Figure 8). GO analysis demonstrated that the biological processes of these DEGs were significantly enriched in the development of skeletal system, extracellular structure and forebrain. Cell components were mainly concentrated in extracellular matrix, collagen containing extracellular matrix and ribosomes. Molecular functions were obviously enriched in growth factor composition, ribosome structure composition and organic acid binding (Figure 9A). KEGG pathway analysis revealed that DEGs were mainly concentrated in PI3K−Akt signaling pathway, TGF−beta signaling pathway and tumor-related signaling pathway (Figure 9B). Meanwhile, we conducted cluster analyses of the top GO and pathways (Figure 9C, 9D). The top GO terms were enriched in extracellular matrix organization, cotranslational protein targeting to membrane, skeletal system development, protein targeting to ER, extracellular structure organization. Pathways were enriched in Retrograde endocannabinoid signaling, Huntington's disease, Alzheimer's disease, and Oxidative phosphorylation.

### Clinical correlation analyses based on TCGA and GTEx data

**Figure 10:**
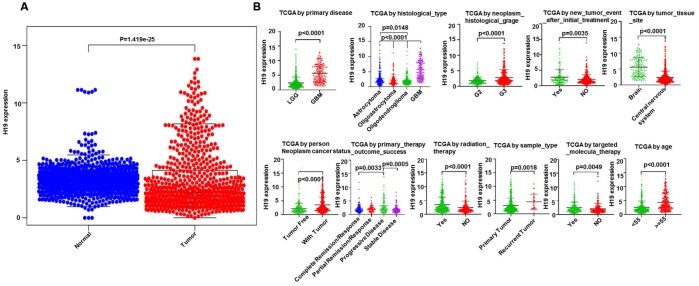
**Clinical correlation analysis between H19 and glioma patients.** (**A**) The expression level of H19 in glioma as analyzed via Limma and beeswarm package. (**B**) correlation analysis between H19 differential expression and clinical features according to TCGA.

**Figure 11:**
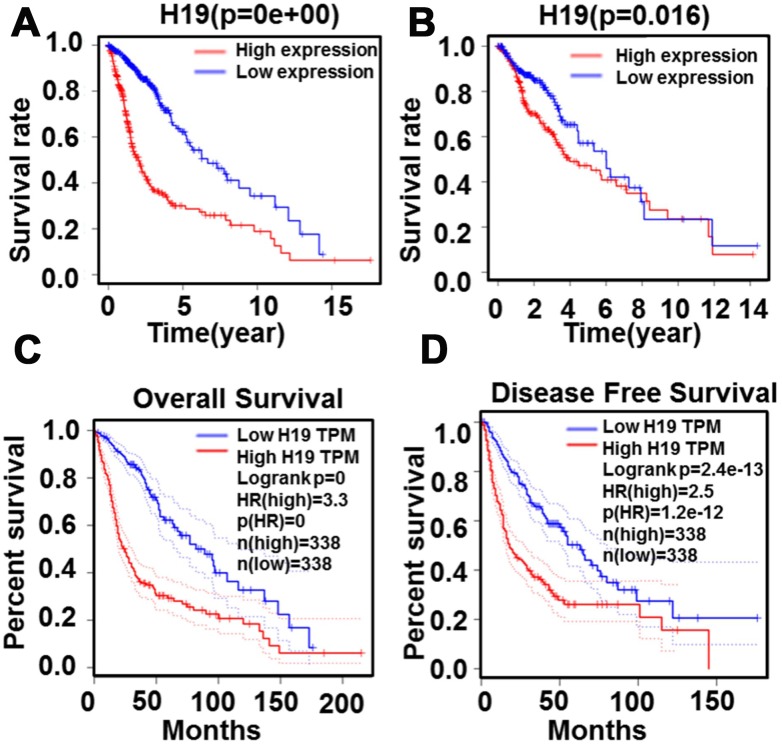
**Relationship between H19 expression levels and survival rate in glioma as analyzed by Kaplan-Meier.** (**A**) and (**B**) The relationship between survival rate and H19 expression level. (**C**) The relationship between overall survival and H19 expression level (**D**) The relationship between disease free survival and H19 expression level.

Based on Limma and beeswarm package analyses, we discovered that H19 was clearly up-regulated in gliomas (Figure 10A). Analysis of 667 samples from TCGA exhibited that H19 differential expression was noticeably associated with primary disease, histological type, histologic grade, new tumor event, tumor tissue site, cancer status, primary therapy outcome, radiation therapy, sample type, targeted molecular therapy and age (Figure 10B). Additionally, traits significantly unrelated to the expression of H19 were shown in Supplementary Figure 1. Survival analysis exhibited that low expression of H19 had a better prognosis (Figure 11), which was in line with the result from GEPIA analysis. These results demonstrated that H19 could be used as an indicator of progression and prognosis in patients with glioma, and high H19 expression indicated poor therapeutic effect. However, radiotherapy could upregulate H19 expression, indicating that radiotherapy might not be suitable for patients with glioma.

To further confirm our conjecture, univariate cox analysis was employed (Samples included LGG and GBM). The analysis unearthed that H19 was a high-risk factor (HR=2.60; 95% CI=2.18-3.09). Besides, primary disease, age, histological type, cancer status and radiation therapy were all high-risk factors. Then multivariate analysis was performed, and it was found that among these factors, H19 (HR=1.71; 95% CI=1.37-2.13; *P* <0.0001) remained independently related to overall survival, suggesting that H19 could be an independent prognostic factor for glioma patients. Interestingly, radiation therapy (HR=0.56; 95% CI=0.38-0.82; *P* =0.003) became a low-risk prognostic factor in multivariate analysis (Table 1).

**Table 1:** Univariate analysis and multivariate analysis of prognostic factors in 582 cases of glioma (LGG + GBM).

**Parameter**	**Univariate analysis**			**Multivariate analysis**		
	**HR**	**95% CI**	**P**	**HR**	**95% CI**	**P**
Primary_disease	8.98	6.65-12.13	<0.001	1.20	0.70-2.6	0.517
Age	1.07	1.05-1.09	<0.001	1.05	1.03-1.06	<0.001
H19	2.60	2.18-3.09	<0.001	1.71	1.37-2.13	<0.001
Histological_type	2.40	2.05-2.80	<0.001	1.65	1.33-2.06	<0.001
Cancer_status	16.32	7.68-34.66	<0.001	11.59	5.38-25.00	<0.001
Radiation_therapy	1.94	1.38-2.72	<0.001	0.56	0.38-0.82	0.003
Sample_type	1.58	0.99-2.50	0.054			
Gender	1.17	0.89-1.54	0.27			

LGG: low grade glioma; GBM: glioblastoma multiforme; Bold values indicate *P*<0.05; HR: hazard ratio; CI: confidence interval.

To further verify the above results, we continued to include more clinicopathological features for analyses. We obtained 416 cases with 11 clinical features and 352 cases with 18 clinical features (samples were all LGG), and the results illustrated that H19 could be used as an independent high-risk prognostic factor. Interestingly, the multivariate analysis presented that radiation therapy (*P*=0.396) and targeted molecular therapy (*P*=0.182) had no significant correlation with overall survival (Table 2). Afterwards further validation analysis on 352 patients was conducted, and the results demonstrated that H19 was an independent prognostic factor with high risk. Interestingly, radiation therapy (*P*=0.226) and targeted molecular therapy (*P*=0.202) showed no significant correlation with overall survival in the multivariate analysis (Table 3). These results revealed that H19 could be an independent prognostic factor for glioma patients. In addition, radiation therapy and targeted molecular therapy could not clearly improve the overall survival of LGG patients, and radiation therapy changed from a high risk to a low risk factor, possibly because radiation therapy only had good therapeutic effect on GBM patients.

**Table 2:** Univariate analysis and multivariate analysis of prognostic factors in 416 cases of glioma (LGG).

**Parameter**	**Univariate analysis**			**Multivariate analysis**		
	**HR**	**95% CI**	**P**	**HR**	**95% CI**	**P**
Age	1.06	1.04-1.08	<0.001	1.05	1.04-1.07	<0.001
New_tumor_event	3.86	2.45-6.09	<0.001	2.19	1.34-3.47	<0.001
Cancer_status	15.15	5.56-41.2	<0.001	12.62	4.40-36.17	<0.001
Grade	3.14	2.05-4.78	<0.001	2.19	1.37-3.49	0.001
H19	1.86	1.41-2.45	<0.001	1.60	1.18-2.18	0.002
Radiation_therapy	2.06	1.28-3.30	0.003	0.80	0.47-1.34	0.396
Histological_type	1.30	1.04-1.63	0.021	1.42	1.12-1.81	0.004
Targeted_molecular_therapy	1.32	0.88-1.97	0.182			
Sample_type	1.19	0.58-2.47	0.633			
Gender	0.93	0.63-1.37	0.726			

LGG: low grade glioma; Bold values indicate *P*<0.05. HR: hazard ratio; CI: confidence interval.

**Table 3:** Univariate and multivariate analyses of prognostic factors in 352 cases of glioma (LGG).

**Parameter**	**Univariate analysis**			**Multivariate analysis**		
	**HR**	**95% CI**	**P**	**HR**	**95% CI**	**P**
Age	1.06	1.04-1.07	<0.001	1.05	1.04-1.07	<0.001
New_tumor_event	4.36	2.61-7.30	<0.001	1.54	0.85-2.80	0.155
Cancer_status	12.1	4.43-33.03	<0.001	4.11	1.41-12.00	0.010
Grade	3.18	1.98-5.09	<0.001	2.12	1.27-3.54	0.004
Primary_therapy	0.41	0.31-0.53	<0.001	0.56	0.41-0.80	0.001
H19	2.00	1.44-2.78	<0.001	1.54	1.09-2.17	0.014
Histological_type	1.41	1.10-1.81	0.007	1.57	1.19-2.08	0.001
Radiation_therapy	1.84	1.11-3.07	0.019	0.70	0.39-1.25	0.226
Targeted_molecular_therapy	1.33	0.86-2.06	0.202			
Laterality	0.87	0.70-1.08	0.209			
Headache_history	0.77	0.49-1.22	0.270			
Motor_movement_changes	1.32	0.80-2.18	0.277			
Seizure_history	0.86	0.56-1.31	0.470			
Sensory_changes	1.13	0.65-1.95	0.668			
Sample_type	1.10	0.50-2.40	0.815			
Gender	1.02	0.67-1.55	0.929			
Mental_status_changes	1.01	0.61-1.69	0.966			
Visual_changes	1.00	0.53-1.88	0.992			

LGG: low grade glioma; Bold values indicate *P*<0.05. HR: hazard ratio; CI: confidence interval.

Univariate analysis via logistic regression exhibited that H19 was associated with clinical pathological characteristics of poor prognosis (Table 4). The upregulation of H19 in gliomas was clearly interrelated with primary disease (OR=12.20, for LGG vs. GBM), age (OR=4.55, for <55 vs.≥55), cancer status (OR=2.20, for tumor free vs. with tumor), radiation therapy (OR=2.47, for NO vs. YES), grade (OR=2.24, for G2 vs.G3) and new tumor event (OR=1.93, for NO vs. YES) (all *P*<0.05). These results illustrated that patients with high expression of H19 tended to have a worse prognosis than those with low expression of H19.

**Table 4:** H19 expression associated with clinical pathological characteristics (logistic regression).

**Clinical characteristics**	**Total (N)**	**Odds ratio in H19 expression**	**p-Value**
Primary_disease (LGG vs.GBM)	677	12.20 (7.50-20.92)	<0.001
Age (<55 vs.≥55)	677	4.55 (3.20-6.54)	<0.001
Sample_type (primary tumor vs.recurrent tumor)	677	2.06 (0.94-4.88)	0.082
Gender (female vs.male)	675	1.07 (0.79-1.45)	0.664
Cancer_status (tumor free vs. with tumor)	630	2.20 (1.58-3.08)	<0.001
Radiation_therapy (NO vs.YES)	615	2.47 (1.75-3.52)	<0.001
Grade (G2 vs.G3)	514	2.24 (1.57-3.19)	<0.001
Laterality (left vs.right)	503	1.02(0.72-1.45)	0.893
Seizure_history (NO vs.YES)	480	0.82 (0.57-1.19)	0.298
Headache_history (NO vs.YES)	465	1.21 (0.83-1.77)	0.318
Targeted_molecular_therapy (NO vs.YES)	460	1.84 (1.27-2.68)	0.001
Mental_status_changes (NO vs.YES)	459	1.40 (0.91-2.14)	0.125
Motor_movement_changes (NO vs.YES)	457	1.27 (0.83-1.97)	0.263
Sensory_changes (NO vs.YES)	454	1.39 (0.84-2.33)	0.200
New_tumor_event (NO vs.YES)	446	1.93 (1.30-2.90)	0.001
Histological_type (Oligodendroglioma vs. Astrocytoma)	385	1.75(1.15-2.70)	0.009
Family_history_of_cancer (NO vs.YES)	342	0.95 (0.62-1.47)	0.824
Primary_therapy (complete remission vs.progressive disease)	240	1.81 (1.09-3.05)	0.024

^a^ Categorical dependent variable, greater or less than the median expression level.

**Figure 12:**
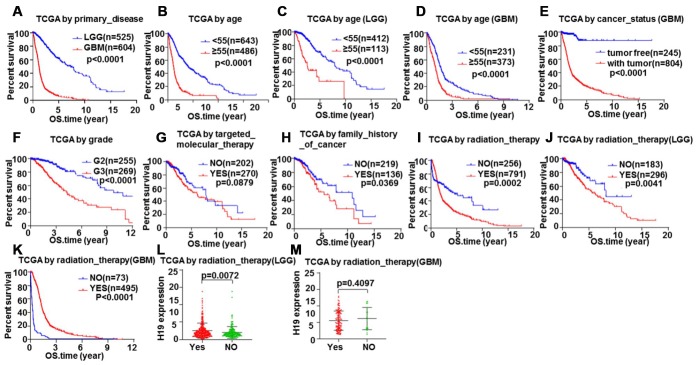
**Glioma samples survival analysis validation by Kaplan-Meier.** Correlation between total survival and (**A**) Primary disease, (**B**–**D**) Age, (**E**) Cancer status, (**F**) Grade, (**G**) Targeted molecular therapy, (**H**) Family history of cancer, (**I**–**K**) Radiation therapy. The relationship between H19 expression level and radiotherapy in (**L**) LGG and (**M**) GBM.

In order to further verify the above inferences, survival analysis was conducted on 1148 glioma patients (LGG and GBM) by Kaplan-Meier plotter. We discovered that patients with LGG had a longer overall survival than those with GBM (Figure 12A, *P*<0.05). Patients younger than 55 have a longer survival time than those older than 55 (Figure 12B–12D, *P*<0.05). The tumor-free survival time was longer than that with tumor (Figure 12E, *P*<0.05). Patients with grade 2 had a longer survival time than patients with grade 3 (Figure 12F, *P*<0.05). Targeted molecular therapy was not correlated with survival time (Figure 12G, *P*>0.05). Patients without a family history of cancer had a longer survival than those with a family history of cancer (Figure 12H, *P*<0.05). Patients without radiotherapy have a longer survival time than patients with radiotherapy (Figure 12I, *P*<0.05), which was consistent with that in LGG patients, whereas patients who received radiotherapy in GBM patients had a longer survival time than those who did not (Figure 12J, 12K, *P*<0.05). Meanwhile, we found that the expression level of H19 in patients who received radiotherapy in LGG was higher than those who did not (Figure 12L, *P*<0.05), and the opposite result was found in GBM, but not statistically significant (Figure 12M, *P*>0.05). These results exhibited that targeted molecular therapy had no effect on the survival time of glioma patients. Primary disease, age, cancer status, grade and radiation therapy had effects on the prognosis of patients with glioma, and radiation therapy had opposite therapeutic effects on LGG and GBM patients, suggesting that the expression of H19 could act as an indicator molecule.

### GSEA enrichment analysis of H19

**Figure 13:**
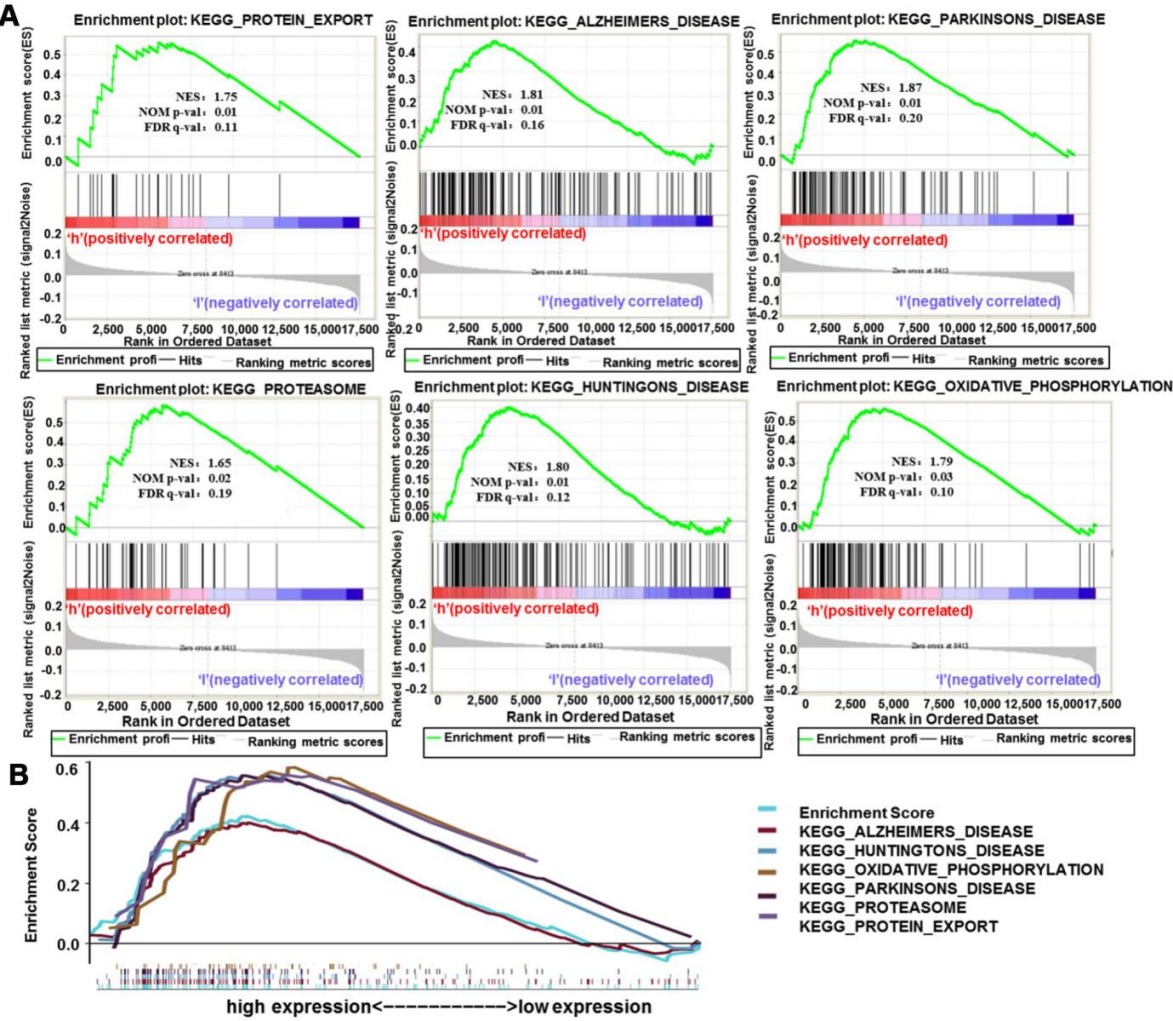
**Enrichment plots from GSEA analysis.** (**A**) GSEA analysis showed that protein export, Alzheimer's disease, Parkinson's disease, proteasome, Huntington's disease and oxidative phosphorylation were differentially enriched in H19 high expression phenotype. (**B**) The integrated GSEA analysis. Abbreviations: GSEA, gene set enrichment analysis; ES, enrichment score; NES, normalized ES; NOM p-val, normalized p-value.

To further analyze the biological functions exerted by H19 in glioma, GSEA enrichment analysis was performed on high and low expression datasets of H19. The result uncovered significant differences (FDR <0.25, NOM p-val <0.05) in the enrichment of MSigDB Collection (c2.cp.biocarta and h.all. v6.1. symbols). Then, the most markedly enriched signaling pathways were screened according to their NES (Figure 13 and Table 5). Figure 13 illustrated that protein export, Alzheimer's disease, Parkinson's disease, proteasome, Huntington's disease and oxidative phosphorylation were differentially concentrated in H19 high expression phenotype.

**Table 5:** Gene sets enriched in high expression phenotype.

**Gene set name**	**NES**	**NOM p-val**	**FDR q-val**
KEGG_PROTEIN_EXPORT	1.75	0.01	0.11
KEGG_ALZHEIMERS_DISEASE	1.81	0.01	0.16
KEGG_PARKINSONS_DISEASE	1.87	0.01	0.20
KEGG_PROTEASOME	1.65	0.02	0.19
KEGG_HUNTINGTONS_DISEASE	1.80	0.02	0.12
KEGG_OXIDATIVE_PHOSPHORYLATION	1.79	0.03	0.10
KEGG_OXIDATIVE_PHOSPHORYLATION	1.79	0.03	0.10

NES: normalized enrichment score; NOM: nominal; FDR: false discovery rate. Gene sets with NOM p-val<0.05 and FDR q-val<0.25 are considered as significant.

### Correlation analyses between H19 and immune infiltration

**Figure 14:**
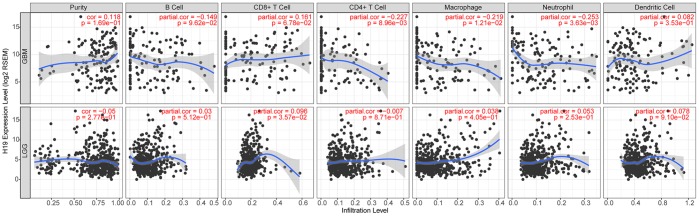
**Correlation analysis between the expression level of H19 and immune infiltration.**

**Figure 15:**
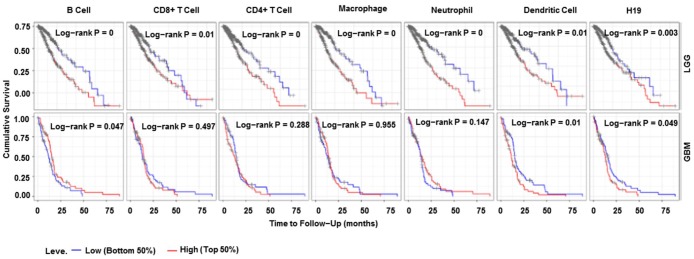
**Immune cell infiltration survival curve.** They are K-M survival curves based on top and bottom sample partitions with 50% and 50% immune penetration, respectively. Red means high degree of infiltration and blue means low degree of infiltration. *P*< 0.05 was considered significant, and *P* < 0.0001 was represented by 0. Abbreviations: K-M, Kaplan-Meier plotter.

According to TIMER data analysis, H19 was negatively correlated with Neutrophil, CD4+ T Cell and Macrophage in GBM, while positively related to CD8+ T Cell in LGG (Figure 14 and Table 6). Univariate COX survival analysis demonstrated that six types of immune cells and H19 markedly affected the survival time of patients with LGG, while only H19, Dendritic cells and B cells influenced the survival time of patients with GBM (Figure 15 and Table 7). Multivariate COX survival analysis elucidated that age, macrophage and H19 could be independent prognostic factors for LGG (Table 8), and age, CD4_Tcell, Dendritic and H19 for GBM (Table 9). Besides, it was found that the copy number variations of H19 could notably affect the infiltration level of glioma immune cells (Supplementary Figure 2), but could not affect the mRNA level of H19 (Supplementary Figure 3). These results indicated that H19 could affect the immune infiltration level by copy number variations, thereby influencing the prognosis of patients with glioma. Collectively, H19 has potential reference value for glioma remission and immunotherapy.

**Table 6:** H19 expression associated with immune infiltration level in glioma (partial Spearman’s correlation).

**Cancer**	**Variable**	**Partial.cor**	**P**
GBM	Neutrophil	-0.25	0.00
GBM	CD4+ T Cell	-0.23	0.01
GBM	Macrophage	-0.22	0.01
LGG	CD8+ T Cell	0.10	0.04
GBM	CD8+ T Cell	0.16	0.07
LGG	Dendritic Cell	0.08	0.09
GBM	B Cell	-0.15	0.10
GBM	Purity	0.12	0.17
LGG	Neutrophil	0.05	0.25
LGG	Purity	-0.05	0.28
GBM	Dendritic Cell	0.08	0.35
LGG	Macrophage	0.04	0.40
LGG	B Cell	0.03	0.51
LGG	CD4+ T Cell	-0.01	0.87

**Table 7:** Univariate analysis of the correlation of H19 expression and immune infiltrates with OS among glioma patients.

**Cancer**	**Variable**	**P**
LGG	Neutrophil	0.00
LGG	Macrophage	0.00
LGG	B Cell	0.00
LGG	CD4+ T Cell	0.00
LGG	Dendritic Cell	0.00
LGG	H19	0.00
LGG	CD8+ T Cell	0.01
GBM	Dendritic Cell	0.01
GBM	B Cell	0.05
GBM	H19	0.05
GBM	Neutrophil	0.15
GBM	CD4+ T Cell	0.29
GBM	CD8+ T Cell	0.50
GBM	Macrophage	0.95

**Table 8:** Multivariate analysis of the correlation of H19 expression and immune infiltrates with OS among LGG patients.

	**coef**	**HR**	**95% CI**	**p.value**	**sig**
Age	0.05	1.05	1.04-1.07	0.00	***
gendermale	0.22	1.25	0.85-1.84	0.26	
raceBlack	15.8	7296302.15	0.00-Inf	1.00	
raceWhite	16.05	9354676.89	0.00-Inf	1.00	
B_cell	0.38	1.46	0.01-452.58	0.90	
CD8_Tcell	3.52	33.68	0.038-29527.813	0.31	
CD4_Tcell	-1.57	0.21	0.00-410.03	0.69	
Macrophage	5.31	201.27	4.35-9321.50	0.01	**
Neutrophil	-3.07	0.05	0.00-91.95	0.43	
Dendritic	1.49	4.45	0.11-180.20	0.43	
H19	0.10	1.10	1.04-1.17	0.00	**

**Table 9:** Multivariate analysis of the correlation of H19 expression and immune infiltrates with OS among GBM patients.

	**coef**	**HR**	**95% CI**	**p.value**	**sig**
Age	0.03	1.03	1.01-1.05	0.00	**
gendermale	0.10	1.10	0.71-1.73	0.67	
raceBlack	-0.24	0.78	0.16-3.88	0.77	
raceWhite	-0.59	0.55	0.16-1.95	0.36	
B_cell	-0.21	0.81	0.14-4.83	0.82	
CD8_Tcell	0.21	1.24	0.36-4.24	0.74	
CD4_Tcell	4.83	125.40	10.68-147.23	0.00	***
Macrophage	1.57	4.80	0.43-53.96	0.20	
Neutrophil	-2.19	0.11	0.01-1.83	0.13	
Dendritic	1.14	3.12	1.27-7.64	0.01	*
H19	0.11	1.12	1.03-1.21	0.01	**

## DISCUSSION

Glioma is a type of tumor derived from glial cells in the spine or brain [17], accounting for about 80% of malignant brain tumors [18]. Current therapy for glioma mainly depends on surgery, and postoperative supplemented radiotherapy and chemotherapy [19]. Due to the invasive growth character of glial tumors, they cannot be completely resected by surgery [20]. Despite in-depth studies, the prognosis of GBM is poor, and the average survival of patients with GBM is still less than 2 years [21]. Therefore, effective biomarkers for early diagnosis of glioma are conductive to the treatment and prognosis of patients.

Recently, there have been many reports on the expression and functions of IncRNA H19 in cancers [22–24], including glioma [25]. To our knowledge, the possible prognostic value of H19 in gliomas remains largely to be explored and the underlying role of H19 in gliomas is the focus of this current study. As reported in related literature, H19 has a high expression in glioma tissues [16, 26]. In the present study, we found that H19 was highly expressed in glioma tissues by Oncomine database analyses. The result is consistent with the finding in the previous study, revealing that H19 might act as a crucial regulatory role in glioma progression. In addition, it was found that the expression of H19 increased with the increase of malignancy in the clinic correlation analyses, which was also confirmed in another study [27]. The result unearthed that H19 might sever as a possible molecular marker for predicting the degree of glioma malignancy.

In the KEGG analysis, DEGs were concentrated in PI3K−Akt signaling pathway, TGF−beta signaling pathway and tumor-related signaling pathway. Importantly, PI3K-AKT signaling pathway has been repeatedly reported as an important signaling pathway involved in the regulation of various aspects of cancers, such as tumor growth and metastasis [28–30]. TGF-beta signaling pathway also plays a key role in metazoan biology, and its misregulation can lead to tumor development [31]. To some extent, these pathways also indicate that H19 is closely related to glioma.

In the clinical correlation analyses, we found that radiotherapy had a good therapeutic effect on GBM patients, but a poor effect on LGG patients. Meanwhile, the expression level of H19 could act as an indicator molecule to indicate the good or bad effect of radiotherapy. In the treatment of glioma, radiotherapy has been proven suitable for GBM [32, 33]. Nonetheless, whether radiotherapy is suitable for patients with LGG is worth further exploration.

In conclusion, our work demonstrated that the expression level of H19 increased with the increase of malignancy. The expression level of H19 in serum-free cultured glioma cells was higher than that in serum cultured ones. Radiation therapy had a good therapeutic effect in GBM patients, while the effect was not good in LGG patients. Meanwhile, the expression level of H19 could act as an indicator molecule indicating the effect of radiotherapy. These findings are expected to provide a theoretical basis for the development of biomarkers in the diagnosis and immune treatment of patients with glioma. Although we explored the relationship between H19 expression and glioma via bioinformatics, this study lacks related validation *in vivo* and *in vitro*. Hence, we will further study the function and mechanism of H19 by a range of cell lines, tissues and animal experiments after we have favorable design and preparation.

## MATERIALS AND METHODS

### Oncomine database analysis

Oncomine (www.oncomine.org), currently the world's largest oncogene chip database and integrated data mining platform, contains 715 gene expression data sets from 86,733 cancer and normal tissues [34]. The expression level of H19 in glioma and its correlations with the degree of glioma malignancy and the clinical characteristics of glioma patients were analyzed in the Oncomine database. This analysis drew on five glioma studies, including Rickman Brain, Liang Brain, Murat Brain, Lee Brain, Sun Brain, and Bredel Brain glioma studies [35–40]. The expression level of H19 in glioma tissues was evaluated relative to that in normal tissue, as well as its expression in tumor tissues with different malignant degrees. *P* <0.01, Gene Rank <Top 10% was considered significant. Outlier analysis has been widely applied in gene expression data for the discovery of cancer-associated genes [41]. In this study, outlier analysis of H19 was performed in the Oncomine database.

### GEPIA database analysis

GEPIA is a newly developed interactive web server that uses standard processing pipelines to analyze RNA sequencing data based on 9,736 tumor samples and 8,587 normal samples from the TCGA and GTEx databases [42]. GEPIA provides customizable functions such as tumor / normal differential expression analysis, analysis based on cancer type or pathological stage, patient survival analysis, similar genetic testing, correlation analysis and dimensional reduction analysis.

### Expression level of H19 in normal tissues

GTEx were downloaded from UCSC Xena project [43] (http://xena.ucsc.edu/). Data was as follows: TOIL RSEM fpkm (n=7,862) and phenotype (n=9,783). The Ensembl number of the gene in RNAseq data was transformed into the Official Symbol of the gene through your own perl [44] script. Then, based on GTEx RNAseq and phenotype data, perl script was used to extract the expression level of H19 in different normal organs of men and women, and visualization was conducted based on GitHub [45] website R package: gganatogram [46] and ggpubr.

### Glioma data sets and data processing

To solve the problem that too little normal sample data may lead to inaccurate differential expression analysis, UCSC Xena project was employed. 697 glioma samples (LGG+GBM) were download from TCGA database, and five paracancer samples and 1152 normal brain tissues were download from GTEx database. All original RNA-seq data were recalculated based on standard channels to minimize differences from different sources.

### Screening of DEGs and GO and KEGG pathway analyses

Based on glioma data integrated from UCSC Xena, DEGs were screened by limma [47] package (screening criteria: |logFC| > 2, FDR<0.05) and then their heatmap was plotted by pheatmap [48] package. Then GO and KEGG pathway analyses were performed on the screened DEGs: GO analysis included biological process (BP), cell component (CC) and molecular function (MF). KEGG (http://www.genome.jp/) was a systematic analysis method for gene function used to discover biological regulatory pathways. First, the Official Symbol of DEGs was converted into gene ID through org.Hs.eg.db package, clusterProfiler package [49] was used for GO and KEGG pathway analyses, and GOplot package [50] was employed for cluster analyses. Moreover, it was considered to have significant difference only when both *P* value and q value were less than 0.05.

### Clinical correlation analyses between H19 expression and glioma patients

The relevant clinical information of glioma patients, namely the phenotype of TCGA LGG and GBM (n=1148), was download from UCSC Xena database. The expression value of H19 in glioma patients with clinical information were integrated to obtain 677 patients via perl script, and the expression difference of H19 in discrete variables of clinical information was analyzed by using Prism 8 software. Finally, among these 677 patients, we eliminated some patients with incomplete clinical information and obtained 582 patients for COX analysis.

### GSEA enrichment analysis

GSEA is a computational method that determines whether a priori defined set of genes shows statistically significant and concordant differences between two biological states [51]. In this study, an ordered list of all genes was first generated by GSEA according to their correlations with H19 expression. GSEA was then used to elucidate the significant survival difference observed between high- and low-H19 groups. Gene set permutations were performed 1000 times for each analysis. The expression level of H19 was used as a phenotype label. The nominal *P* value and normalized enrichment score (NES) were used to sort the pathways enriched in each phenotype.

### Immune infiltration analysis

TIMER [52] (https://cistrome.shinyapps.io/timer/) is a comprehensive resource for systematical analysis of immune infiltrates across diverse cancer types. The abundances of six immune infiltrates (B cells, CD4+ T cells, CD8+ T cells, Neutrphils, Macrophages and Dendritic cells) were estimated by our statistical method, which was validated using pathological estimations. This web server allows users to input function-specific parameters, with resulting figures dynamically displayed and makes it convenient to access the tumor immunological, clinical, and genomic features. We used the TIMER “gene” module to analyze the correlations between H19 expression level and glioma immune infiltration level. Kaplan-Meier method was used to analyze the influence of H19 and immune cell infiltration on the prognosis of glioma patients (GBM+LGG). Meanwhile, clinical factors were included to construct a multivariate COX proportional risk model. Finally, SCNA module was used to analyze the relationship between H19 copy number variations in different somatic cells and infiltration level of glioma. The correlations between copy number variations and mRNA level of H19 were analyzed by cBioportal (http://www.cbioportal.org/) [53] database.

## Supplementary Material

Supplementary Figures

Supplementary Table 1
