# Supplementary material for: Expression and prognostic value of long non-coding RNA H19 in glioma via integrated bioinformatics analyses
Source: Aging (Albany NY). 2020 Feb 20;12(4):3407–30. doi: 10.18632/aging.102819 (PMC7066912; doi:10.18632/aging.102819)
Supplement: Supplementary Figures [file aging-12-102819-s002..pdf]

SUPPLEMENTARY FIGURES

Supplementary Figures

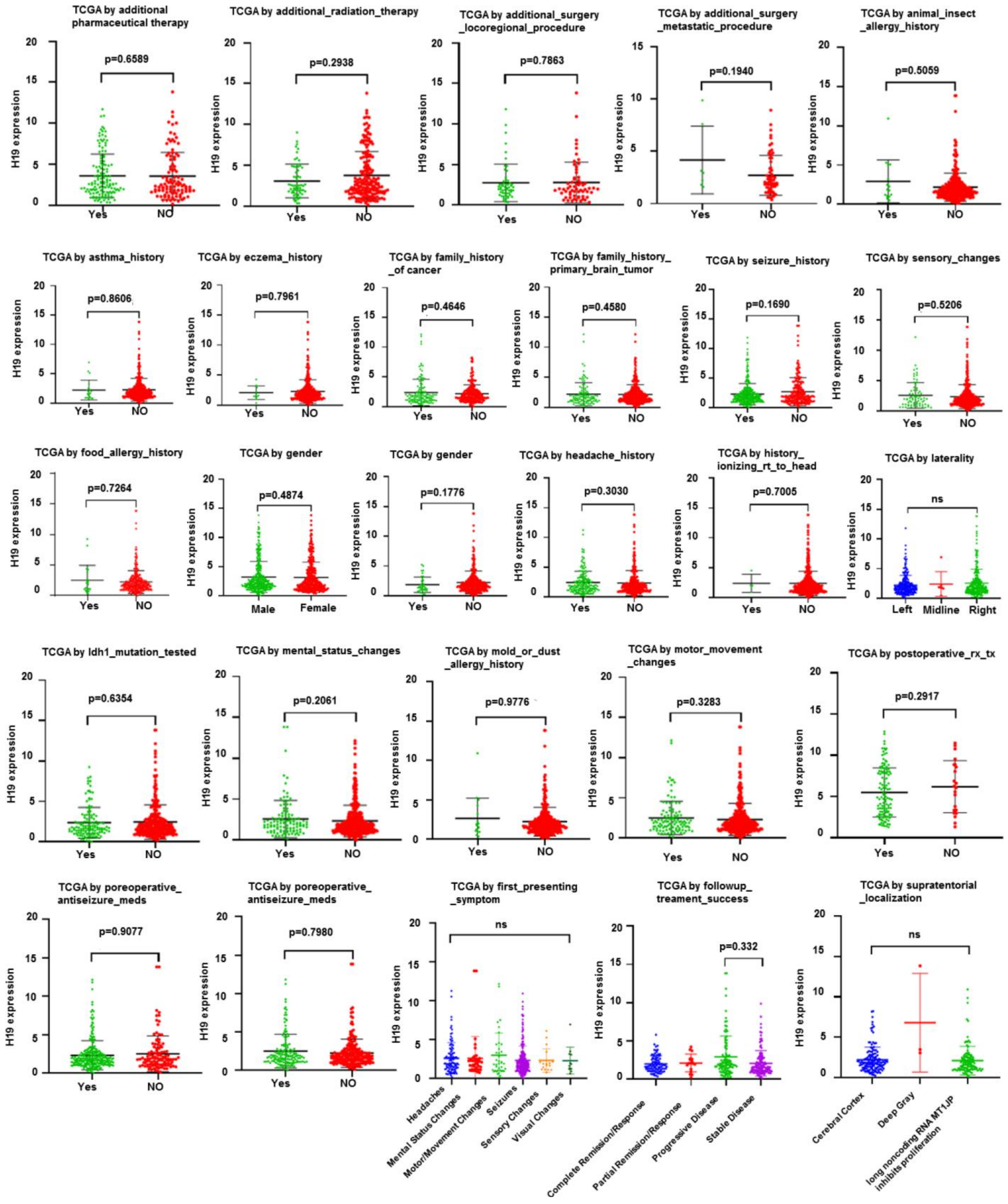

Supplementary Figure 1. No significant correlation between H19 expression and clinical traits.

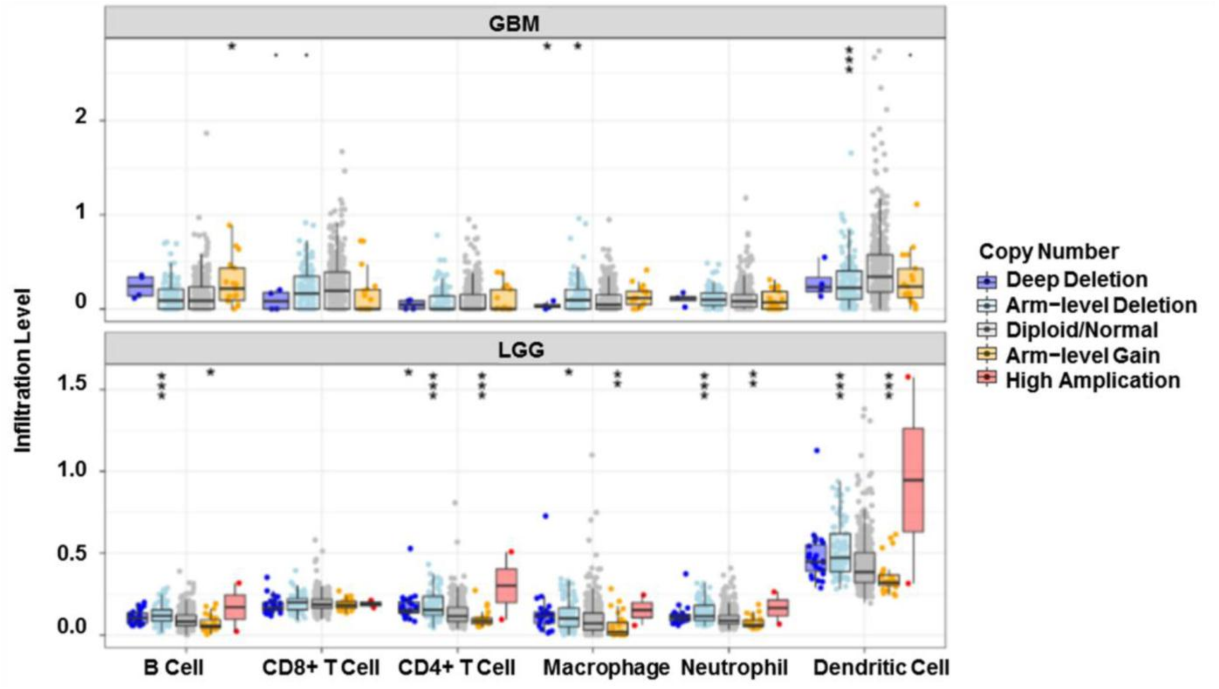

Supplementary Figure 2. The relation between H19 copy number variation and infiltration level. *, *P*<0.05; **, *P*<0.01; ***, *P*<0.001. Abbreviation: LGG, low grade glioma; GBM, glioblastoma.

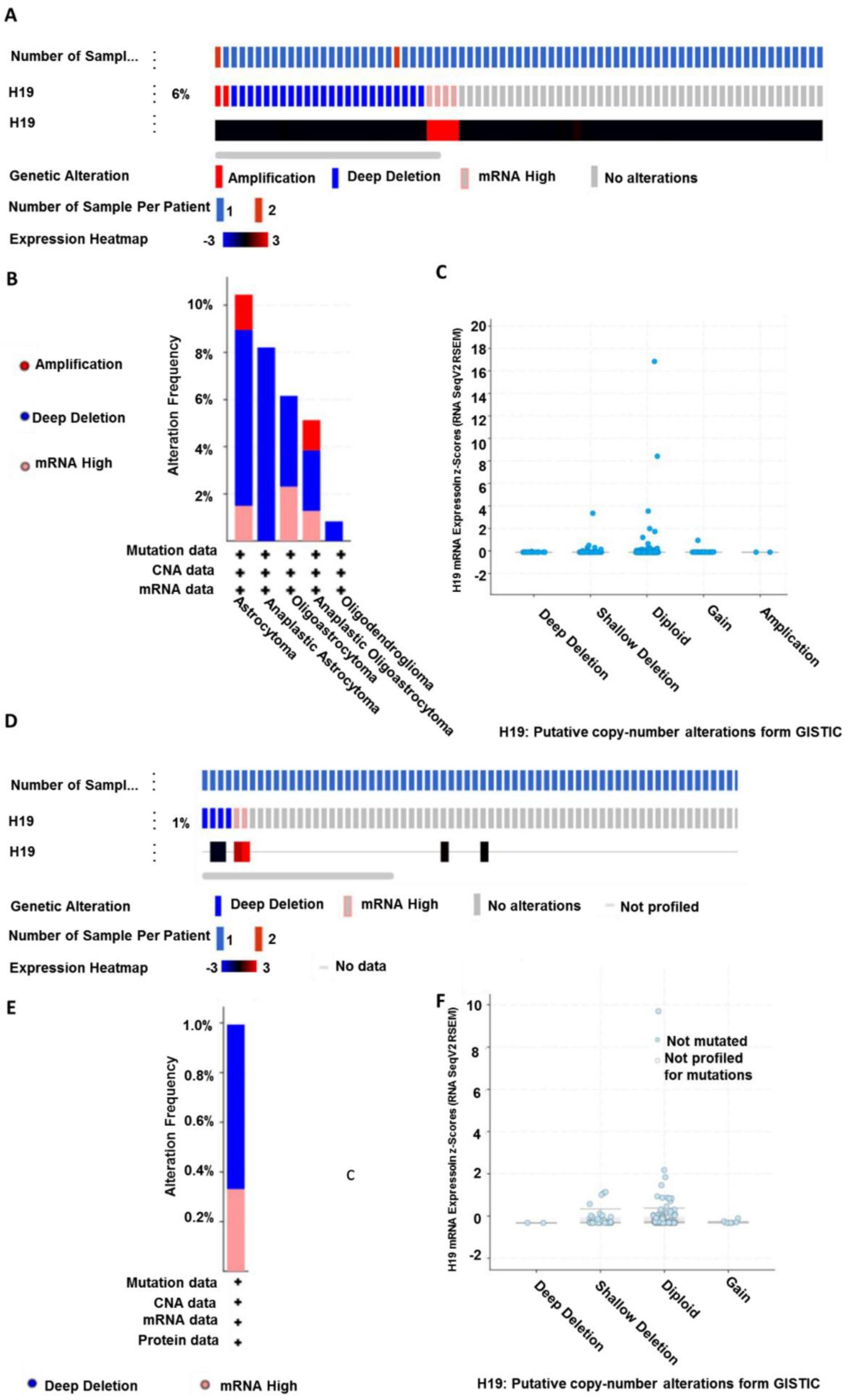

Supplementary Figure 3. The relationship between the copy number of H19 and the mRNA level of H19. The copy number of H19 did not affect the mRNA level of H19.
