# Supplementary material for: Expression and prognostic value of long non-coding RNA H19 in glioma via integrated bioinformatics analyses
Source: Aging (Albany NY). 2020 Feb 20;12(4):3407–30. doi: 10.18632/aging.102819 (PMC7066912; doi:10.18632/aging.102819)
Supplement: Supplementary Table 1 [file aging-12-102819-s001..docx]

**Supplementary Table 1. Gene expression value, fold change, and *P*-value in DEGs analysis.**

| Gene | ConMean | TreatMean | LogFC | *P*-value | FDR |
| --- | --- | --- | --- | --- | --- |
| RPL23A | 10.490581 | 7.7743847 | -2.716197 | 3.36E-274 | 6.12E-273 |
| CALB2 | 6.6324608 | 2.9672843 | -3.665177 | 1.00E-235 | 5.27E-235 |
| GLI4 | 6.1066807 | 4.042133 | -2.064548 | 1.02E-270 | 1.44E-269 |
| KRT1 | 2.7007934 | 0.3263735 | -2.37442 | 1.87E-238 | 1.02E-237 |
| MT1L | 5.3789117 | 2.3846097 | -2.994302 | 3.75E-259 | 3.08E-258 |
| MITD1 | 6.0258375 | 3.8935945 | -2.132243 | 2.50E-280 | 1.13E-278 |
| SPCS2 | 8.1539581 | 5.9578714 | -2.196087 | 8.22E-276 | 1.77E-274 |
| MT1A | 4.3930581 | 1.1395449 | -3.253513 | 2.21E-247 | 1.40E-246 |
| COX17 | 7.3893456 | 4.6708173 | -2.718528 | 7.96E-279 | 2.66E-277 |
| TMEM52 | 3.1041248 | 0.7528844 | -2.35124 | 4.34E-263 | 4.12E-262 |
| RBM15B | 4.7403738 | 6.8821964 | 2.1418226 | 4.49E-285 | 3.32E-282 |
| NCAPG | 0.8152788 | 3.5614386 | 2.7461598 | 1.68E-241 | 9.59E-241 |
| UPP1 | 6.9338548 | 4.4258369 | -2.508018 | 3.73E-224 | 1.70E-223 |
| TNNT1 | 5.6164931 | 2.1947952 | -3.421698 | 2.27E-244 | 1.36E-243 |
| H6PD | 4.1799102 | 6.5492137 | 2.3693035 | 2.65E-284 | 7.09E-282 |
| WFDC1 | 5.2210523 | 3.061605 | -2.159447 | 2.89E-228 | 1.38E-227 |
| CBL | 4.1073846 | 6.7672159 | 2.6598312 | 6.47E-284 | 1.23E-281 |
| OGDHL | 6.6679953 | 3.9724991 | -2.695496 | 6.92E-211 | 2.72E-210 |
| PRRT1 | 7.7733118 | 4.9309091 | -2.842403 | 3.25E-256 | 2.48E-255 |
| SLC7A11 | 4.3637649 | 6.4668408 | 2.1030759 | 6.20E-214 | 2.52E-213 |
| NCAN | 6.1683106 | 9.8888677 | 3.7205571 | 1.25E-235 | 6.55E-235 |
| MGA | 3.6840947 | 5.6967378 | 2.012643 | 9.26E-264 | 9.09E-263 |
| CAPN3 | 7.4456888 | 5.2620192 | -2.18367 | 3.06E-159 | 7.94E-159 |
| HOOK3 | 4.240476 | 6.5871243 | 2.3466482 | 1.80E-278 | 5.66E-277 |
| RGS20 | 5.6607726 | 3.6142676 | -2.046505 | 3.65E-175 | 1.06E-174 |
| ABCA1 | 3.4244459 | 7.3745871 | 3.9501412 | 4.60E-280 | 1.93E-278 |
| S100A12 | 2.8445398 | 0.6534217 | -2.191118 | 8.84E-167 | 2.42E-166 |
| CSPG4 | 4.2454424 | 7.5373146 | 3.2918722 | 4.68E-263 | 4.44E-262 |
| B3GNT4 | 3.6567578 | 1.4260573 | -2.2307 | 1.79E-231 | 8.88E-231 |
| WIF1 | 5.4941309 | 2.0242052 | -3.469926 | 1.34E-169 | 3.73E-169 |
| XPO4 | 3.3945907 | 5.3976152 | 2.0030245 | 2.85E-277 | 7.64E-276 |
| DDR1 | 7.5634546 | 10.970046 | 3.4065915 | 1.13E-275 | 2.40E-274 |
| PRB2 | 2.2623175 | 0.1263581 | -2.135959 | 1.61E-165 | 4.37E-165 |
| ZNF487 | 4.5791749 | 2.3399569 | -2.239218 | 2.10E-282 | 1.75E-280 |
| TIMP4 | 4.5811425 | 6.6471987 | 2.0660562 | 8.26E-147 | 1.99E-146 |
| RBM12 | 4.7615973 | 6.8907623 | 2.129165 | 6.92E-281 | 3.54E-279 |
| IGDCC4 | 3.5841381 | 5.6172234 | 2.0330853 | 1.32E-217 | 5.58E-217 |
| ANXA3 | 4.9370841 | 2.2585486 | -2.678535 | 1.40E-258 | 1.13E-257 |
| IGF1R | 4.5268031 | 6.7734349 | 2.2466318 | 2.06E-259 | 1.71E-258 |
| RPL38 | 11.220675 | 8.4069032 | -2.813772 | 4.60E-274 | 8.25E-273 |
| PRRT2 | 8.0205506 | 5.7603644 | -2.260186 | 1.83E-169 | 5.11E-169 |
| FAHD2B | 6.7479961 | 3.6797625 | -3.068234 | 5.23E-280 | 2.16E-278 |
| BDH1 | 6.9121789 | 4.394667 | -2.517512 | 9.40E-268 | 1.11E-266 |
| LRIG1 | 6.7543868 | 10.141856 | 3.3874693 | 4.50E-270 | 6.01E-269 |
| NOTCH2 | 4.3629733 | 8.1674816 | 3.8045083 | 1.13E-283 | 1.86E-281 |
| DPYSL3 | 6.729831 | 10.783982 | 4.0541505 | 2.07E-281 | 1.22E-279 |
| SERPINI1 | 8.0353556 | 5.2486291 | -2.786727 | 2.80E-183 | 8.62E-183 |
| RPL23AP82 | 6.1668778 | 4.1323914 | -2.034486 | 7.46E-276 | 1.63E-274 |
| VCAM1 | 2.7333591 | 5.6775566 | 2.9441975 | 3.26E-197 | 1.12E-196 |
| PBX1 | 5.6932084 | 7.7996929 | 2.1064845 | 2.57E-277 | 6.89E-276 |
| NDUFA3 | 9.5165052 | 5.8514327 | -3.665073 | 6.07E-277 | 1.54E-275 |
| KIF1B | 7.4019215 | 9.4611475 | 2.059226 | 6.59E-250 | 4.36E-249 |
| RPL9 | 10.662174 | 5.2532725 | -5.408901 | 1.28E-279 | 4.96E-278 |
| DCAF7 | 5.7871408 | 8.3069195 | 2.5197787 | 3.24E-285 | 3.32E-282 |
| CENPE | 0.8520564 | 3.0031737 | 2.1511173 | 5.37E-261 | 4.74E-260 |
| ZDHHC20 | 5.6530348 | 3.5271766 | -2.125858 | 1.38E-275 | 2.90E-274 |
| RILP | 5.3204221 | 3.2298593 | -2.090563 | 1.21E-276 | 2.96E-275 |
| C9orf16 | 8.6477917 | 6.1138626 | -2.533929 | 2.35E-258 | 1.89E-257 |
| CRIP1 | 5.413285 | 3.0283534 | -2.384932 | 2.60E-215 | 1.07E-214 |
| SYCE1L | 3.1063105 | 1.0867146 | -2.019596 | 6.02E-230 | 2.93E-229 |
| CRIP3 | 5.2580447 | 1.8525399 | -3.405505 | 4.84E-277 | 1.24E-275 |
| PTS | 6.7578557 | 4.2453938 | -2.512462 | 4.31E-280 | 1.83E-278 |
| PLEKHM3 | 2.529935 | 5.4545856 | 2.9246506 | 7.83E-274 | 1.37E-272 |
| ZFHX3 | 2.4455717 | 5.425283 | 2.9797113 | 1.43E-284 | 4.91E-282 |
| PBK | 0.4441098 | 3.7133499 | 3.2692401 | 1.43E-265 | 1.52E-264 |
| PXDN | 3.5449499 | 6.2072689 | 2.6623191 | 2.47E-244 | 1.47E-243 |
| CORO1C | 6.247628 | 8.5767987 | 2.3291707 | 3.70E-283 | 4.42E-281 |
| TP53INP1 | 3.3895718 | 5.598955 | 2.2093832 | 2.99E-277 | 7.98E-276 |
| ACAP1 | 5.0515446 | 1.8443684 | -3.207176 | 1.66E-277 | 4.58E-276 |
| VIPR1 | 5.1160557 | 2.9317498 | -2.184306 | 1.78E-160 | 4.66E-160 |
| RASSF2 | 6.9117434 | 9.4044111 | 2.4926678 | 3.75E-207 | 1.42E-206 |
| CNIH2 | 7.8477821 | 5.5163157 | -2.331466 | 2.64E-210 | 1.03E-209 |
| FPR3 | 0.8329707 | 3.192548 | 2.3595772 | 3.02E-216 | 1.25E-215 |
| SALL1 | 4.7060489 | 7.1108409 | 2.4047919 | 1.44E-242 | 8.38E-242 |
| CA4 | 5.6997339 | 3.5625085 | -2.137225 | 1.57E-150 | 3.86E-150 |
| FREM2 | 0.8947432 | 4.183092 | 3.2883487 | 4.74E-272 | 7.24E-271 |
| NLRP1 | 8.4772795 | 3.6171588 | -4.860121 | 1.26E-282 | 1.14E-280 |
| LRP1 | 7.780344 | 10.809104 | 3.0287597 | 2.05E-280 | 9.45E-279 |
| C6orf141 | 3.4402724 | 1.3671341 | -2.073138 | 5.37E-89 | 9.48E-89 |
| ZCCHC12 | 5.9907573 | 3.2426481 | -2.748109 | 2.76E-195 | 9.35E-195 |
| FOXG1 | 3.870399 | 5.8758095 | 2.0054105 | 1.30E-57 | 1.94E-57 |
| C1QTNF4 | 6.4934768 | 2.96546 | -3.528017 | 1.28E-263 | 1.24E-262 |
| WSCD2 | 5.3238554 | 3.1343454 | -2.18951 | 6.12E-112 | 1.22E-111 |
| BCL11A | 5.4582521 | 3.1311327 | -2.327119 | 3.00E-131 | 6.61E-131 |
| TJP2 | 6.0453072 | 8.0868768 | 2.0415696 | 4.25E-172 | 1.21E-171 |
| SLC7A1 | 5.0703157 | 7.5631782 | 2.4928626 | 8.07E-272 | 1.21E-270 |
| DLGAP5 | 0.1852574 | 2.5931223 | 2.4078648 | 3.60E-245 | 2.17E-244 |
| IFITM2 | 7.7727773 | 5.635957 | -2.13682 | 2.32E-178 | 6.89E-178 |
| FHIT | 5.1208665 | 3.0193496 | -2.101517 | 1.49E-260 | 1.29E-259 |
| EGFL8 | 6.2137126 | 4.2121725 | -2.00154 | 2.83E-251 | 1.93E-250 |
| DDT | 7.6621648 | 5.4380432 | -2.224122 | 5.33E-268 | 6.44E-267 |
| FCGBP | 2.0413595 | 7.1262142 | 5.0848546 | 6.92E-267 | 7.88E-266 |
| LUZP2 | 4.0824008 | 6.4096515 | 2.3272507 | 3.05E-123 | 6.44E-123 |
| TTK | 0.5905608 | 2.7447212 | 2.1541604 | 4.55E-238 | 2.47E-237 |
| PFDN2 | 8.3356345 | 5.9390279 | -2.396607 | 4.60E-276 | 1.04E-274 |
| MEG8 | 4.3360035 | 0.1896464 | -4.146357 | 1.09E-281 | 7.01E-280 |
| ACVR2B | 3.0135798 | 5.2328108 | 2.2192309 | 2.31E-245 | 1.40E-244 |
| ARL17B | 3.6126603 | 0.7386183 | -2.874042 | 1.15E-279 | 4.50E-278 |
| QKI | 7.9764724 | 10.909677 | 2.9332047 | 2.05E-273 | 3.47E-272 |
| KIF21B | 4.8567294 | 7.1951075 | 2.3383782 | 3.44E-173 | 9.84E-173 |
| UBE4A | 5.0271754 | 7.147499 | 2.1203237 | 4.89E-280 | 2.03E-278 |
| BSCL2 | 9.4679647 | 7.1954645 | -2.2725 | 4.60E-238 | 2.49E-237 |
| KCNIP4 | 5.8319781 | 3.8263101 | -2.005668 | 2.16E-181 | 6.57E-181 |
| NACA2 | 0.5734472 | 3.0036224 | 2.4301752 | 1.40E-276 | 3.41E-275 |
| RASSF7 | 5.5293145 | 3.1466907 | -2.382624 | 6.34E-261 | 5.58E-260 |
| SNORA8 | 1.1691263 | 4.7117851 | 3.5426588 | 7.75E-146 | 1.86E-145 |
| PPP1R14A | 7.4394708 | 4.1875124 | -3.251958 | 5.51E-226 | 2.56E-225 |
| FAM120C | 4.2709038 | 6.2933574 | 2.0224537 | 6.25E-284 | 1.23E-281 |
| HLA-H | 3.7552676 | 6.0868104 | 2.3315427 | 1.41E-241 | 8.07E-241 |
| TRMT5 | 3.4635223 | 5.5532046 | 2.0896823 | 1.38E-284 | 4.91E-282 |
| TNPO1 | 4.7331396 | 7.7829563 | 3.0498167 | 4.19E-285 | 3.32E-282 |
| ATP2B4 | 6.4652864 | 8.6377514 | 2.172465 | 2.56E-272 | 4.00E-271 |
| NEFL | 7.2859415 | 5.0988417 | -2.1871 | 6.43E-81 | 1.09E-80 |
| AMIGO3 | 0.7815595 | 3.7218594 | 2.9402999 | 1.25E-266 | 1.41E-265 |
| ANO6 | 3.8885036 | 6.6720777 | 2.7835741 | 1.69E-279 | 6.29E-278 |
| FABP5 | 7.7830675 | 2.5874729 | -5.195595 | 2.43E-283 | 3.25E-281 |
| ALS2CL | 4.6811352 | 2.6698268 | -2.011308 | 3.56E-224 | 1.62E-223 |
| FAM163B | 5.1726296 | 2.4828716 | -2.689758 | 6.48E-171 | 1.83E-170 |
| FAM24B | 3.9401423 | 1.8221893 | -2.117953 | 2.88E-276 | 6.72E-275 |
| UGGT1 | 4.011621 | 6.5882096 | 2.5765885 | 2.04E-284 | 6.23E-282 |
| HSPA5 | 7.5011763 | 9.5253345 | 2.0241582 | 1.93E-253 | 1.38E-252 |
| ASAH2B | 3.1799965 | 1.1187981 | -2.061198 | 6.49E-262 | 5.92E-261 |
| TRRAP | 4.4310965 | 7.1851808 | 2.7540843 | 1.53E-281 | 9.47E-280 |
| ZNF148 | 3.9297122 | 6.3178888 | 2.3881766 | 4.36E-281 | 2.36E-279 |
| HHATL | 8.0036599 | 4.5040959 | -3.499564 | 1.69E-238 | 9.23E-238 |
| PTPRZ1 | 7.5707298 | 11.625038 | 4.0543084 | 1.65E-273 | 2.80E-272 |
| C21orf62 | 1.1461648 | 4.6515003 | 3.5053354 | 3.84E-249 | 2.50E-248 |
| DEFA1B | 2.7204575 | 0.5928512 | -2.127606 | 2.04E-118 | 4.18E-118 |
| BEGAIN | 6.3937786 | 3.6090631 | -2.784715 | 1.29E-240 | 7.27E-240 |
| S1PR2 | 2.0070707 | 4.1819406 | 2.1748699 | 6.08E-276 | 1.34E-274 |
| DOC2A | 6.6873248 | 3.6538759 | -3.033449 | 3.19E-198 | 1.11E-197 |
| PRKDC | 6.261952 | 8.2743761 | 2.0124241 | 2.03E-280 | 9.42E-279 |
| SOX2 | 6.7583843 | 9.5142451 | 2.7558608 | 4.02E-259 | 3.30E-258 |
| UNC13C | 5.4778285 | 2.7790122 | -2.698816 | 2.44E-157 | 6.27E-157 |
| CHD6 | 4.7831204 | 6.8717099 | 2.0885895 | 1.82E-264 | 1.85E-263 |
| RBP7 | 4.5519994 | 2.4715901 | -2.080409 | 1.71E-180 | 5.16E-180 |
| GRHL3 | 1.1628736 | 3.328174 | 2.1653004 | 8.12E-201 | 2.90E-200 |
| NPPC | 3.0893929 | 0.4536485 | -2.635744 | 1.73E-262 | 1.62E-261 |
| TOP2A | 0.7680121 | 5.4812461 | 4.713234 | 5.34E-268 | 6.44E-267 |
| HAPLN2 | 7.8449698 | 4.4407472 | -3.404223 | 2.81E-229 | 1.36E-228 |
| RPPH1 | 5.3838811 | 2.3969653 | -2.986916 | 7.45E-231 | 3.66E-230 |
| SDC3 | 7.4050523 | 10.516118 | 3.111066 | 6.19E-278 | 1.84E-276 |
| SLC25A27 | 7.1099829 | 4.7409261 | -2.369057 | 5.43E-274 | 9.63E-273 |
| NCAPH | 1.3545246 | 3.3890213 | 2.0344967 | 6.51E-229 | 3.13E-228 |
| EMP1 | 5.0523425 | 7.2293156 | 2.1769732 | 2.66E-125 | 5.67E-125 |
| PPIA | 11.46064 | 8.4519074 | -3.008732 | 3.11E-275 | 6.28E-274 |
| SORL1 | 6.0681621 | 8.9551132 | 2.8869511 | 5.72E-271 | 8.20E-270 |
| SOCS6 | 3.9477624 | 6.048652 | 2.1008897 | 6.41E-275 | 1.25E-273 |
| DEPDC1 | 0.3117565 | 2.4317437 | 2.1199872 | 1.34E-264 | 1.36E-263 |
| COX7C | 10.485369 | 7.5870416 | -2.898327 | 1.01E-277 | 2.89E-276 |
| HRH1 | 2.4802154 | 4.9758288 | 2.4956134 | 1.06E-218 | 4.54E-218 |
| ERAP2 | 2.5962189 | 4.6500885 | 2.0538696 | 2.22E-160 | 5.81E-160 |
| NME2P1 | 0.6036765 | 2.6238351 | 2.0201587 | 5.84E-266 | 6.34E-265 |
| MATR3 | 7.340501 | 9.7597316 | 2.4192306 | 2.06E-282 | 1.72E-280 |
| PDXP | 8.3927044 | 6.3005321 | -2.092172 | 2.15E-223 | 9.69E-223 |
| ZACN | 6.5437521 | 0.5101509 | -6.033601 | 7.54E-282 | 5.20E-280 |
| RGS11 | 7.7144528 | 5.0707259 | -2.643727 | 7.30E-247 | 4.55E-246 |
| WDR3 | 3.2092467 | 5.7184071 | 2.5091604 | 6.16E-285 | 3.32E-282 |
| C3 | 7.2548082 | 9.5138904 | 2.2590823 | 1.29E-130 | 2.83E-130 |
| RAB3A | 8.4838345 | 5.3752822 | -3.108552 | 1.26E-212 | 5.04E-212 |
| TMEM160 | 6.77059 | 3.4157272 | -3.354863 | 1.14E-276 | 2.78E-275 |
| UCHL1 | 10.690961 | 8.2513752 | -2.439586 | 5.20E-210 | 2.02E-209 |
| CPD | 4.5391286 | 6.6879507 | 2.1488221 | 2.41E-269 | 3.11E-268 |
| ACTA1 | 4.2895401 | 1.2439089 | -3.045631 | 1.88E-281 | 1.12E-279 |
| RNF181 | 8.0357353 | 5.9481096 | -2.087626 | 1.66E-266 | 1.86E-265 |
| SIK1 | 2.6538886 | 4.8939531 | 2.2400646 | 1.48E-188 | 4.77E-188 |
| CAMK1G | 5.2086317 | 2.9236575 | -2.284974 | 1.06E-113 | 2.11E-113 |
| PNMT | 5.3228788 | 1.9385827 | -3.384296 | 2.34E-253 | 1.67E-252 |
| EPCAM | 3.7648562 | 1.6933121 | -2.071544 | 5.83E-214 | 2.37E-213 |
| BBX | 4.7708692 | 7.0665621 | 2.2956929 | 8.13E-276 | 1.76E-274 |
| KCNIP2 | 7.4930299 | 5.2719622 | -2.221068 | 2.98E-129 | 6.49E-129 |
| KLHDC9 | 6.4394092 | 3.6247328 | -2.814676 | 1.86E-277 | 5.10E-276 |
| COX5B | 9.5946269 | 7.0905861 | -2.504041 | 9.02E-274 | 1.57E-272 |
| KCNQ1OT1 | 0.7601776 | 2.8240415 | 2.0638639 | 8.32E-264 | 8.18E-263 |
| CENPF | 1.513739 | 4.9921841 | 3.478445 | 1.91E-264 | 1.93E-263 |
| NFIX | 7.6562072 | 9.9596994 | 2.3034922 | 4.19E-261 | 3.71E-260 |
| LPCAT4 | 8.1315437 | 4.9618535 | -3.16969 | 2.93E-266 | 3.23E-265 |
| CKM | 3.3673465 | 0.5839279 | -2.783419 | 1.21E-270 | 1.69E-269 |
| KIAA1211 | 3.1364024 | 6.0029268 | 2.8665244 | 4.36E-275 | 8.60E-274 |
| ANXA2P2 | 1.1938878 | 5.2203281 | 4.0264403 | 7.89E-274 | 1.38E-272 |
| CORO6 | 6.8465769 | 2.9537368 | -3.89284 | 2.87E-264 | 2.87E-263 |
| PLXNA4 | 3.571597 | 6.1661548 | 2.5945578 | 7.12E-246 | 4.35E-245 |
| PDLIM5 | 5.4280224 | 7.9997352 | 2.5717128 | 3.69E-262 | 3.41E-261 |
| C8orf59 | 6.9599987 | 4.849315 | -2.110684 | 1.13E-269 | 1.48E-268 |
| FABP3 | 7.4041537 | 4.044613 | -3.359541 | 2.07E-265 | 2.19E-264 |
| DOCK2 | 2.7296408 | 4.7733197 | 2.0436789 | 1.81E-197 | 6.26E-197 |
| PARP14 | 3.9781577 | 6.1805398 | 2.2023822 | 1.82E-252 | 1.27E-251 |
| AMY2A | 3.1728742 | 0.7108871 | -2.461987 | 9.94E-237 | 5.30E-236 |
| EZH2 | 2.5000946 | 4.5469046 | 2.0468101 | 2.08E-193 | 6.95E-193 |
| SCN1B | 8.1936301 | 5.005076 | -3.188554 | 4.19E-252 | 2.90E-251 |
| TPX2 | 1.926073 | 4.9328198 | 3.0067468 | 9.80E-269 | 1.23E-267 |
| LRP5 | 3.9174341 | 6.0836675 | 2.1662334 | 3.25E-261 | 2.88E-260 |
| BMPR1A | 3.5020651 | 5.5873534 | 2.0852884 | 5.54E-282 | 3.99E-280 |
| NKX6-2 | 6.2179808 | 3.6952899 | -2.522691 | 1.14E-158 | 2.95E-158 |
| MYC | 3.07395 | 6.1787684 | 3.1048184 | 3.69E-265 | 3.85E-264 |
| SFI1 | 6.6513457 | 4.5157573 | -2.135588 | 5.28E-266 | 5.75E-265 |
| FAM173A | 6.8681015 | 3.8978531 | -2.970248 | 2.95E-277 | 7.90E-276 |
| DOCK8 | 2.5179176 | 5.0246924 | 2.5067747 | 6.06E-232 | 3.03E-231 |
| COX7A1 | 7.069661 | 3.4242436 | -3.645417 | 3.89E-274 | 7.03E-273 |
| NFAM1 | 1.5830445 | 4.0141284 | 2.431084 | 1.98E-264 | 2.00E-263 |
| CDCA2 | 0.3513328 | 2.3584614 | 2.0071286 | 3.80E-262 | 3.50E-261 |
| DNER | 7.401865 | 9.4564201 | 2.0545551 | 4.80E-218 | 2.03E-217 |
| PGAP1 | 4.1160531 | 6.3809837 | 2.2649306 | 5.98E-272 | 9.06E-271 |
| CLEC4G | 3.3241946 | 0.931703 | -2.392492 | 4.63E-215 | 1.90E-214 |
| GSTM5 | 6.6230751 | 3.6792313 | -2.943844 | 2.35E-209 | 9.10E-209 |
| IMMP1L | 6.8245574 | 3.0511748 | -3.773383 | 2.43E-280 | 1.11E-278 |
| TTR | 3.8168927 | 0.3196348 | -3.497258 | 3.99E-227 | 1.87E-226 |
| FEM1A | 2.6857501 | 6.3707141 | 3.6849639 | 1.01E-282 | 9.65E-281 |
| COX7A2 | 9.5350477 | 7.0149435 | -2.520104 | 1.33E-272 | 2.13E-271 |
| KRT13 | 2.695392 | 0.2694169 | -2.425975 | 8.30E-146 | 1.99E-145 |
| DLG5 | 5.1012873 | 7.6510172 | 2.5497299 | 4.41E-277 | 1.14E-275 |
| F5 | 1.6190269 | 3.8517615 | 2.2327346 | 1.04E-119 | 2.16E-119 |
| KIF25 | 3.129324 | 0.7257404 | -2.403584 | 1.74E-254 | 1.28E-253 |
| TBC1D3 | 0.9646138 | 4.5665686 | 3.6019548 | 2.65E-174 | 7.64E-174 |
| ANKIB1 | 5.5325706 | 7.673802 | 2.1412314 | 1.10E-275 | 2.33E-274 |
| CKMT1A | 6.3668488 | 3.1980445 | -3.168804 | 6.12E-211 | 2.40E-210 |
| TMEM33 | 4.6036651 | 6.7794721 | 2.175807 | 1.33E-283 | 2.11E-281 |
| WDR5B | 2.5700587 | 4.8332571 | 2.2631984 | 3.08E-284 | 7.96E-282 |
| SNAP25 | 10.844798 | 7.5148823 | -3.329915 | 3.08E-152 | 7.66E-152 |
| ACLY | 6.1513229 | 8.2545084 | 2.1031855 | 2.78E-281 | 1.59E-279 |
| MLEC | 6.3842116 | 8.3999532 | 2.0157416 | 5.13E-283 | 5.47E-281 |
| ZDHHC11 | 5.5986647 | 3.0748518 | -2.523813 | 2.33E-256 | 1.78E-255 |
| CANX | 8.4183978 | 10.775687 | 2.3572889 | 4.29E-284 | 9.84E-282 |
| FNDC3B | 3.0949298 | 5.1522166 | 2.0572868 | 1.12E-264 | 1.15E-263 |
| TGFBR1 | 3.9917392 | 6.5329058 | 2.5411666 | 4.87E-276 | 1.10E-274 |
| MS4A7 | 3.0788585 | 5.1989887 | 2.1201302 | 6.51E-169 | 1.81E-168 |
| ABCA10 | 3.4297582 | 1.0986779 | -2.33108 | 1.94E-272 | 3.08E-271 |
| ABCA5 | 5.8869179 | 3.5495519 | -2.337366 | 3.69E-236 | 1.95E-235 |
| PARP4 | 3.8563848 | 6.1788276 | 2.3224428 | 2.83E-276 | 6.60E-275 |
| MMP2 | 3.2449964 | 6.4439892 | 3.1989928 | 3.96E-272 | 6.08E-271 |
| SLCO4A1 | 5.7521112 | 3.4291883 | -2.322923 | 2.88E-236 | 1.53E-235 |
| ZNF681 | 2.0056695 | 4.0302511 | 2.0245816 | 2.48E-239 | 1.37E-238 |
| EP300 | 4.9040258 | 7.1195198 | 2.215494 | 4.95E-265 | 5.14E-264 |
| CABP1 | 7.5469592 | 3.7455152 | -3.801444 | 7.79E-228 | 3.69E-227 |
| NDC80 | 0.3966949 | 3.0665714 | 2.6698765 | 3.17E-260 | 2.69E-259 |
| SEC61A1 | 6.8448526 | 9.1313825 | 2.2865298 | 3.77E-283 | 4.46E-281 |
| IFI27L1 | 6.1684484 | 3.9975158 | -2.170933 | 1.64E-273 | 2.79E-272 |
| VSTM2B | 6.4335569 | 3.5386545 | -2.894902 | 1.09E-273 | 1.88E-272 |
| RNF207 | 4.8130257 | 2.5204138 | -2.292612 | 2.52E-222 | 1.12E-221 |
| RP2 | 2.8868217 | 5.1407203 | 2.2538986 | 3.29E-278 | 1.02E-276 |
| SOHLH1 | 4.0318552 | 1.4491723 | -2.582683 | 1.58E-154 | 3.98E-154 |
| TANC2 | 4.9233509 | 7.2097286 | 2.2863777 | 5.24E-266 | 5.71E-265 |
| CAMK4 | 4.8325956 | 2.5905325 | -2.242063 | 5.59E-156 | 1.42E-155 |
| NSUN5P1 | 6.4282351 | 3.8578693 | -2.570366 | 5.53E-274 | 9.80E-273 |
| TNNT2 | 3.6193657 | 1.0884621 | -2.530904 | 4.17E-150 | 1.02E-149 |
| RHOXF1 | 2.965799 | 0.7708842 | -2.194915 | 3.83E-172 | 1.09E-171 |
| FBXL16 | 9.2496764 | 7.0937828 | -2.155894 | 2.96E-130 | 6.50E-130 |
| SFRP2 | 3.1906046 | 5.4030304 | 2.2124258 | 2.94E-69 | 4.66E-69 |
| ABCC8 | 7.1731611 | 4.5907602 | -2.582401 | 2.64E-177 | 7.79E-177 |
| TCF12 | 5.7481127 | 9.2922531 | 3.5441404 | 2.13E-281 | 1.25E-279 |
| SRRM3 | 7.4456965 | 4.6596358 | -2.786061 | 1.20E-188 | 3.85E-188 |
| SMC1A | 4.8224976 | 7.3079287 | 2.4854311 | 7.33E-283 | 7.46E-281 |
| PCSK1N | 9.5866335 | 6.4201056 | -3.166528 | 5.72E-255 | 4.23E-254 |
| EIF5AL1 | 1.0305882 | 6.3115994 | 5.2810111 | 2.77E-285 | 3.32E-282 |
| ZFR2 | 5.1428356 | 2.7630968 | -2.379739 | 3.39E-190 | 1.10E-189 |
| 11-Mar | 3.6265887 | 1.1371872 | -2.489401 | 1.76E-219 | 7.58E-219 |
| UQCRH | 9.635635 | 6.7150444 | -2.920591 | 4.54E-278 | 1.38E-276 |
| FAAH | 6.7411346 | 4.5013361 | -2.239798 | 6.74E-259 | 5.49E-258 |
| MAL | 7.7156316 | 4.6657555 | -3.049876 | 5.91E-192 | 1.95E-191 |
| HDAC4 | 4.3581962 | 6.4756818 | 2.1174856 | 3.04E-269 | 3.91E-268 |
| PLCH2 | 6.3651261 | 4.1706462 | -2.19448 | 2.93E-177 | 8.65E-177 |
| NDUFA11 | 9.1456672 | 7.092657 | -2.05301 | 8.53E-262 | 7.75E-261 |
| ZHX3 | 5.9305777 | 7.9864274 | 2.0558497 | 2.11E-271 | 3.10E-270 |
| NDUFC1 | 7.9164898 | 5.6120801 | -2.30441 | 6.88E-276 | 1.51E-274 |
| C1orf194 | 4.9989348 | 2.6263825 | -2.372552 | 7.66E-165 | 2.07E-164 |
| TET3 | 3.2340643 | 5.6489979 | 2.4149336 | 3.06E-272 | 4.74E-271 |
| AQP4 | 7.9313944 | 10.787337 | 2.8559428 | 8.19E-192 | 2.70E-191 |
| HPCA | 8.3183138 | 4.4492349 | -3.869079 | 2.49E-195 | 8.43E-195 |
| TXNDC5 | 4.8660892 | 6.8862167 | 2.0201275 | 5.53E-276 | 1.24E-274 |
| TIMP2 | 7.6317685 | 10.246966 | 2.6151974 | 8.15E-278 | 2.37E-276 |
| GAS5 | 8.6919288 | 6.213978 | -2.477951 | 1.04E-263 | 1.02E-262 |
| RNF175 | 5.4625684 | 2.9725967 | -2.489972 | 1.40E-242 | 8.10E-242 |
| RPS21 | 11.329848 | 8.4717736 | -2.858075 | 3.62E-274 | 6.57E-273 |
| FAT3 | 3.7444831 | 6.1915478 | 2.4470647 | 1.34E-215 | 5.52E-215 |
| AHNAK | 6.0826174 | 9.4177291 | 3.3351117 | 5.49E-253 | 3.89E-252 |
| PALM2-AKAP2 | 1.4865565 | 5.0100015 | 3.523445 | 6.11E-279 | 2.10E-277 |
| RPL37A | 12.450948 | 10.049978 | -2.400969 | 8.49E-273 | 1.38E-271 |
| TP53TG5 | 3.8463819 | 0.7987621 | -3.04762 | 5.70E-271 | 8.18E-270 |
| PGAM4 | 0.5098297 | 3.2017688 | 2.6919392 | 4.87E-276 | 1.10E-274 |
| DHRS11 | 6.006932 | 3.9199304 | -2.087002 | 7.99E-269 | 1.00E-267 |
| PYROXD2 | 4.6398957 | 2.4463336 | -2.193562 | 2.34E-268 | 2.89E-267 |
| PENK | 5.7402403 | 2.5082501 | -3.23199 | 1.65E-146 | 3.95E-146 |
| DNAJC12 | 6.6421129 | 4.5329061 | -2.109207 | 8.46E-266 | 9.07E-265 |
| FHDC1 | 2.2535275 | 4.3299217 | 2.0763942 | 5.76E-187 | 1.83E-186 |
| TSPAN19 | 2.5790853 | 0.3055427 | -2.273543 | 1.33E-276 | 3.24E-275 |
| FAM84B | 4.5436785 | 7.0366842 | 2.4930057 | 1.90E-227 | 8.98E-227 |
| BCHE | 4.5645834 | 6.7556321 | 2.1910488 | 1.42E-228 | 6.79E-228 |
| GNL3L | 2.5255403 | 5.174541 | 2.6490008 | 7.91E-283 | 7.94E-281 |
| 2-Sep | 8.5727211 | 10.640584 | 2.0678629 | 7.90E-276 | 1.71E-274 |
| WEE1 | 2.8534464 | 4.8735178 | 2.0200715 | 7.03E-201 | 2.51E-200 |
| SH3BP4 | 4.4739238 | 6.5453768 | 2.071453 | 3.01E-255 | 2.24E-254 |
| SLC25A41 | 3.9986599 | 1.9596055 | -2.039054 | 1.52E-221 | 6.70E-221 |
| SP1 | 4.6328437 | 6.648853 | 2.0160094 | 3.05E-275 | 6.17E-274 |
| DYNLRB1 | 9.6801015 | 7.5679765 | -2.112125 | 3.99E-277 | 1.04E-275 |
| CCDC84 | 6.7153789 | 3.7398005 | -2.975578 | 7.03E-280 | 2.85E-278 |
| STAG2 | 5.5912505 | 7.7439923 | 2.1527418 | 2.62E-279 | 9.48E-278 |
| SNHG8 | 8.3975573 | 5.0393053 | -3.358252 | 1.82E-276 | 4.33E-275 |
| CD180 | 0.7674778 | 3.0870231 | 2.3195453 | 2.15E-261 | 1.92E-260 |
| RPE65 | 1.0990944 | 3.8607159 | 2.7616215 | 9.47E-213 | 3.80E-212 |
| DGKZ | 8.4340853 | 6.241965 | -2.19212 | 3.91E-226 | 1.82E-225 |
| KCNK9 | 4.0595309 | 1.9991854 | -2.060346 | 2.35E-82 | 4.01E-82 |
| HDDC2 | 8.5312059 | 6.4205351 | -2.110671 | 3.78E-273 | 6.29E-272 |
| KRT14 | 2.5258511 | 0.4027388 | -2.123112 | 6.22E-212 | 2.47E-211 |
| ERAP1 | 4.0059333 | 6.1309278 | 2.1249944 | 2.67E-279 | 9.66E-278 |
| RHOBTB3 | 6.3495749 | 8.5509246 | 2.2013497 | 2.57E-258 | 2.07E-257 |
| S100A13 | 8.9844966 | 5.7078901 | -3.276607 | 1.17E-252 | 8.21E-252 |
| KCTD12 | 5.6629357 | 7.8803892 | 2.2174535 | 1.01E-243 | 5.94E-243 |
| TESC | 5.9425065 | 3.4007271 | -2.541779 | 3.64E-209 | 1.41E-208 |
| HSBP1L1 | 6.130366 | 2.4407568 | -3.689609 | 5.11E-284 | 1.06E-281 |
| DCHS1 | 3.7602405 | 6.5961605 | 2.83592 | 1.15E-278 | 3.72E-277 |
| AIFM3 | 7.5839245 | 3.8344978 | -3.749427 | 3.02E-259 | 2.49E-258 |
| B4GALT5 | 5.3786364 | 7.4055905 | 2.0269542 | 2.64E-280 | 1.19E-278 |
| KCNT1 | 6.0157309 | 2.9551594 | -3.060572 | 3.86E-178 | 1.14E-177 |
| RAB40B | 7.5735111 | 5.2880705 | -2.285441 | 1.55E-255 | 1.16E-254 |
| NDUFA12 | 8.1874346 | 5.8624675 | -2.324967 | 1.91E-276 | 4.51E-275 |
| PLIN5 | 4.4401889 | 2.3368898 | -2.103299 | 8.97E-221 | 3.93E-220 |
| DYNLRB2 | 4.605112 | 1.7235529 | -2.881559 | 1.72E-269 | 2.23E-268 |
| ITPR2 | 3.7228772 | 7.0264177 | 3.3035405 | 3.72E-282 | 2.79E-280 |
| CENPV | 7.1869357 | 3.7484887 | -3.438447 | 1.91E-266 | 2.13E-265 |
| DTNB | 7.0646319 | 4.737252 | -2.32738 | 4.13E-275 | 8.19E-274 |
| MDFI | 3.5681241 | 5.841926 | 2.273802 | 1.27E-172 | 3.62E-172 |
| GTSE1 | 0.8930045 | 3.2774974 | 2.3844929 | 5.05E-254 | 3.66E-253 |
| FGF22 | 3.7635159 | 0.4442114 | -3.319305 | 1.53E-283 | 2.32E-281 |
| SYNPR | 7.0869963 | 3.3876153 | -3.699381 | 8.77E-210 | 3.40E-209 |
| IMPAD1 | 5.4803344 | 7.7175204 | 2.237186 | 4.04E-283 | 4.63E-281 |
| RGS8 | 4.0080969 | 0.856012 | -3.152085 | 4.73E-257 | 3.67E-256 |
| HEATR5A | 4.292465 | 6.3889436 | 2.0964786 | 2.24E-274 | 4.14E-273 |
| MB | 3.7143811 | 1.7063606 | -2.008021 | 1.76E-219 | 7.58E-219 |
| LRP6 | 4.2215509 | 6.2298883 | 2.0083374 | 1.23E-275 | 2.59E-274 |
| XPR1 | 4.175144 | 6.7959545 | 2.6208105 | 1.62E-284 | 5.31E-282 |
| DPM3 | 7.4323492 | 4.8927483 | -2.539601 | 3.64E-265 | 3.80E-264 |
| JPH2 | 1.3399933 | 3.4227443 | 2.0827511 | 1.88E-241 | 1.07E-240 |
| GATC | 4.9498963 | 2.4548804 | -2.495016 | 3.65E-284 | 8.78E-282 |
| ARID5B | 3.8323843 | 6.3567324 | 2.5243481 | 3.34E-277 | 8.87E-276 |
| NFIB | 5.2113895 | 8.1428734 | 2.9314839 | 1.12E-281 | 7.22E-280 |
| NRG4 | 3.7790196 | 0.6941843 | -3.084835 | 1.83E-279 | 6.79E-278 |
| DES | 4.3925777 | 2.0276022 | -2.364976 | 5.04E-162 | 1.33E-161 |
| PWP2 | 3.6897948 | 5.9914531 | 2.3016583 | 9.73E-275 | 1.86E-273 |
| LPL | 5.4160395 | 7.934913 | 2.5188736 | 4.51E-168 | 1.25E-167 |
| SYT11 | 8.1755935 | 10.612724 | 2.4371302 | 6.07E-268 | 7.29E-267 |
| TTTY14 | 3.4413628 | 0.6970665 | -2.744296 | 1.41E-78 | 2.36E-78 |
| GPR27 | 5.9028739 | 1.9632699 | -3.939604 | 1.70E-283 | 2.49E-281 |
| RBP4 | 5.722602 | 2.3539429 | -3.368659 | 4.48E-149 | 1.09E-148 |
| GABRA2 | 5.5202664 | 2.9627034 | -2.557563 | 4.44E-184 | 1.37E-183 |
| PCP4 | 8.6320908 | 2.9968113 | -5.63528 | 1.02E-278 | 3.34E-277 |
| ACTL6B | 6.2712325 | 4.2567894 | -2.014443 | 4.07E-127 | 8.77E-127 |
| SLC6A13 | 4.7175238 | 2.3758858 | -2.341638 | 3.55E-252 | 2.47E-251 |
| MAVS | 4.8701619 | 7.7236445 | 2.8534826 | 2.32E-285 | 3.32E-282 |
| GPR89B | 4.9832287 | 1.9383109 | -3.044918 | 8.01E-282 | 5.49E-280 |
| TCEAL5 | 7.4797783 | 5.1105222 | -2.369256 | 2.69E-265 | 2.83E-264 |
| GALNT4 | 1.6388141 | 3.8037512 | 2.1649371 | 1.82E-275 | 3.81E-274 |
| EIF3C | 9.2193798 | 4.1498255 | -5.069554 | 1.59E-282 | 1.38E-280 |
| CYGB | 5.3121666 | 3.1546341 | -2.157533 | 1.04E-217 | 4.37E-217 |
| ANKRD37 | 6.1252275 | 3.2831044 | -2.842123 | 3.23E-284 | 8.13E-282 |
| EEA1 | 3.5138552 | 5.9431536 | 2.4292985 | 2.12E-280 | 9.76E-279 |
| MOBP | 8.6887151 | 6.1247117 | -2.564003 | 2.60E-91 | 4.64E-91 |
| CLIC4 | 6.3929408 | 9.1488111 | 2.7558703 | 8.29E-252 | 5.70E-251 |
| SCARNA9 | 3.8004273 | 1.2319362 | -2.568491 | 2.00E-256 | 1.53E-255 |
| UCN | 4.4033278 | 2.0070616 | -2.396266 | 3.57E-270 | 4.80E-269 |
| GNG3 | 9.1507128 | 4.5283224 | -4.62239 | 2.82E-262 | 2.61E-261 |
| FAM111B | 0.2352804 | 2.8557766 | 2.6204962 | 2.84E-268 | 3.49E-267 |
| MAGT1 | 4.7645522 | 6.846004 | 2.0814518 | 2.93E-257 | 2.29E-256 |
| RPL21 | 10.609551 | 1.5003678 | -9.109184 | 1.08E-281 | 7.01E-280 |
| ZKSCAN2 | 2.6586422 | 4.7814023 | 2.1227601 | 3.17E-281 | 1.79E-279 |
| MMP14 | 3.7743017 | 6.7182364 | 2.9439347 | 8.10E-245 | 4.86E-244 |
| KDM5A | 3.6845538 | 6.1505301 | 2.4659762 | 6.01E-280 | 2.45E-278 |
| CHI3L2 | 3.0934052 | 5.3986597 | 2.3052545 | 1.08E-86 | 1.88E-86 |
| HMBOX1 | 6.1145127 | 3.3680017 | -2.746511 | 7.89E-281 | 3.97E-279 |
| RRM2 | 0.672233 | 4.2186538 | 3.5464209 | 9.53E-257 | 7.35E-256 |
| EIF4EBP2 | 5.8077486 | 7.9803646 | 2.172616 | 6.78E-285 | 3.38E-282 |
| CCDC159 | 6.9938581 | 4.3411369 | -2.652721 | 3.33E-278 | 1.02E-276 |
| CRABP1 | 5.7609272 | 1.7395334 | -4.021394 | 1.86E-245 | 1.13E-244 |
| NEDD4 | 2.277907 | 4.5740908 | 2.2961838 | 3.40E-277 | 8.99E-276 |
| VSTM2L | 6.4543877 | 4.3038626 | -2.150525 | 1.17E-149 | 2.87E-149 |
| MYL2 | 3.4282269 | 0.0361518 | -3.392075 | 2.23E-284 | 6.33E-282 |
| CD109 | 1.9090749 | 4.089309 | 2.1802341 | 3.25E-259 | 2.67E-258 |
| GLS2 | 5.8672818 | 2.5548865 | -3.312395 | 4.15E-237 | 2.22E-236 |
| CHST3 | 4.031279 | 6.7132443 | 2.6819653 | 2.51E-268 | 3.08E-267 |
| FERMT1 | 2.6598556 | 5.8576548 | 3.1977992 | 6.28E-205 | 2.32E-204 |
| ECHDC2 | 7.9558858 | 3.9990278 | -3.956858 | 1.36E-278 | 4.32E-277 |
| EGR2 | 2.1357553 | 4.552043 | 2.4162877 | 2.60E-178 | 7.74E-178 |
| AGPAT5 | 5.662894 | 7.6790598 | 2.0161658 | 2.78E-279 | 1.00E-277 |
| TCAP | 4.6339644 | 2.6328694 | -2.001095 | 1.26E-257 | 9.99E-257 |
| GRIN1 | 8.4055789 | 5.2326455 | -3.172933 | 1.32E-158 | 3.42E-158 |
| ST8SIA3 | 7.5507587 | 5.1918175 | -2.358941 | 1.23E-137 | 2.82E-137 |
| HNRNPA1L2 | 4.2034501 | 7.2661194 | 3.0626694 | 2.43E-285 | 3.32E-282 |
| NAV1 | 5.0151557 | 8.1246931 | 3.1095374 | 3.47E-277 | 9.11E-276 |
| UBL5 | 9.9230592 | 7.3350158 | -2.588043 | 5.58E-274 | 9.86E-273 |
| RPS28 | 10.259487 | 3.4759214 | -6.783566 | 1.35E-279 | 5.19E-278 |
| RPS15 | 11.125899 | 8.2395276 | -2.886372 | 8.70E-274 | 1.51E-272 |
| TUBA8 | 6.1024195 | 3.510827 | -2.591593 | 9.92E-211 | 3.89E-210 |
| ARPP21 | 7.9289144 | 5.2201486 | -2.708766 | 1.65E-167 | 4.54E-167 |
| MAP1LC3A | 8.9342462 | 5.3417946 | -3.592452 | 1.25E-278 | 4.00E-277 |
| ZDHHC8P1 | 5.2855388 | 2.4958448 | -2.789694 | 9.82E-254 | 7.08E-253 |
| MAML2 | 3.4317054 | 6.1114368 | 2.6797314 | 4.07E-265 | 4.24E-264 |
| HSF4 | 7.578653 | 3.6720869 | -3.906566 | 2.45E-280 | 1.11E-278 |
| CCR1 | 2.0235381 | 4.2251199 | 2.2015818 | 2.06E-194 | 6.93E-194 |
| AFAP1 | 4.2740348 | 6.9411679 | 2.667133 | 2.44E-282 | 1.97E-280 |
| SPATA5 | 1.700456 | 3.8488251 | 2.1483691 | 1.91E-275 | 3.97E-274 |
| PLCXD2 | 1.5966318 | 3.6444684 | 2.0478367 | 1.82E-188 | 5.85E-188 |
| MED13L | 4.7697727 | 7.2479041 | 2.4781315 | 6.40E-259 | 5.22E-258 |
| PRKD3 | 4.1860471 | 6.3350421 | 2.148995 | 2.06E-282 | 1.72E-280 |
| GUK1 | 10.096796 | 8.0466204 | -2.050176 | 2.10E-267 | 2.45E-266 |
| CKMT1B | 7.4115191 | 4.3051537 | -3.106365 | 1.37E-202 | 4.98E-202 |
| NMB | 4.546139 | 6.7977898 | 2.2516507 | 2.11E-161 | 5.55E-161 |
| CCND2 | 5.1933852 | 8.5701508 | 3.3767656 | 9.88E-278 | 2.84E-276 |
| PSMB10 | 7.5881552 | 5.4812339 | -2.106921 | 6.65E-239 | 3.65E-238 |
| MAFB | 3.3828091 | 6.3220597 | 2.9392506 | 1.12E-263 | 1.10E-262 |
| GSTO2 | 4.3457823 | 2.1982464 | -2.147536 | 1.13E-252 | 7.90E-252 |
| NAA38 | 8.2142334 | 4.6213191 | -3.592914 | 2.60E-274 | 4.79E-273 |
| BEX2 | 8.8609851 | 5.852672 | -3.008313 | 7.81E-277 | 1.95E-275 |
| SOX4 | 3.4205791 | 8.1069251 | 4.686346 | 2.39E-282 | 1.94E-280 |
| OSGEP | 7.1183243 | 5.1172881 | -2.001036 | 1.87E-273 | 3.18E-272 |
| TSTD1 | 6.2888098 | 2.7289852 | -3.559825 | 3.89E-252 | 2.71E-251 |
| QSER1 | 3.6896559 | 6.4218278 | 2.7321719 | 6.76E-282 | 4.74E-280 |
| EME2 | 5.4561516 | 1.9325448 | -3.523607 | 5.13E-284 | 1.06E-281 |
| PRCD | 5.773467 | 2.5069502 | -3.266517 | 6.46E-277 | 1.64E-275 |
| EEF1A2 | 9.5033581 | 7.0989699 | -2.404388 | 2.09E-166 | 5.71E-166 |
| SCARA3 | 6.4782855 | 8.4879433 | 2.0096579 | 2.35E-212 | 9.36E-212 |
| IDI2 | 0.2461261 | 2.8748327 | 2.6287065 | 2.84E-274 | 5.20E-273 |
| NDUFB8 | 9.7621613 | 6.7924649 | -2.969696 | 2.16E-279 | 7.89E-278 |
| MTCP1 | 5.8488996 | 1.9741301 | -3.874769 | 7.34E-281 | 3.74E-279 |
| RAB26 | 7.4429043 | 3.4107414 | -4.032163 | 1.13E-267 | 1.34E-266 |
| HNRNPA3P1 | 0.0508419 | 2.7256496 | 2.6748077 | 6.26E-280 | 2.55E-278 |
| ASPM | 0.3709611 | 3.2558556 | 2.8848945 | 1.07E-274 | 2.04E-273 |
| SSR1 | 5.8223459 | 7.9131527 | 2.0908068 | 4.56E-285 | 3.32E-282 |
| CASK | 4.6073984 | 7.4378931 | 2.8304947 | 6.47E-285 | 3.34E-282 |
| STX1A | 7.0917682 | 4.8786529 | -2.213115 | 6.55E-135 | 1.48E-134 |
| EDNRB | 5.8523272 | 8.7039377 | 2.8516105 | 8.55E-200 | 3.02E-199 |
| STMN2 | 8.8244124 | 6.0003037 | -2.824109 | 6.01E-140 | 1.40E-139 |
| FAM129A | 2.0617356 | 4.5944325 | 2.5326969 | 2.22E-250 | 1.48E-249 |
| S100A9 | 6.1061352 | 3.5304968 | -2.575638 | 4.50E-153 | 1.12E-152 |
| ANKRD50 | 2.9546747 | 5.3381734 | 2.3834987 | 2.88E-281 | 1.64E-279 |
| DNM1 | 8.8770301 | 6.6396423 | -2.237388 | 1.60E-122 | 3.36E-122 |
| SLC25A28 | 7.7077292 | 5.65396 | -2.053769 | 2.91E-276 | 6.78E-275 |
| EGFL7 | 7.0681476 | 4.911549 | -2.156599 | 1.27E-210 | 4.96E-210 |
| TCEA2 | 8.5355315 | 6.4777539 | -2.057778 | 2.54E-275 | 5.19E-274 |
| CALB1 | 5.5804295 | 2.8906372 | -2.689792 | 2.49E-154 | 6.27E-154 |
| SAMD9L | 3.0295254 | 5.0852602 | 2.0557349 | 1.68E-227 | 7.94E-227 |
| NDUFB3 | 7.7434681 | 5.671399 | -2.072069 | 2.56E-272 | 4.00E-271 |
| DNAJC4 | 7.22524 | 5.0760003 | -2.14924 | 3.26E-271 | 4.74E-270 |
| LRRC37A2 | 5.6614013 | 3.6111307 | -2.050271 | 7.97E-253 | 5.62E-252 |
| SNRNP200 | 7.295055 | 9.414565 | 2.11951 | 4.17E-275 | 8.26E-274 |
| KIF4A | 0.9030904 | 3.667723 | 2.7646326 | 1.19E-273 | 2.05E-272 |
| SYTL1 | 3.7894617 | 1.3952028 | -2.394259 | 7.02E-207 | 2.64E-206 |
| MICALL2 | 7.131068 | 4.9914429 | -2.139625 | 3.32E-229 | 1.60E-228 |
| IGF2BP3 | 0.454292 | 2.855456 | 2.401164 | 6.79E-257 | 5.26E-256 |
| GABRA6 | 2.7554691 | 0.224801 | -2.530668 | 5.72E-205 | 2.12E-204 |
| SLC15A2 | 4.5600083 | 6.6855925 | 2.1255841 | 3.88E-185 | 1.21E-184 |
| CREBBP | 5.312683 | 7.6178826 | 2.3051996 | 9.93E-267 | 1.12E-265 |
| ALX3 | 0.5562822 | 2.6854601 | 2.1291779 | 4.34E-264 | 4.30E-263 |
| PTAR1 | 4.3410115 | 6.3652062 | 2.0241947 | 3.30E-279 | 1.17E-277 |
| SEMA3D | 1.4812192 | 3.6588463 | 2.1776271 | 4.69E-156 | 1.20E-155 |
| RGS7 | 6.0212142 | 3.6907666 | -2.330448 | 8.04E-205 | 2.97E-204 |
| LGI4 | 7.1760972 | 4.810023 | -2.366074 | 8.98E-210 | 3.48E-209 |
| HRH3 | 5.8126893 | 3.1417561 | -2.670933 | 5.18E-175 | 1.50E-174 |
| C1orf94 | 1.106293 | 3.1129475 | 2.0066545 | 3.50E-246 | 2.15E-245 |
| SST | 6.3519327 | 2.9393548 | -3.412578 | 6.58E-100 | 1.22E-99 |
| SMAD5 | 4.4226075 | 6.8154766 | 2.3928691 | 6.29E-282 | 4.46E-280 |
| 12-Sep | 2.5543422 | 0.2897525 | -2.26459 | 7.97E-241 | 4.50E-240 |
| ODF3B | 4.2683791 | 2.2296721 | -2.038707 | 2.48E-168 | 6.87E-168 |
| TRPS1 | 3.1286894 | 5.786626 | 2.6579366 | 9.44E-282 | 6.24E-280 |
| YPEL4 | 6.4675872 | 4.2794517 | -2.188135 | 1.53E-224 | 7.01E-224 |
| PRTG | 1.3655029 | 3.8664637 | 2.5009608 | 1.92E-269 | 2.49E-268 |
| MBP | 13.481463 | 10.549339 | -2.932123 | 2.46E-145 | 5.86E-145 |
| FBXL18 | 3.6160545 | 5.7224559 | 2.1064014 | 1.06E-272 | 1.72E-271 |
| DISC1 | 2.9850484 | 5.2344586 | 2.2494102 | 9.62E-272 | 1.44E-270 |
| FN1 | 7.016439 | 9.4953087 | 2.4788698 | 4.25E-207 | 1.60E-206 |
| NFIA | 5.1228434 | 7.6277816 | 2.5049382 | 5.31E-266 | 5.78E-265 |
| MYH7B | 5.0756307 | 3.0288674 | -2.046763 | 2.07E-232 | 1.04E-231 |
| PCDHGC3 | 9.1291972 | 11.43243 | 2.3032326 | 1.03E-268 | 1.29E-267 |
| DOC2B | 5.7252168 | 1.6894236 | -4.035793 | 1.45E-280 | 6.95E-279 |
| NRGN | 9.0207118 | 6.1741401 | -2.846572 | 1.86E-64 | 2.89E-64 |
| NME5 | 5.4305991 | 3.4305567 | -2.000042 | 3.96E-230 | 1.93E-229 |
| FAAH2 | 3.6889827 | 1.4698465 | -2.219136 | 1.18E-269 | 1.54E-268 |
| FGF17 | 5.4753405 | 2.3301265 | -3.145214 | 3.25E-231 | 1.61E-230 |
| NTN1 | 3.522661 | 7.2222588 | 3.6995978 | 1.45E-277 | 4.06E-276 |
| CD68 | 4.3809526 | 7.0657102 | 2.6847576 | 2.90E-192 | 9.62E-192 |
| MKI67 | 0.3693613 | 4.8928622 | 4.5235009 | 2.16E-280 | 9.92E-279 |
| CPNE6 | 7.3915749 | 3.8804066 | -3.511168 | 1.09E-230 | 5.34E-230 |
| TRIM69 | 6.2087178 | 3.2166117 | -2.992106 | 5.44E-282 | 3.93E-280 |
| SMOC1 | 5.787431 | 8.8291181 | 3.0416872 | 5.07E-121 | 1.06E-120 |
| FAM153B | 6.1444455 | 0.5818225 | -5.562623 | 1.20E-279 | 4.70E-278 |
| ZNF217 | 2.5251247 | 4.5911666 | 2.0660419 | 1.53E-255 | 1.15E-254 |
| MSR1 | 2.5350113 | 4.8599101 | 2.3248988 | 1.92E-150 | 4.71E-150 |
| TPPP3 | 7.8461378 | 5.6777826 | -2.168355 | 2.14E-138 | 4.93E-138 |
| NPY | 5.9173279 | 3.5019389 | -2.415389 | 9.03E-78 | 1.50E-77 |
| KLHL6 | 1.3477197 | 3.4821933 | 2.1344737 | 2.19E-214 | 8.93E-214 |
| CCDC73 | 0.6230545 | 3.225806 | 2.6027514 | 2.20E-277 | 5.97E-276 |
| AFF4 | 5.3182699 | 7.663771 | 2.3455012 | 7.62E-281 | 3.85E-279 |
| PABPC1L2A | 3.9785198 | 1.2676698 | -2.71085 | 2.62E-258 | 2.11E-257 |
| GRM4 | 4.8922806 | 1.9987383 | -2.893542 | 7.53E-145 | 1.79E-144 |
| SYCP3 | 2.2994824 | 0.0936445 | -2.205838 | 3.12E-275 | 6.30E-274 |
| SNORA63 | 3.2338141 | 0.3576337 | -2.87618 | 6.37E-26 | 7.97E-26 |
| FABP6 | 5.5341589 | 1.3281455 | -4.206013 | 1.05E-277 | 2.98E-276 |
| ANKRD9 | 5.621039 | 3.012897 | -2.608142 | 3.95E-277 | 1.03E-275 |
| CYP4X1 | 4.3582629 | 2.2513008 | -2.106962 | 6.36E-214 | 2.58E-213 |
| TSKU | 2.8327338 | 5.0237985 | 2.1910647 | 1.85E-262 | 1.72E-261 |
| ITGAE | 6.6196773 | 4.5714939 | -2.048183 | 1.02E-265 | 1.09E-264 |
| DUSP23 | 6.2732163 | 3.7585627 | -2.514654 | 9.17E-267 | 1.04E-265 |
| CPLX1 | 8.1409121 | 5.21228 | -2.928632 | 2.92E-208 | 1.12E-207 |
| RALYL | 6.2323675 | 3.2965213 | -2.935846 | 3.05E-239 | 1.68E-238 |
| ST8SIA4 | 2.0896464 | 4.3249871 | 2.2353407 | 1.04E-261 | 9.41E-261 |
| CDC26 | 6.0316099 | 2.8637494 | -3.16786 | 8.91E-282 | 5.96E-280 |
| ATP6V1G2 | 9.024964 | 6.8941695 | -2.130795 | 5.16E-196 | 1.76E-195 |
| PMP2 | 8.0450633 | 10.750563 | 2.7054999 | 1.44E-236 | 7.64E-236 |
| HJURP | 0.530069 | 3.1139199 | 2.5838509 | 5.58E-256 | 4.23E-255 |
| RPL12 | 10.304807 | 8.2326154 | -2.072192 | 9.75E-264 | 9.54E-263 |
| SPON1 | 5.3407268 | 7.6526478 | 2.311921 | 1.54E-159 | 4.01E-159 |
| ROMO1 | 7.9633438 | 5.9289771 | -2.034367 | 8.70E-234 | 4.46E-233 |
| LRP10 | 5.4863502 | 7.6572994 | 2.1709492 | 1.21E-260 | 1.05E-259 |
| MYBL2 | 0.399373 | 3.7826707 | 3.3832976 | 2.02E-263 | 1.95E-262 |
| CAMKV | 6.8169045 | 4.7678921 | -2.049012 | 1.49E-77 | 2.48E-77 |
| PRKX | 3.8241605 | 6.0138402 | 2.1896797 | 9.56E-227 | 4.48E-226 |
| QTRT1 | 7.8608437 | 5.8315575 | -2.029286 | 5.33E-263 | 5.05E-262 |
| SCN3A | 4.1409399 | 6.3988544 | 2.2579145 | 5.09E-188 | 1.63E-187 |
| ZFHX4 | 2.7220883 | 5.6390004 | 2.916912 | 5.79E-274 | 1.02E-272 |
| SPTBN4 | 7.5018684 | 5.3489915 | -2.152877 | 1.72E-206 | 6.45E-206 |
| RPL35 | 10.963524 | 8.6891399 | -2.274384 | 7.97E-267 | 9.04E-266 |
| ZNF490 | 1.6861258 | 4.505309 | 2.8191831 | 7.51E-278 | 2.20E-276 |
| VIPR2 | 2.7836612 | 5.4881802 | 2.704519 | 1.23E-128 | 2.66E-128 |
| LY6E | 8.5731751 | 6.495452 | -2.077723 | 1.78E-232 | 8.94E-232 |
| RPP21 | 7.2267071 | 4.5141843 | -2.712523 | 2.06E-277 | 5.61E-276 |
| SERGEF | 7.9750204 | 5.1639307 | -2.81109 | 8.06E-279 | 2.68E-277 |
| NEIL1 | 7.2004043 | 3.4301062 | -3.770298 | 4.71E-284 | 1.03E-281 |
| WIPF3 | 5.2939919 | 2.3290243 | -2.964968 | 1.79E-257 | 1.42E-256 |
| ANKRD22 | 0.9629535 | 3.1633687 | 2.2004151 | 3.31E-191 | 1.09E-190 |
| SATB2 | 3.1516772 | 5.3940275 | 2.2423503 | 9.66E-178 | 2.86E-177 |
| ABL1 | 5.8486992 | 7.8798599 | 2.0311607 | 1.39E-274 | 2.60E-273 |
| SMU1 | 4.9282162 | 6.9631717 | 2.0349555 | 4.70E-284 | 1.03E-281 |
| VIM | 8.7698289 | 10.780624 | 2.0107947 | 1.45E-103 | 2.74E-103 |
| KIF2C | 1.458455 | 3.6409152 | 2.1824602 | 9.70E-228 | 4.60E-227 |
| PPP2R2C | 7.9020023 | 5.6931603 | -2.208842 | 1.69E-154 | 4.26E-154 |
| USP9X | 5.3433072 | 7.9192161 | 2.5759089 | 2.34E-282 | 1.91E-280 |
| CD93 | 2.4869347 | 5.290542 | 2.8036073 | 3.65E-202 | 1.32E-201 |
| DSCAM | 3.7293094 | 5.9020287 | 2.1727192 | 4.09E-187 | 1.30E-186 |
| TAGAP | 1.7769332 | 3.8245591 | 2.0476259 | 4.54E-209 | 1.75E-208 |
| DPY30 | 7.3557853 | 5.329092 | -2.026693 | 1.86E-270 | 2.57E-269 |
| PABPC3 | 0.7350105 | 3.9210688 | 3.1860583 | 1.60E-281 | 9.75E-280 |
| PIGY | 1.7285183 | 7.0876483 | 5.35913 | 1.57E-283 | 2.34E-281 |
| RNASEK | 9.6877785 | 7.4061499 | -2.281629 | 4.50E-276 | 1.02E-274 |
| HK2 | 2.5106268 | 5.7107419 | 3.2001151 | 1.61E-253 | 1.16E-252 |
| COL9A3 | 6.8970631 | 4.648618 | -2.248445 | 4.20E-144 | 9.95E-144 |
| RPL31 | 11.923476 | 9.4302321 | -2.493244 | 3.42E-272 | 5.27E-271 |
| DEGS2 | 4.5857615 | 2.5703416 | -2.01542 | 1.89E-216 | 7.89E-216 |
| CACNG8 | 4.6538439 | 1.415067 | -3.238777 | 3.32E-246 | 2.04E-245 |
| PYDC1 | 3.7528887 | 0.5038714 | -3.249017 | 1.34E-255 | 1.00E-254 |
| DMKN | 5.670387 | 1.6932439 | -3.977143 | 4.97E-280 | 2.05E-278 |
| MIB1 | 4.5537095 | 6.999194 | 2.4454845 | 1.00E-280 | 5.02E-279 |
| PPAN-P2RY11 | 0.8818386 | 3.0798452 | 2.1980066 | 2.12E-157 | 5.44E-157 |
| H2AFJ | 6.2209363 | 4.1623352 | -2.058601 | 1.94E-239 | 1.07E-238 |
| SPOCD1 | 2.0077945 | 4.3413456 | 2.3335512 | 1.16E-131 | 2.57E-131 |
| CCDC103 | 2.9002456 | 5.4558188 | 2.5555732 | 5.70E-252 | 3.93E-251 |
| PTPRJ | 4.2702188 | 6.4167285 | 2.1465097 | 2.47E-278 | 7.67E-277 |
| TCEAL6 | 7.2146141 | 3.9493127 | -3.265301 | 3.87E-275 | 7.70E-274 |
| CPXM1 | 1.3567928 | 6.0356512 | 4.6788584 | 1.93E-279 | 7.13E-278 |
| TLR7 | 1.2166103 | 4.0261081 | 2.8094978 | 3.42E-253 | 2.43E-252 |
| GRM5 | 2.0710069 | 4.1891305 | 2.1181237 | 5.72E-145 | 1.36E-144 |
| TSPAN11 | 3.6225932 | 6.7531138 | 3.1305206 | 5.65E-263 | 5.35E-262 |
| FAM27B | 2.8276906 | 0.0959004 | -2.73179 | 3.45E-163 | 9.22E-163 |
| FMNL3 | 4.7278439 | 6.8753518 | 2.1475079 | 5.91E-281 | 3.08E-279 |
| AGRN | 6.3832379 | 8.4328822 | 2.0496444 | 1.16E-231 | 5.79E-231 |
| FXYD1 | 8.9562974 | 3.9494355 | -5.006862 | 3.88E-279 | 1.36E-277 |
| GALT | 6.8862347 | 4.4779049 | -2.40833 | 8.28E-280 | 3.31E-278 |
| HPCAL1 | 8.1827165 | 5.9068349 | -2.275882 | 5.64E-221 | 2.47E-220 |
| SCG5 | 8.6307093 | 6.5858296 | -2.04488 | 4.70E-219 | 2.02E-218 |
| USP38 | 3.6450178 | 5.849514 | 2.2044961 | 7.49E-284 | 1.37E-281 |
| MYL5 | 6.0076066 | 3.735505 | -2.272102 | 1.56E-274 | 2.91E-273 |
| NECAB2 | 6.7880719 | 4.3421707 | -2.445901 | 5.46E-115 | 1.10E-114 |
| PAK2 | 5.3503203 | 7.6457275 | 2.2954072 | 2.49E-284 | 6.80E-282 |
| UHRF1 | 2.0676897 | 5.237219 | 3.1695293 | 1.03E-272 | 1.66E-271 |
| FLNC | 3.2973896 | 5.9853642 | 2.6879745 | 1.33E-190 | 4.33E-190 |
| ROGDI | 8.567943 | 6.0531539 | -2.514789 | 5.64E-271 | 8.12E-270 |
| ASTN1 | 5.8190609 | 8.2697856 | 2.4507247 | 1.21E-258 | 9.84E-258 |
| PRR4 | 5.3183044 | 2.93721 | -2.381094 | 6.67E-283 | 6.88E-281 |
| BEST3 | 2.9547047 | 5.0472592 | 2.0925544 | 3.40E-158 | 8.77E-158 |
| PRSS3 | 6.1280385 | 2.3814648 | -3.746574 | 4.74E-265 | 4.93E-264 |
| ID4 | 6.5599731 | 8.775999 | 2.216026 | 1.82E-192 | 6.05E-192 |
| ENOSF1 | 6.0339173 | 3.6008489 | -2.433068 | 3.91E-252 | 2.71E-251 |
| ZFP36L2 | 5.5854753 | 7.8771877 | 2.2917124 | 3.65E-245 | 2.20E-244 |
| SEL1L | 5.1849358 | 7.369388 | 2.1844522 | 1.88E-280 | 8.76E-279 |
| MTHFD2 | 4.2822097 | 6.737969 | 2.4557594 | 3.77E-200 | 1.34E-199 |
| AP2B1 | 7.6833818 | 9.9913183 | 2.3079366 | 6.38E-283 | 6.69E-281 |
| ZNFX1 | 4.663543 | 6.8451296 | 2.1815866 | 1.19E-282 | 1.09E-280 |
| ZDHHC15 | 2.4010416 | 4.5871811 | 2.1861395 | 1.98E-279 | 7.28E-278 |
| NSD1 | 4.7575405 | 6.7576364 | 2.0000959 | 2.47E-273 | 4.16E-272 |
| MAPK15 | 3.138543 | 0.9337902 | -2.204753 | 4.25E-126 | 9.10E-126 |
| CCDC78 | 5.5750839 | 3.07735 | -2.497734 | 7.35E-250 | 4.85E-249 |
| NDUFAB1 | 8.1972938 | 6.0339914 | -2.163302 | 3.68E-275 | 7.36E-274 |
| E2F2 | 0.1654482 | 2.9216798 | 2.7562317 | 1.86E-277 | 5.09E-276 |
| KIF11 | 1.3229336 | 4.1909064 | 2.8679728 | 6.27E-282 | 4.46E-280 |
| SNORA61 | 2.3035469 | 0.115326 | -2.188221 | 1.49E-12 | 1.71E-12 |
| CCDC28A | 6.6076462 | 4.537404 | -2.070242 | 1.60E-277 | 4.44E-276 |
| MASP1 | 3.9864553 | 7.193497 | 3.2070417 | 1.03E-242 | 5.97E-242 |
| ESYT3 | 3.8894593 | 1.7409994 | -2.14846 | 2.01E-240 | 1.13E-239 |
| FSTL1 | 5.0115153 | 7.1475106 | 2.1359953 | 6.13E-199 | 2.15E-198 |
| SPHAR | 0.4727701 | 3.7489282 | 3.2761581 | 2.42E-219 | 1.04E-218 |
| NOTCH3 | 4.6771252 | 6.9239692 | 2.2468441 | 8.20E-245 | 4.92E-244 |
| COL5A2 | 3.0148912 | 5.2827146 | 2.2678235 | 1.97E-151 | 4.88E-151 |
| MYLPF | 2.5052551 | 0.2547849 | -2.25047 | 3.87E-271 | 5.62E-270 |
| SNHG3 | 4.9277061 | 2.5714711 | -2.356235 | 8.02E-280 | 3.22E-278 |
| RPL23P8 | 0.4179367 | 2.711255 | 2.2933182 | 2.00E-257 | 1.57E-256 |
| COL3A1 | 2.1754863 | 5.3783328 | 3.2028465 | 5.89E-203 | 2.14E-202 |
| LRRC8A | 6.9828576 | 9.0863483 | 2.1034907 | 5.04E-239 | 2.77E-238 |
| C1QL4 | 0.8078674 | 3.0278883 | 2.2200209 | 7.41E-214 | 3.00E-213 |
| CACNG3 | 4.8607433 | 2.8305364 | -2.030207 | 9.88E-83 | 1.69E-82 |
| SMG1 | 5.015604 | 7.1808671 | 2.1652631 | 6.34E-249 | 4.11E-248 |
| ADAP1 | 7.7760606 | 5.2403619 | -2.535699 | 5.20E-244 | 3.09E-243 |
| RIN2 | 4.8469423 | 7.0061141 | 2.1591717 | 6.60E-262 | 6.01E-261 |
| RAB3C | 5.0097148 | 2.3440736 | -2.665641 | 6.62E-222 | 2.94E-221 |
| TNFRSF19 | 2.2899072 | 5.307329 | 3.0174218 | 9.41E-277 | 2.33E-275 |
| PXK | 6.9644488 | 4.8936079 | -2.070841 | 1.20E-267 | 1.42E-266 |
| HIST1H4E | 3.320283 | 0.9023692 | -2.417914 | 2.76E-229 | 1.33E-228 |
| MRPL34 | 7.5354164 | 5.2882813 | -2.247135 | 1.05E-270 | 1.48E-269 |
| CYBB | 3.1618021 | 6.1513574 | 2.9895553 | 1.87E-234 | 9.66E-234 |
| SH3PXD2B | 4.2536939 | 6.8276425 | 2.5739486 | 9.38E-279 | 3.08E-277 |
| FOXO3B | 1.34067 | 5.8801462 | 4.5394762 | 2.42E-283 | 3.25E-281 |
| ARL6IP4 | 9.1817276 | 7.0832716 | -2.098456 | 2.81E-271 | 4.10E-270 |
| C6orf136 | 7.0251713 | 4.8369119 | -2.188259 | 1.09E-279 | 4.29E-278 |
| MSTN | 1.6645502 | 4.0855845 | 2.4210343 | 1.03E-207 | 3.93E-207 |
| STON1 | 2.7821342 | 5.5952293 | 2.8130952 | 4.51E-232 | 2.25E-231 |
| TTC9B | 7.3973456 | 3.5526209 | -3.844725 | 6.59E-265 | 6.79E-264 |
| KCNH3 | 6.3875378 | 4.0974173 | -2.290121 | 1.21E-136 | 2.76E-136 |
| ADORA3 | 3.3738932 | 5.7521926 | 2.3782994 | 4.97E-178 | 1.47E-177 |
| POLE4 | 7.3212887 | 4.9908017 | -2.330487 | 1.25E-232 | 6.30E-232 |
| TMEM141 | 7.3185803 | 5.1542452 | -2.164335 | 5.98E-264 | 5.91E-263 |
| SEMA6A | 5.649714 | 7.7258932 | 2.0761791 | 3.11E-242 | 1.80E-241 |
| PHYHIP | 8.6596269 | 5.9195312 | -2.740096 | 9.55E-150 | 2.34E-149 |
| MAP1B | 7.6873585 | 10.297365 | 2.6100064 | 3.58E-264 | 3.56E-263 |
| RIT2 | 5.4458859 | 2.3522495 | -3.093636 | 2.67E-228 | 1.27E-227 |
| FXYD7 | 7.9203614 | 3.735033 | -4.185328 | 4.04E-234 | 2.08E-233 |
| OAS3 | 3.4721151 | 5.6894614 | 2.2173462 | 6.70E-240 | 3.73E-239 |
| PIK3AP1 | 2.1122191 | 4.7001411 | 2.587922 | 6.56E-248 | 4.19E-247 |
| SNRPA1 | 6.6611282 | 4.5137676 | -2.147361 | 4.23E-278 | 1.29E-276 |
| HSFX1 | 2.5697832 | 0.0010324 | -2.568751 | 1.90E-281 | 1.13E-279 |
| LHFPL3 | 4.2455742 | 7.3917838 | 3.1462096 | 7.25E-200 | 2.56E-199 |
| ETV1 | 5.6613066 | 8.6178945 | 2.9565879 | 5.54E-197 | 1.91E-196 |
| LY6H | 7.5280241 | 4.7875744 | -2.74045 | 1.61E-113 | 3.23E-113 |
| RREB1 | 2.2335715 | 4.2569158 | 2.0233444 | 1.44E-269 | 1.87E-268 |
| HBA1 | 8.7858529 | 4.9014529 | -3.8844 | 1.40E-216 | 5.82E-216 |
| RGN | 6.2787074 | 3.9452506 | -2.333457 | 2.68E-247 | 1.69E-246 |
| HIP1 | 5.1238751 | 7.3674473 | 2.2435722 | 9.77E-241 | 5.51E-240 |
| RHBDL1 | 6.1043667 | 3.33537 | -2.768997 | 3.41E-257 | 2.66E-256 |
| ENY2 | 7.1043977 | 4.9531208 | -2.151277 | 3.32E-273 | 5.55E-272 |
| MKLN1 | 4.9782762 | 7.8185656 | 2.8402895 | 4.24E-283 | 4.75E-281 |
| GCSH | 7.5434046 | 2.7798114 | -4.763593 | 2.95E-282 | 2.31E-280 |
| ABHD2 | 5.6160576 | 8.398188 | 2.7821304 | 1.25E-278 | 4.00E-277 |
| PTPN5 | 6.536377 | 3.9518951 | -2.584482 | 2.29E-109 | 4.47E-109 |
| COL4A1 | 3.7107854 | 6.7973101 | 3.0865246 | 1.32E-197 | 4.58E-197 |
| ST6GAL1 | 5.0073342 | 7.1488156 | 2.1414813 | 2.65E-245 | 1.60E-244 |
| P2RX6P | 3.3766437 | 0.008896 | -3.367748 | 6.50E-272 | 9.83E-271 |
| SIGLEC1 | 1.4913452 | 3.745904 | 2.2545588 | 9.79E-233 | 4.95E-232 |
| POM121 | 5.5787604 | 7.8416604 | 2.2629 | 2.84E-282 | 2.25E-280 |
| PPP1R1A | 6.8558467 | 4.2884946 | -2.567352 | 4.05E-188 | 1.30E-187 |
| DTL | 0.6259094 | 3.5391156 | 2.9132062 | 7.44E-276 | 1.62E-274 |
| KCNK1 | 6.2969676 | 4.0890751 | -2.207892 | 1.02E-175 | 2.99E-175 |
| ANKRD36BP1 | 0.0373745 | 2.9742688 | 2.9368944 | 1.32E-279 | 5.08E-278 |
| IFI27 | 8.0817774 | 5.411845 | -2.669932 | 5.89E-235 | 3.06E-234 |
| IGSF3 | 2.7002638 | 5.695462 | 2.9951982 | 2.69E-258 | 2.17E-257 |
| ITGB8 | 4.7589749 | 8.189241 | 3.4302661 | 7.30E-274 | 1.28E-272 |
| AK1 | 8.8744366 | 6.6899458 | -2.184491 | 8.48E-253 | 5.97E-252 |
| SCIN | 2.7874038 | 4.8878163 | 2.1004125 | 3.20E-122 | 6.72E-122 |
| SYK | 2.226499 | 5.0848269 | 2.8583279 | 2.06E-250 | 1.38E-249 |
| C1orf226 | 3.1089159 | 5.4447725 | 2.3358567 | 8.44E-252 | 5.81E-251 |
| NPAS3 | 4.793505 | 6.9791285 | 2.1856236 | 2.98E-256 | 2.28E-255 |
| UBE2C | 1.0624173 | 3.7132819 | 2.6508646 | 2.08E-207 | 7.88E-207 |
| SIPA1L2 | 4.7675171 | 6.7773084 | 2.0097913 | 1.20E-261 | 1.09E-260 |
| CDCA5 | 2.2563153 | 4.2695295 | 2.0132142 | 2.34E-214 | 9.56E-214 |
| CSF1R | 4.9697917 | 7.8532664 | 2.8834748 | 2.01E-216 | 8.37E-216 |
| SNHG6 | 9.0905051 | 6.2120184 | -2.878487 | 4.50E-274 | 8.08E-273 |
| BEX5 | 7.4141399 | 3.5886628 | -3.825477 | 8.04E-274 | 1.40E-272 |
| HSD17B3 | 3.6944151 | 1.6320516 | -2.062363 | 1.56E-205 | 5.81E-205 |
| SOX11 | 1.7788117 | 6.0862535 | 4.3074418 | 3.17E-282 | 2.45E-280 |
| NDUFA1 | 8.6838372 | 6.6281906 | -2.055647 | 1.74E-274 | 3.22E-273 |
| LIMD1 | 2.7428654 | 5.4576082 | 2.7147428 | 3.92E-278 | 1.20E-276 |
| CKMT2 | 4.6721849 | 2.4379736 | -2.234211 | 1.09E-264 | 1.11E-263 |
| NRN1L | 3.8619956 | 1.7936871 | -2.068309 | 2.62E-251 | 1.79E-250 |
| RAVER1 | 4.5839976 | 6.980141 | 2.3961434 | 2.11E-284 | 6.23E-282 |
| DAG1 | 5.6840152 | 8.4830091 | 2.7989939 | 6.21E-285 | 3.32E-282 |
| RARRES2 | 6.9347065 | 3.6694786 | -3.265228 | 5.81E-195 | 1.96E-194 |
| PSD | 8.7217826 | 6.3862678 | -2.335515 | 3.16E-137 | 7.21E-137 |
| PLAC9 | 4.6452492 | 2.4111052 | -2.234144 | 3.36E-257 | 2.62E-256 |
| TCTEX1D2 | 6.2930097 | 4.0107575 | -2.282252 | 4.00E-277 | 1.04E-275 |
| VANGL1 | 1.4168532 | 4.0321435 | 2.6152903 | 3.07E-280 | 1.34E-278 |
| WDR35 | 3.6957734 | 5.8275825 | 2.1318091 | 9.15E-285 | 3.78E-282 |
| AMT | 6.1430135 | 4.1383874 | -2.004626 | 2.91E-246 | 1.79E-245 |
| MXRA5 | 0.7656752 | 3.8744562 | 3.1087811 | 3.70E-279 | 1.31E-277 |
| ITGAV | 5.8195515 | 8.7292247 | 2.9096732 | 2.30E-283 | 3.17E-281 |
| HNRNPA0 | 5.3701244 | 8.2265963 | 2.856472 | 5.65E-285 | 3.32E-282 |
| WFDC2 | 4.9929508 | 1.6998687 | -3.293082 | 3.02E-280 | 1.32E-278 |
| ZEB1 | 5.7976432 | 8.38785 | 2.5902068 | 7.99E-278 | 2.33E-276 |
| SLC12A5 | 8.2580181 | 4.4231429 | -3.834875 | 4.15E-213 | 1.67E-212 |
| GRIK3 | 3.8555611 | 7.5656087 | 3.7100476 | 2.79E-263 | 2.66E-262 |
| GNAI3 | 2.8599553 | 7.0404288 | 4.1804735 | 1.72E-285 | 3.32E-282 |
| PHLPP1 | 5.8669071 | 8.114154 | 2.2472469 | 9.61E-256 | 7.22E-255 |
| SLC9A3R2 | 7.9722326 | 5.9507132 | -2.021519 | 5.45E-264 | 5.39E-263 |
| SMC2 | 3.3327818 | 5.6230838 | 2.290302 | 8.43E-284 | 1.49E-281 |
| MELK | 0.3187232 | 2.9798407 | 2.6611175 | 5.07E-260 | 4.29E-259 |
| KIAA1549 | 2.6893675 | 6.2780962 | 3.5887288 | 3.06E-280 | 1.34E-278 |
| SNORA18 | 2.0820229 | 0.0634788 | -2.018544 | 6.30E-11 | 7.08E-11 |
| C3orf14 | 6.8858575 | 4.0611297 | -2.824728 | 1.49E-281 | 9.27E-280 |
| IQGAP3 | 0.6252377 | 3.3423728 | 2.7171351 | 7.58E-260 | 6.38E-259 |
| PRKCG | 6.1256072 | 3.3404943 | -2.785113 | 5.22E-149 | 1.27E-148 |
| DLGAP3 | 6.2233348 | 4.0681086 | -2.155226 | 9.77E-165 | 2.64E-164 |
| GNB3 | 5.4463436 | 3.1812126 | -2.265131 | 1.75E-202 | 6.35E-202 |
| CALCRL | 3.4055694 | 6.8157115 | 3.4101421 | 5.19E-260 | 4.40E-259 |
| OPALIN | 6.5711867 | 3.7766404 | -2.794546 | 1.18E-120 | 2.45E-120 |
| KIF26A | 2.0827315 | 4.1499624 | 2.0672309 | 8.97E-206 | 3.35E-205 |
| SPINT2 | 7.3878498 | 4.7857394 | -2.60211 | 2.09E-264 | 2.11E-263 |
| FOXM1 | 2.0411736 | 4.9675769 | 2.9264033 | 1.45E-262 | 1.36E-261 |
| F2R | 1.7084149 | 6.5863885 | 4.8779736 | 5.72E-285 | 3.32E-282 |
| NUFIP2 | 4.7811236 | 7.2149282 | 2.4338047 | 1.76E-280 | 8.23E-279 |
| PCBD1 | 7.4028785 | 4.9919891 | -2.410889 | 1.60E-276 | 3.84E-275 |
| ATP7A | 1.9371623 | 4.2087912 | 2.271629 | 2.34E-281 | 1.37E-279 |
| SLIT1 | 4.94752 | 7.818918 | 2.871398 | 6.80E-165 | 1.84E-164 |
| BRSK2 | 7.5972137 | 5.5094092 | -2.087804 | 1.29E-233 | 6.61E-233 |
| FBXO30 | 3.0872705 | 5.1545401 | 2.0672696 | 1.36E-282 | 1.23E-280 |
| PDE1B | 6.5517089 | 4.3717121 | -2.179997 | 3.63E-146 | 8.71E-146 |
| DTX3L | 3.2029672 | 5.7522494 | 2.5492822 | 4.73E-268 | 5.74E-267 |
| OSGIN2 | 4.6200257 | 6.6433427 | 2.023317 | 1.08E-260 | 9.35E-260 |
| AKAP13 | 4.9759889 | 7.1749091 | 2.1989203 | 2.23E-272 | 3.51E-271 |
| GOLT1A | 4.2578291 | 1.0690383 | -3.188791 | 1.36E-253 | 9.74E-253 |
| DNASE1 | 4.8795277 | 2.8445996 | -2.034928 | 1.41E-276 | 3.41E-275 |
| TROAP | 0.7110657 | 2.9016973 | 2.1906316 | 1.85E-221 | 8.14E-221 |
| LRRC55 | 2.0478149 | 5.3399643 | 3.2921494 | 3.39E-255 | 2.52E-254 |
| C4orf36 | 3.333429 | 1.2344463 | -2.098983 | 1.54E-268 | 1.91E-267 |
| TNNC2 | 4.1680448 | 1.1753248 | -2.99272 | 1.19E-280 | 5.90E-279 |
| FBN2 | 0.5620728 | 3.6318259 | 3.0697531 | 9.59E-269 | 1.20E-267 |
| ARHGDIG | 8.3893547 | 4.2375982 | -4.151757 | 7.07E-273 | 1.15E-271 |
| CLIP2 | 7.1763629 | 9.2737606 | 2.0973978 | 2.00E-265 | 2.12E-264 |
| SOX6 | 3.2710459 | 6.9518421 | 3.6807963 | 5.50E-276 | 1.23E-274 |
| MEST | 4.6913612 | 6.7570509 | 2.0656896 | 6.26E-212 | 2.49E-211 |
| MED13 | 4.3056242 | 7.1372692 | 2.8316451 | 7.56E-282 | 5.20E-280 |
| C4A | 6.88868 | 9.093792 | 2.2051119 | 1.29E-137 | 2.96E-137 |
| HSPA1B | 9.0255942 | 6.1274343 | -2.89816 | 7.37E-213 | 2.96E-212 |
| SPRED3 | 3.8258271 | 1.3045508 | -2.521276 | 1.61E-267 | 1.89E-266 |
| STEAP3 | 2.9085389 | 4.9734384 | 2.0648995 | 1.72E-141 | 4.03E-141 |
| PAG1 | 3.6510969 | 6.2622676 | 2.6111707 | 4.67E-247 | 2.93E-246 |
| PLVAP | 2.1027737 | 4.6109821 | 2.5082083 | 3.14E-246 | 1.93E-245 |
| KLHL25 | 4.0336791 | 6.2425528 | 2.2088737 | 1.35E-258 | 1.09E-257 |
| SNAPC5 | 6.7928496 | 3.673969 | -3.118881 | 9.87E-285 | 3.86E-282 |
| MAPK1 | 6.5889285 | 8.8621774 | 2.2732489 | 2.28E-275 | 4.70E-274 |
| HIGD2A | 8.2458249 | 6.2360059 | -2.009819 | 9.38E-270 | 1.23E-268 |
| SH2D5 | 5.0870489 | 2.9333668 | -2.153682 | 7.84E-119 | 1.61E-118 |
| FAM169A | 3.7828449 | 6.2019895 | 2.4191445 | 4.45E-265 | 4.64E-264 |
| PURB | 5.0082511 | 7.3272241 | 2.3189729 | 5.31E-282 | 3.87E-280 |
| TAF13 | 6.8619464 | 4.1345003 | -2.727446 | 1.29E-280 | 6.27E-279 |
| PPDPF | 9.2901055 | 6.9421006 | -2.348005 | 5.95E-271 | 8.50E-270 |
| TSIX | 0.1182888 | 2.2299472 | 2.1116584 | 3.32E-189 | 1.07E-188 |
| PNCK | 7.1290735 | 3.9943388 | -3.134735 | 8.24E-251 | 5.59E-250 |
| NDUFB7 | 9.0977915 | 6.8345936 | -2.263198 | 1.32E-261 | 1.19E-260 |
| KCNJ9 | 6.8833197 | 4.7983535 | -2.084966 | 5.19E-188 | 1.66E-187 |
| YJEFN3 | 7.85687 | 4.0664608 | -3.790409 | 2.40E-270 | 3.27E-269 |
| MATK | 5.8670008 | 3.3348122 | -2.532189 | 1.16E-165 | 3.16E-165 |
| SCD5 | 8.8286005 | 10.847421 | 2.0188204 | 2.47E-232 | 1.24E-231 |
| PRRG3 | 3.5613593 | 0.7708448 | -2.790514 | 3.19E-210 | 1.24E-209 |
| TOMM7 | 10.146012 | 7.4657234 | -2.680288 | 1.93E-275 | 4.01E-274 |
| E2F7 | 0.3619413 | 2.5012773 | 2.1393361 | 1.82E-257 | 1.44E-256 |
| SIGLEC8 | 2.3612084 | 4.8746416 | 2.5134332 | 1.16E-199 | 4.08E-199 |
| IGF2R | 4.1646668 | 6.9100694 | 2.7454026 | 4.58E-283 | 5.05E-281 |
| TMEM119 | 3.0373135 | 5.3338817 | 2.2965681 | 1.94E-185 | 6.08E-185 |
| BCL2L11 | 2.9383344 | 4.988759 | 2.0504246 | 6.70E-273 | 1.10E-271 |
| ATRN | 5.1890323 | 7.5276512 | 2.338619 | 4.96E-280 | 2.05E-278 |
| RTKN2 | 0.9541183 | 3.1186388 | 2.1645205 | 6.46E-255 | 4.76E-254 |
| TCF4 | 6.1327986 | 8.6669585 | 2.53416 | 3.48E-277 | 9.14E-276 |
| GLG1 | 6.7023163 | 8.759638 | 2.0573217 | 3.70E-282 | 2.79E-280 |
| PCDH17 | 4.1256096 | 6.9182305 | 2.7926209 | 1.75E-273 | 2.98E-272 |
| SCARB2 | 7.1621671 | 9.5288732 | 2.366706 | 1.24E-280 | 6.08E-279 |
| UHRF1BP1 | 3.2836224 | 5.478613 | 2.1949906 | 4.53E-280 | 1.91E-278 |
| SLC27A5 | 6.4791331 | 4.1226252 | -2.356508 | 1.91E-273 | 3.24E-272 |
| OLFML3 | 3.706379 | 5.7574358 | 2.0510568 | 4.94E-185 | 1.54E-184 |
| SERPINE1 | 2.5539763 | 5.2801018 | 2.7261255 | 1.70E-152 | 4.24E-152 |
| DGCR10 | 4.0222192 | 1.6440516 | -2.378168 | 1.21E-174 | 3.49E-174 |
| BCO2 | 4.9874407 | 2.5842517 | -2.403189 | 4.17E-275 | 8.26E-274 |
| RPL18A | 10.540067 | 6.4441859 | -4.095881 | 1.32E-274 | 2.48E-273 |
| GPC4 | 3.3285961 | 5.5329922 | 2.2043961 | 6.97E-256 | 5.26E-255 |
| PRRX1 | 4.5200413 | 6.9651205 | 2.4450792 | 2.59E-260 | 2.22E-259 |
| RSC1A1 | 0.1749547 | 3.2015339 | 3.0265792 | 2.47E-270 | 3.35E-269 |
| KCNA6 | 0.050806 | 5.8516214 | 5.8008154 | 5.12E-284 | 1.06E-281 |
| ZC3HAV1 | 3.5227599 | 6.2005694 | 2.6778095 | 7.42E-274 | 1.30E-272 |
| C9orf139 | 3.9283503 | 1.3511437 | -2.577207 | 1.26E-281 | 8.00E-280 |
| ADCY7 | 3.0869628 | 5.6932818 | 2.606319 | 2.28E-266 | 2.53E-265 |
| POMC | 3.9574639 | 1.8681852 | -2.089279 | 1.01E-243 | 5.95E-243 |
| TPD52L1 | 7.3249827 | 4.6781395 | -2.646843 | 4.16E-171 | 1.18E-170 |
| NANOS3 | 4.9650954 | 2.5379937 | -2.427102 | 9.21E-256 | 6.93E-255 |
| OLFML2B | 2.460329 | 4.6210992 | 2.1607702 | 2.95E-196 | 1.01E-195 |
| SERP2 | 7.4262504 | 4.8033448 | -2.622906 | 1.08E-276 | 2.66E-275 |
| ZC3H12C | 3.2558327 | 5.5968705 | 2.3410377 | 6.05E-283 | 6.38E-281 |
| GPR143 | 3.972233 | 1.9221014 | -2.050132 | 2.69E-222 | 1.20E-221 |
| RPL36A | 10.408673 | 2.6128763 | -7.795797 | 2.05E-282 | 1.72E-280 |
| POLR2J2 | 2.2212902 | 4.8214587 | 2.6001685 | 1.02E-238 | 5.60E-238 |
| TDRD9 | 4.2752625 | 2.0677814 | -2.207481 | 6.77E-216 | 2.80E-215 |
| CALY | 7.9635176 | 3.2535177 | -4.71 | 8.72E-260 | 7.32E-259 |
| RNF180 | 3.409329 | 5.6216866 | 2.2123576 | 6.51E-268 | 7.78E-267 |
| NFAT5 | 3.818262 | 6.5230766 | 2.7048145 | 4.04E-276 | 9.20E-275 |
| STC2 | 1.6493704 | 4.3590252 | 2.7096549 | 5.55E-233 | 2.81E-232 |
| CEP350 | 4.4744907 | 6.537221 | 2.0627303 | 3.47E-274 | 6.29E-273 |
| SNHG9 | 7.1047349 | 2.8988504 | -4.205884 | 2.70E-280 | 1.21E-278 |
| GALNT8 | 4.821141 | 1.4864208 | -3.33472 | 4.66E-280 | 1.95E-278 |
| JOSD2 | 6.9232086 | 4.6300318 | -2.293177 | 1.30E-266 | 1.46E-265 |
| ARHGEF6 | 5.3048443 | 8.0663325 | 2.7614882 | 4.02E-273 | 6.69E-272 |
| NIPAL2 | 4.5674501 | 2.2015794 | -2.365871 | 3.70E-255 | 2.74E-254 |
| UTP20 | 3.1219342 | 5.1228649 | 2.0009307 | 2.53E-282 | 2.03E-280 |
| RPS7 | 10.223652 | 7.7410352 | -2.482617 | 2.90E-272 | 4.51E-271 |
| GFAP | 12.233485 | 14.270993 | 2.0375082 | 1.60E-196 | 5.49E-196 |
| AFMID | 6.778327 | 4.5905952 | -2.187732 | 6.49E-266 | 7.01E-265 |
| RGPD4 | 0.1049408 | 3.5268932 | 3.4219523 | 1.07E-275 | 2.28E-274 |
| SFT2D3 | 0.0513736 | 4.6897171 | 4.6383435 | 1.70E-286 | 1.26E-282 |
| ALG10B | 2.1349292 | 4.5650211 | 2.4300919 | 1.49E-281 | 9.27E-280 |
| TET1 | 1.2628384 | 3.3309826 | 2.0681441 | 8.79E-254 | 6.35E-253 |
| LHFPL2 | 4.006452 | 6.1230891 | 2.1166371 | 4.14E-254 | 3.01E-253 |
| TIMP3 | 6.7324665 | 9.8348246 | 3.102358 | 1.35E-269 | 1.76E-268 |
| RPRML | 4.5818724 | 1.6285189 | -2.953353 | 4.48E-162 | 1.19E-161 |
| TTN | 1.2930117 | 3.471517 | 2.1785053 | 2.63E-262 | 2.44E-261 |
| TOX3 | 2.4513508 | 4.6002444 | 2.1488935 | 2.37E-172 | 6.76E-172 |
| GNS | 5.3916509 | 7.5637386 | 2.1720877 | 6.42E-278 | 1.90E-276 |
| ITGB2 | 4.4563005 | 6.8210826 | 2.3647821 | 2.22E-174 | 6.42E-174 |
| TNC | 2.658147 | 8.4968566 | 5.8387096 | 2.32E-276 | 5.46E-275 |
| NUSAP1 | 2.0527872 | 4.6605014 | 2.6077142 | 6.45E-234 | 3.31E-233 |
| LYPLA2P1 | 0.1855311 | 2.4748885 | 2.2893573 | 1.16E-278 | 3.75E-277 |
| MYL7 | 3.5666384 | 0.148296 | -3.418342 | 8.70E-283 | 8.49E-281 |
| COX6A1 | 10.727438 | 7.5901975 | -3.13724 | 1.65E-277 | 4.55E-276 |
| ABL2 | 3.9373099 | 6.1799523 | 2.2426424 | 9.05E-280 | 3.60E-278 |
| SLC8A1 | 3.449109 | 5.7798803 | 2.3307713 | 9.97E-266 | 1.07E-264 |
| JAG1 | 4.3844016 | 6.3991813 | 2.0147798 | 1.82E-237 | 9.83E-237 |
| ZNF204P | 5.7127759 | 3.5378488 | -2.174927 | 9.93E-229 | 4.77E-228 |
| CCDC57 | 6.6989656 | 4.6244207 | -2.074545 | 4.53E-266 | 4.96E-265 |
| OLFML2A | 2.0910254 | 4.2718795 | 2.1808541 | 1.10E-260 | 9.57E-260 |
| HBG2 | 2.9355967 | 0.7675423 | -2.168054 | 1.72E-207 | 6.51E-207 |
| CD22 | 5.7323195 | 2.8844709 | -2.847849 | 4.11E-200 | 1.46E-199 |
| FAM71E1 | 4.7897379 | 2.418799 | -2.370939 | 7.92E-272 | 1.19E-270 |
| BOLA3 | 7.0535363 | 4.0049384 | -3.048598 | 6.56E-282 | 4.63E-280 |
| GALNT10 | 4.3193573 | 6.5242442 | 2.2048868 | 3.71E-267 | 4.30E-266 |
| CBX5 | 5.7247985 | 8.9344171 | 3.2096186 | 8.50E-285 | 3.61E-282 |
| DCXR | 8.5759494 | 6.2970916 | -2.278858 | 2.24E-269 | 2.89E-268 |
| EEF1A1P9 | 0.486042 | 7.7843301 | 7.2982881 | 6.27E-284 | 1.23E-281 |
| SLC24A5 | 0.3607095 | 2.4842011 | 2.1234916 | 2.25E-271 | 3.30E-270 |
| MT1E | 8.897994 | 5.6736521 | -3.224342 | 6.33E-235 | 3.29E-234 |
| PTTG1IP | 7.5060843 | 9.8311906 | 2.3251064 | 1.79E-271 | 2.64E-270 |
| SV2C | 3.0242802 | 0.9043588 | -2.119921 | 4.40E-156 | 1.12E-155 |
| ZNF692 | 7.5826391 | 5.2519963 | -2.330643 | 1.10E-264 | 1.13E-263 |
| LAPTM4B | 5.9089117 | 7.9867192 | 2.0778075 | 5.62E-279 | 1.94E-277 |
| USP13 | 3.1628015 | 5.2055931 | 2.0427917 | 1.26E-281 | 8.00E-280 |
| TMEM88B | 4.6928771 | 1.0406252 | -3.652252 | 6.13E-247 | 3.83E-246 |
| U2AF1L4 | 6.6330941 | 4.0684918 | -2.564602 | 1.38E-280 | 6.67E-279 |
| SLC1A6 | 5.3011908 | 3.0871361 | -2.214055 | 6.88E-145 | 1.64E-144 |
| TCEAL2 | 8.8539057 | 6.7625114 | -2.091394 | 2.69E-258 | 2.17E-257 |
| CREB3L2 | 3.5712613 | 5.8986647 | 2.3274034 | 6.13E-276 | 1.35E-274 |
| CCNB2 | 0.9946223 | 3.3328336 | 2.3382113 | 5.78E-216 | 2.40E-215 |
| YPEL3 | 9.3702044 | 6.7426663 | -2.627538 | 4.18E-276 | 9.50E-275 |
| ABLIM2 | 6.5251452 | 4.2195675 | -2.305578 | 1.49E-226 | 6.95E-226 |
| CHCHD10 | 8.9862359 | 4.5215468 | -4.464689 | 1.91E-281 | 1.13E-279 |
| FKBP2 | 8.1212796 | 5.9390475 | -2.182232 | 4.56E-269 | 5.81E-268 |
| MT1H | 4.0203502 | 1.0330092 | -2.987341 | 3.68E-137 | 8.42E-137 |
| PIK3CG | 0.6404902 | 2.6883227 | 2.0478325 | 1.90E-261 | 1.70E-260 |
| PRKCZ | 8.3692847 | 5.6484628 | -2.720822 | 1.53E-256 | 1.18E-255 |
| GLI2 | 1.7794954 | 3.8397809 | 2.0602855 | 4.92E-252 | 3.41E-251 |
| LGALS1 | 9.4394535 | 7.3581116 | -2.081342 | 3.94E-150 | 9.68E-150 |
| CDK1 | 1.5465891 | 4.0278631 | 2.481274 | 1.99E-243 | 1.17E-242 |
| CRISPLD1 | 2.7644066 | 6.4063835 | 3.6419768 | 6.20E-275 | 1.21E-273 |
| KLF12 | 3.4348087 | 6.2136983 | 2.7788897 | 2.95E-282 | 2.31E-280 |
| PVALB | 6.0871738 | 1.6296545 | -4.457519 | 2.65E-189 | 8.58E-189 |
| ACYP2 | 7.5881434 | 5.2549052 | -2.333238 | 5.88E-272 | 8.91E-271 |
| KPNA4 | 5.1164619 | 7.1509745 | 2.0345125 | 1.21E-283 | 1.95E-281 |
| AP1G2 | 8.2690652 | 4.597336 | -3.671729 | 1.60E-277 | 4.44E-276 |
| HEATR1 | 3.2533351 | 5.4875924 | 2.2342573 | 1.09E-282 | 1.02E-280 |
| MRPL23 | 7.4087032 | 5.2859146 | -2.122789 | 7.58E-265 | 7.80E-264 |
| MMP19 | 1.0328868 | 5.2460542 | 4.2131674 | 4.04E-278 | 1.23E-276 |
| RAPH1 | 3.2893611 | 5.6995085 | 2.4101474 | 1.53E-278 | 4.83E-277 |
| CELF4 | 7.2802977 | 4.5235713 | -2.756726 | 8.31E-182 | 2.53E-181 |
| APC2 | 6.665233 | 9.3271677 | 2.6619347 | 1.82E-249 | 1.19E-248 |
| VWA5B2 | 6.7218778 | 3.3104903 | -3.411387 | 4.16E-256 | 3.16E-255 |
| SLC5A3 | 3.2831365 | 5.5886483 | 2.3055118 | 2.89E-266 | 3.18E-265 |
| RPL13P5 | 4.3617142 | 2.3077453 | -2.053969 | 5.70E-281 | 2.99E-279 |
| NAV2 | 4.5671401 | 7.1620667 | 2.5949266 | 1.02E-274 | 1.95E-273 |
| SYT5 | 6.8501394 | 4.1113648 | -2.738775 | 2.34E-144 | 5.55E-144 |
| LCN12 | 5.270648 | 1.9431829 | -3.327465 | 2.56E-277 | 6.89E-276 |
| ESCO2 | 0.4561826 | 2.6041695 | 2.147987 | 1.52E-257 | 1.20E-256 |
| ATF7IP2 | 4.598962 | 2.2192808 | -2.379681 | 9.13E-279 | 3.01E-277 |
| NAIP | 5.1627659 | 3.0903038 | -2.072462 | 4.79E-269 | 6.09E-268 |
| NTRK2 | 8.0006527 | 11.074968 | 3.0743155 | 5.51E-205 | 2.04E-204 |
| NRP2 | 4.5653722 | 6.7708904 | 2.2055182 | 1.82E-228 | 8.71E-228 |
| LRRN1 | 4.7897766 | 7.3273403 | 2.5375637 | 5.24E-243 | 3.05E-242 |
| BEND3 | 1.5204571 | 3.5586121 | 2.0381549 | 3.38E-262 | 3.12E-261 |
| FER1L4 | 3.4976084 | 1.4061728 | -2.091436 | 5.41E-218 | 2.29E-217 |
| NDUFB2 | 9.4342513 | 7.0889425 | -2.345309 | 4.25E-275 | 8.39E-274 |
| BMP2 | 2.1028858 | 5.691252 | 3.5883661 | 2.23E-272 | 3.51E-271 |
| CDCA7 | 1.6886912 | 4.1583216 | 2.4696303 | 8.32E-265 | 8.56E-264 |
| EGR4 | 3.646068 | 1.3454016 | -2.300666 | 8.55E-153 | 2.13E-152 |
| NEFM | 7.9012989 | 4.4252814 | -3.476018 | 1.49E-168 | 4.14E-168 |
| FTCD | 4.9448698 | 1.2987635 | -3.646106 | 6.64E-273 | 1.09E-271 |
| GLIS3 | 3.2045369 | 5.4269877 | 2.2224508 | 2.24E-213 | 9.03E-213 |
| TMEM179 | 6.8825743 | 2.2179523 | -4.664622 | 1.47E-283 | 2.28E-281 |
| RPL34 | 11.495925 | 8.4911701 | -3.004754 | 7.09E-275 | 1.38E-273 |
| TBC1D5 | 5.5081232 | 7.5268937 | 2.0187705 | 1.24E-278 | 4.00E-277 |
| GCHFR | 5.1162323 | 2.660056 | -2.456176 | 1.09E-269 | 1.43E-268 |
| CKAP2L | 0.4646658 | 2.7803711 | 2.3157053 | 4.37E-252 | 3.03E-251 |
| MRC1 | 1.4780759 | 3.521156 | 2.0430802 | 1.51E-197 | 5.22E-197 |
| CFTR | 2.9488547 | 0.7636229 | -2.185232 | 1.80E-247 | 1.14E-246 |
| HRAS | 8.4159262 | 5.8616982 | -2.554228 | 1.85E-275 | 3.85E-274 |
| CCDC80 | 4.6081988 | 7.518959 | 2.9107602 | 1.56E-238 | 8.51E-238 |
| NF1 | 5.3749082 | 7.6278295 | 2.2529213 | 6.97E-265 | 7.18E-264 |
| SERPINA3 | 4.4433445 | 8.2919746 | 3.8486301 | 6.81E-133 | 1.52E-132 |
| SRGAP1 | 3.3169896 | 5.994896 | 2.6779063 | 2.77E-281 | 1.59E-279 |
| SPARC | 9.7873001 | 12.710167 | 2.922867 | 2.88E-252 | 2.01E-251 |
| PIGL | 5.5316348 | 2.7769931 | -2.754642 | 1.47E-282 | 1.31E-280 |
| MYO15A | 3.6408178 | 1.4598237 | -2.180994 | 4.53E-218 | 1.92E-217 |
| IFT20 | 7.1810861 | 4.5806862 | -2.6004 | 7.53E-274 | 1.32E-272 |
| CCDC154 | 4.0511904 | 1.9015585 | -2.149632 | 1.26E-244 | 7.52E-244 |
| GTF2IRD2P1 | 0.9593702 | 4.7481422 | 3.788772 | 7.85E-284 | 1.42E-281 |
| 4-Sep | 10.361201 | 7.4155177 | -2.945683 | 2.04E-233 | 1.04E-232 |
| MUSTN1 | 5.678417 | 3.1659628 | -2.512454 | 5.40E-244 | 3.20E-243 |
| IGFBP5 | 5.8555402 | 9.2568477 | 3.4013075 | 1.95E-265 | 2.07E-264 |
| NDUFA7 | 7.8333849 | 5.7895406 | -2.043844 | 2.51E-263 | 2.41E-262 |
| RNF208 | 7.4790869 | 4.952909 | -2.526178 | 2.09E-258 | 1.69E-257 |
| AGPS | 4.0468901 | 6.1478284 | 2.1009384 | 6.97E-281 | 3.56E-279 |
| GFOD1 | 5.2488513 | 2.9064889 | -2.342362 | 6.10E-260 | 5.15E-259 |
| MYO10 | 5.8605972 | 8.7234612 | 2.8628639 | 8.95E-264 | 8.79E-263 |
| ASPDH | 6.9155975 | 2.6048583 | -4.310739 | 3.26E-284 | 8.13E-282 |
| SNCA | 8.2364312 | 5.0986991 | -3.137732 | 6.43E-244 | 3.80E-243 |
| ZNF275 | 4.6139165 | 6.9745459 | 2.3606293 | 1.37E-280 | 6.62E-279 |
| ABHD12B | 3.9616813 | 1.4110521 | -2.550629 | 4.98E-226 | 2.32E-225 |
| AUH | 6.0102092 | 4.0012766 | -2.008933 | 5.68E-284 | 1.14E-281 |
| PACSIN1 | 8.0604365 | 4.9474359 | -3.113001 | 3.34E-144 | 7.92E-144 |
| HAGHL | 7.0257662 | 3.9971701 | -3.028596 | 3.17E-277 | 8.44E-276 |
| KCNA1 | 5.9725435 | 3.1382665 | -2.834277 | 7.82E-181 | 2.37E-180 |
| ETV6 | 3.115732 | 5.2664669 | 2.150735 | 4.26E-265 | 4.44E-264 |
| PDIA2 | 7.4931977 | 3.2530324 | -4.240165 | 3.70E-276 | 8.47E-275 |
| TAC3 | 4.369219 | 1.8779458 | -2.491273 | 1.41E-117 | 2.88E-117 |
| C9orf24 | 6.3572388 | 2.1642202 | -4.193019 | 8.29E-264 | 8.16E-263 |
| MDP1 | 6.225032 | 4.1818584 | -2.043174 | 1.24E-275 | 2.62E-274 |
| FAM153A | 5.3560688 | 1.073336 | -4.282733 | 3.57E-264 | 3.56E-263 |
| ZMIZ1 | 6.1750104 | 8.6807896 | 2.5057792 | 2.71E-272 | 4.24E-271 |
| SH3GL3 | 7.0433865 | 4.0575084 | -2.985878 | 3.79E-262 | 3.49E-261 |
| HSP90AB2P | 0.1709877 | 2.1800308 | 2.0090431 | 1.17E-270 | 1.64E-269 |
| MYO16 | 2.2690781 | 4.5237072 | 2.2546291 | 7.49E-220 | 3.25E-219 |
| ANGPTL2 | 4.7313346 | 7.9438354 | 3.2125008 | 1.84E-257 | 1.45E-256 |
| TANC1 | 4.4922242 | 6.5677423 | 2.0755181 | 1.69E-270 | 2.33E-269 |
| SDHAP1 | 5.950491 | 3.6090134 | -2.341478 | 5.05E-281 | 2.69E-279 |
| C3AR1 | 3.002728 | 5.2873527 | 2.2846247 | 4.78E-179 | 1.43E-178 |
| SLC26A8 | 3.3024444 | 1.1215056 | -2.180939 | 7.66E-216 | 3.16E-215 |
| PIPSL | 0.758178 | 3.9331395 | 3.1749615 | 4.53E-280 | 1.91E-278 |
| POFUT1 | 4.2742052 | 6.9281562 | 2.653951 | 1.65E-285 | 3.32E-282 |
| KIFC1 | 0.9172837 | 3.9231544 | 3.0058707 | 1.63E-267 | 1.91E-266 |
| TCEAL7 | 7.7084924 | 5.5684666 | -2.140026 | 1.50E-266 | 1.68E-265 |
| SLC6A7 | 4.2150131 | 2.1291612 | -2.085852 | 1.70E-59 | 2.57E-59 |
| ZNF107 | 2.4788537 | 4.692499 | 2.2136453 | 5.47E-281 | 2.89E-279 |
| SKA3 | 0.9198357 | 2.9369114 | 2.0170757 | 1.51E-248 | 9.75E-248 |
| COL1A2 | 4.3186846 | 6.4098181 | 2.0911334 | 3.44E-138 | 7.90E-138 |
| CARTPT | 4.2269236 | 0.8291989 | -3.397725 | 2.84E-135 | 6.41E-135 |
| CYFIP1 | 5.7916422 | 7.9491438 | 2.1575016 | 2.82E-266 | 3.11E-265 |
| EDARADD | 0.341935 | 2.7818373 | 2.4399024 | 1.10E-278 | 3.57E-277 |
| ST20 | 4.7210533 | 2.6372409 | -2.083812 | 4.52E-273 | 7.50E-272 |
| MYH9 | 6.6613214 | 8.8561603 | 2.1948389 | 1.98E-271 | 2.91E-270 |
| GNA13 | 5.2888772 | 7.6004278 | 2.3115505 | 5.58E-281 | 2.93E-279 |
| C16orf89 | 6.0813855 | 3.8108436 | -2.270542 | 2.83E-226 | 1.32E-225 |
| SLC16A1 | 5.487608 | 7.5162073 | 2.0285993 | 6.00E-219 | 2.57E-218 |
| CHD7 | 5.0258375 | 7.0286561 | 2.0028186 | 2.19E-157 | 5.61E-157 |
| NLGN1 | 3.7988702 | 5.8473406 | 2.0484704 | 8.63E-267 | 9.76E-266 |
| LIME1 | 6.1600815 | 3.0473043 | -3.112777 | 2.16E-276 | 5.10E-275 |
| MRPL52 | 7.8147862 | 5.5753845 | -2.239402 | 5.73E-268 | 6.91E-267 |
| MCTP1 | 5.0692427 | 2.9319657 | -2.137277 | 4.11E-211 | 1.62E-210 |
| CEP55 | 0.3317034 | 2.6293914 | 2.297688 | 2.79E-260 | 2.39E-259 |
| TTC30A | 2.3879688 | 4.6657958 | 2.2778271 | 2.19E-282 | 1.81E-280 |
| CMC1 | 6.1082795 | 3.4549632 | -2.653316 | 4.08E-279 | 1.43E-277 |
| ART3 | 4.3328072 | 2.2404187 | -2.092389 | 2.23E-204 | 8.20E-204 |
| KRTDAP | 2.4323858 | 0.0426558 | -2.38973 | 1.58E-261 | 1.42E-260 |
| NPM2 | 6.7283275 | 2.7022666 | -4.026061 | 4.27E-268 | 5.19E-267 |
| GAB1 | 5.0190775 | 7.0516048 | 2.0325273 | 4.36E-238 | 2.37E-237 |
| PRODH | 7.2272567 | 5.1000188 | -2.127238 | 1.32E-177 | 3.90E-177 |
| TNRC6B | 3.8134755 | 6.2372458 | 2.4237703 | 1.19E-270 | 1.66E-269 |
| RESP18 | 3.6925413 | 0.4231968 | -3.269345 | 1.38E-243 | 8.11E-243 |
| RPS10 | 10.538826 | 8.3438196 | -2.195007 | 5.11E-270 | 6.80E-269 |
| SNCG | 8.9491373 | 4.2723096 | -4.676828 | 2.15E-271 | 3.16E-270 |
| SSBP4 | 8.1852011 | 5.7500455 | -2.435156 | 1.23E-264 | 1.26E-263 |
| ASPHD1 | 8.0958611 | 4.9283356 | -3.167525 | 1.93E-279 | 7.13E-278 |
| TRIM24 | 4.245714 | 6.4874077 | 2.2416937 | 4.89E-285 | 3.32E-282 |
| FBXO16 | 4.5425432 | 2.4372154 | -2.105328 | 1.05E-250 | 7.10E-250 |
| MT1M | 7.1649778 | 3.9191198 | -3.245858 | 6.17E-235 | 3.20E-234 |
| AQP7 | 2.6727994 | 0.6022908 | -2.070509 | 6.32E-239 | 3.47E-238 |
| BUB1 | 0.7547517 | 3.5027678 | 2.7480162 | 1.31E-264 | 1.34E-263 |
| CECR7 | 4.16774 | 2.079614 | -2.088126 | 1.10E-222 | 4.95E-222 |
| PDZD7 | 4.9337204 | 2.3802779 | -2.553442 | 2.18E-257 | 1.71E-256 |
| C12orf57 | 9.057013 | 6.8770628 | -2.17995 | 1.69E-257 | 1.33E-256 |
| NBPF10 | 2.7952901 | 5.3607778 | 2.5654877 | 7.42E-261 | 6.50E-260 |
| LAMC1 | 4.2534782 | 6.6059343 | 2.3524561 | 9.95E-268 | 1.18E-266 |
| SEC24A | 3.3509942 | 5.4657894 | 2.1147952 | 3.42E-282 | 2.58E-280 |
| CRYM | 7.1695506 | 3.542181 | -3.62737 | 9.04E-178 | 2.68E-177 |
| EPB41L4A | 3.3641216 | 5.411142 | 2.0470204 | 3.37E-272 | 5.20E-271 |
| RUNX1 | 2.2077164 | 4.59083 | 2.3831136 | 1.51E-213 | 6.08E-213 |
| DIO3OS | 4.1977381 | 0.3591382 | -3.8386 | 1.27E-281 | 8.04E-280 |
| RTBDN | 3.7485266 | 1.6507988 | -2.097728 | 1.47E-151 | 3.63E-151 |
| TRAPPC5 | 7.5818336 | 5.3082971 | -2.273537 | 6.10E-266 | 6.61E-265 |
| KIAA0408 | 5.8681403 | 0.8300106 | -5.03813 | 1.31E-281 | 8.21E-280 |
| GLUD2 | 1.9340283 | 6.2412775 | 4.3072492 | 3.57E-283 | 4.30E-281 |
| RELL2 | 6.0128038 | 3.6574604 | -2.355343 | 1.31E-237 | 7.09E-237 |
| LAPTM5 | 5.6299765 | 8.4182535 | 2.7882769 | 4.75E-191 | 1.56E-190 |
| AHR | 2.6927712 | 4.817887 | 2.1251158 | 1.11E-245 | 6.79E-245 |
| GPC6 | 1.7527337 | 4.3027978 | 2.5500641 | 9.96E-276 | 2.14E-274 |
| PCDH18 | 2.5553766 | 4.7217393 | 2.1663627 | 1.36E-262 | 1.27E-261 |
| FAM98C | 6.3910757 | 4.3555638 | -2.035512 | 7.34E-278 | 2.15E-276 |
| LIN7B | 6.8037818 | 3.4331772 | -3.370605 | 2.86E-283 | 3.57E-281 |
| VSIG4 | 3.622072 | 6.4006837 | 2.7786117 | 1.21E-173 | 3.48E-173 |
| LUZP6 | 5.5969755 | 9.0538276 | 3.4568521 | 8.59E-280 | 3.43E-278 |
| LTBP1 | 3.5416227 | 5.7023744 | 2.1607517 | 5.85E-218 | 2.48E-217 |
| CEND1 | 8.6588807 | 6.3881442 | -2.270736 | 4.03E-219 | 1.73E-218 |
| ATP9A | 7.0073752 | 9.0166421 | 2.0092668 | 1.37E-221 | 6.05E-221 |
| TRAM2 | 2.8489097 | 4.8764442 | 2.0275346 | 6.60E-273 | 1.08E-271 |
| C9orf3 | 6.3441287 | 4.3224589 | -2.02167 | 1.86E-263 | 1.80E-262 |
| SYCE1 | 5.1680957 | 1.9488624 | -3.219233 | 9.94E-224 | 4.51E-223 |
| TSGA10 | 4.4741573 | 2.3835868 | -2.090571 | 9.03E-279 | 2.98E-277 |
| PDGFRA | 4.864989 | 8.4316631 | 3.5666741 | 6.35E-226 | 2.95E-225 |
| TMEM134 | 7.0869058 | 5.0226134 | -2.064292 | 6.19E-267 | 7.08E-266 |
| CBLN3 | 4.4750657 | 2.1206582 | -2.354407 | 1.97E-194 | 6.63E-194 |
| RPL7 | 10.815266 | 5.8901108 | -4.925155 | 1.12E-275 | 2.38E-274 |
| S100A8 | 5.6357012 | 2.7988646 | -2.836837 | 2.64E-178 | 7.84E-178 |
| CLDND2 | 3.9415361 | 1.2245342 | -2.717002 | 1.71E-277 | 4.70E-276 |
| GFPT1 | 4.1300025 | 6.304963 | 2.1749605 | 3.23E-282 | 2.49E-280 |
| SNORD116-4 | 0.0370024 | 3.8790532 | 3.8420508 | 2.25E-278 | 7.01E-277 |
| CLDN10 | 6.4372033 | 3.7773801 | -2.659823 | 1.72E-205 | 6.39E-205 |
| ASS1 | 6.8217947 | 4.4057047 | -2.41609 | 8.40E-226 | 3.90E-225 |
| TMEM106B | 4.5894376 | 7.4451562 | 2.8557185 | 3.81E-283 | 4.48E-281 |
| GLRX2 | 5.3675273 | 3.23168 | -2.135847 | 1.02E-279 | 4.06E-278 |
| ESRP2 | 1.0836369 | 3.2347728 | 2.1511359 | 2.79E-261 | 2.49E-260 |
| RGS14 | 6.1395833 | 3.1222987 | -3.017285 | 2.30E-152 | 5.72E-152 |
| RPL26 | 11.376295 | 8.9771099 | -2.399185 | 1.25E-274 | 2.37E-273 |
| F8A1 | 6.0459332 | 3.625455 | -2.420478 | 1.10E-280 | 5.48E-279 |
| TARBP1 | 7.0652255 | 5.024505 | -2.04072 | 6.68E-252 | 4.60E-251 |
| MEG3 | 10.781579 | 5.5952372 | -5.186341 | 1.08E-272 | 1.74E-271 |
| NID1 | 3.4544151 | 6.4802128 | 3.0257977 | 2.25E-272 | 3.54E-271 |
| NEURL1B | 3.6267716 | 5.7465752 | 2.1198036 | 1.18E-208 | 4.54E-208 |
| NUDT14 | 6.3966145 | 4.1392179 | -2.257397 | 1.68E-223 | 7.59E-223 |
| CDKN2B | 2.3880675 | 4.5262028 | 2.1381353 | 5.27E-184 | 1.63E-183 |
| RPS3A | 10.99373 | 8.1730826 | -2.820647 | 2.38E-273 | 4.03E-272 |
| TUBA4A | 7.9565764 | 5.4899713 | -2.466605 | 4.79E-193 | 1.60E-192 |
| PIK3R1 | 6.2379612 | 8.8201101 | 2.5821489 | 9.29E-261 | 8.10E-260 |
| FAM181B | 5.2426603 | 7.4013399 | 2.1586796 | 7.81E-231 | 3.84E-230 |
| NNAT | 8.1261133 | 4.6054143 | -3.520699 | 5.73E-180 | 1.72E-179 |
| HSD11B1L | 7.2555925 | 5.2473287 | -2.008264 | 7.14E-253 | 5.05E-252 |
| TLR4 | 3.987692 | 6.0107477 | 2.0230557 | 9.44E-208 | 3.59E-207 |
| SNORD116-28 | 0.034023 | 2.8172887 | 2.7832657 | 1.58E-279 | 5.98E-278 |
| STXBP6 | 5.1791072 | 3.1328288 | -2.046278 | 6.21E-152 | 1.54E-151 |
| KIF20A | 0.426235 | 3.0382056 | 2.6119706 | 1.16E-249 | 7.62E-249 |
| MFRP | 0.1264883 | 6.5102852 | 6.3837969 | 1.75E-286 | 1.26E-282 |
| VCAN | 5.1396553 | 9.3429261 | 4.2032707 | 1.40E-280 | 6.75E-279 |
| TTC32 | 5.611575 | 3.2317598 | -2.379815 | 4.69E-280 | 1.96E-278 |
| FOXK1 | 4.5413472 | 7.0180867 | 2.4767395 | 3.93E-277 | 1.03E-275 |
| FAM153C | 6.0679154 | 0.2746258 | -5.79329 | 8.68E-280 | 3.46E-278 |
| MCM10 | 0.3920979 | 2.7239274 | 2.3318295 | 4.95E-255 | 3.66E-254 |
| HPRT1 | 6.896328 | 4.6461503 | -2.250178 | 8.32E-242 | 4.77E-241 |
| TET2 | 3.0245665 | 5.6911303 | 2.6665638 | 2.78E-284 | 7.29E-282 |
| NDUFB4 | 8.8522847 | 6.6354418 | -2.216843 | 4.08E-283 | 4.65E-281 |
| FAM106A | 4.6880077 | 1.1734124 | -3.514595 | 1.30E-272 | 2.09E-271 |
| A2M | 7.1613868 | 10.271755 | 3.1103686 | 9.53E-274 | 1.65E-272 |
| CA7 | 3.7928798 | 1.4176731 | -2.375207 | 3.62E-158 | 9.31E-158 |
| HSPG2 | 3.6889377 | 5.9069409 | 2.2180032 | 2.09E-198 | 7.30E-198 |
| GHRHR | 2.2173597 | 0.0527193 | -2.16464 | 1.81E-257 | 1.43E-256 |
| HBA2 | 9.494015 | 6.8704525 | -2.623563 | 1.04E-145 | 2.48E-145 |
| MRPL33 | 8.2507864 | 5.1855786 | -3.065208 | 1.71E-276 | 4.05E-275 |
| TNFRSF25 | 6.9414411 | 2.7426042 | -4.198837 | 4.82E-282 | 3.54E-280 |
| OTOS | 3.4566306 | 1.2598831 | -2.196747 | 1.51E-176 | 4.43E-176 |
| NCKAP1L | 2.6765501 | 5.1056902 | 2.4291401 | 2.81E-217 | 1.18E-216 |
| ESPL1 | 0.5624173 | 3.0091018 | 2.4466844 | 1.89E-256 | 1.45E-255 |
| PPP1R3E | 7.0093198 | 3.9030941 | -3.106226 | 4.43E-283 | 4.93E-281 |
| SULT4A1 | 7.3574302 | 4.2906032 | -3.066827 | 1.07E-169 | 3.00E-169 |
| TNKS | 5.2346873 | 7.5618383 | 2.327151 | 2.40E-281 | 1.39E-279 |
| DCLK2 | 6.5445596 | 8.8833102 | 2.3387506 | 1.07E-261 | 9.70E-261 |
| FRMPD2 | 3.7717986 | 1.5594377 | -2.212361 | 1.08E-183 | 3.34E-183 |
| S1PR3 | 3.5965258 | 5.8103469 | 2.2138211 | 1.41E-191 | 4.65E-191 |
| CSAD | 6.8757844 | 4.6631056 | -2.212679 | 1.31E-268 | 1.63E-267 |
| STX8 | 7.1074629 | 5.0585352 | -2.048928 | 1.53E-267 | 1.80E-266 |
| TRIM3 | 6.7989366 | 4.7833332 | -2.015603 | 7.67E-270 | 1.01E-268 |
| TAF1D | 8.0641545 | 5.1791393 | -2.885015 | 1.52E-276 | 3.66E-275 |
| ARHGEF33 | 3.9076932 | 1.8319856 | -2.075708 | 5.24E-264 | 5.20E-263 |
| SLC4A4 | 5.6161641 | 8.5671783 | 2.9510142 | 6.46E-225 | 2.97E-224 |
| NME3 | 8.0142828 | 5.5101255 | -2.504157 | 1.46E-270 | 2.02E-269 |
| WLS | 6.5286326 | 8.7348905 | 2.2062579 | 3.23E-219 | 1.39E-218 |
| SIGLEC10 | 2.904096 | 5.1127721 | 2.2086761 | 3.47E-192 | 1.15E-191 |
| RPL17 | 11.026831 | 7.6611767 | -3.365654 | 5.25E-276 | 1.18E-274 |
| SNCB | 9.0337935 | 5.2895968 | -3.744197 | 1.58E-206 | 5.92E-206 |
| SLC7A10 | 4.876487 | 1.8991713 | -2.977316 | 4.90E-204 | 1.80E-203 |
| MAL2 | 5.5204597 | 2.8627933 | -2.657666 | 4.48E-155 | 1.13E-154 |
| APC | 5.9038321 | 8.0801217 | 2.1762896 | 6.41E-253 | 4.53E-252 |
| GAD2 | 5.4493325 | 2.4919287 | -2.957404 | 2.72E-177 | 8.01E-177 |
| ZNF805 | 1.4377106 | 4.3843999 | 2.9466894 | 3.38E-277 | 8.97E-276 |
| ZNF292 | 4.1753508 | 6.2672863 | 2.0919356 | 8.44E-263 | 7.96E-262 |
| C15orf39 | 3.2257963 | 5.3208579 | 2.0950616 | 8.39E-275 | 1.61E-273 |
| FKBP1B | 6.9335338 | 3.3801974 | -3.553336 | 2.54E-275 | 5.19E-274 |
| STRC | 5.0315708 | 2.2477283 | -2.783842 | 5.34E-252 | 3.69E-251 |
| WSCD1 | 5.2988244 | 7.7472712 | 2.4484468 | 2.01E-250 | 1.35E-249 |
| HIST3H2A | 5.7469986 | 3.1306648 | -2.616334 | 2.31E-266 | 2.55E-265 |
| MMP17 | 6.4272597 | 4.1284797 | -2.29878 | 3.12E-157 | 8.01E-157 |
| GH1 | 2.1424503 | 0.0295222 | -2.112928 | 5.51E-124 | 1.17E-123 |
| MT3 | 12.23227 | 9.1091979 | -3.123072 | 2.31E-245 | 1.40E-244 |
| MLXIPL | 5.8105755 | 3.7219664 | -2.088609 | 5.72E-197 | 1.97E-196 |
| IQSEC3 | 7.1926065 | 3.8504401 | -3.342166 | 7.32E-232 | 3.65E-231 |
| KIFC2 | 8.2401191 | 5.963863 | -2.276256 | 1.21E-201 | 4.37E-201 |
| MRPL21 | 7.362496 | 5.3252954 | -2.037201 | 1.21E-272 | 1.95E-271 |
| SLC24A2 | 5.122477 | 2.8503577 | -2.272119 | 1.01E-202 | 3.66E-202 |
| IGFL4 | 3.6206865 | 0.6423122 | -2.978374 | 3.83E-281 | 2.12E-279 |
| PTGDS | 11.957038 | 9.0235941 | -2.933444 | 8.03E-237 | 4.28E-236 |
| KIF18B | 0.7681186 | 3.8700367 | 3.1019181 | 1.73E-275 | 3.62E-274 |
| APLNR | 5.1105998 | 7.3293916 | 2.2187918 | 5.55E-105 | 1.06E-104 |
| KIF14 | 0.2530291 | 2.7671846 | 2.5141555 | 3.31E-278 | 1.02E-276 |
| BCYRN1 | 5.1346614 | 0.011001 | -5.12366 | 3.07E-282 | 2.38E-280 |
| LIFR | 4.9294575 | 8.2438681 | 3.3144106 | 9.76E-275 | 1.87E-273 |
| NACC2 | 6.7218616 | 4.4827165 | -2.239145 | 5.68E-271 | 8.16E-270 |
| IL17RD | 3.2905241 | 6.5477519 | 3.2572278 | 7.90E-285 | 3.49E-282 |
| ETS1 | 4.2997056 | 6.5899415 | 2.290236 | 1.69E-246 | 1.05E-245 |
| GNG13 | 3.5179999 | 0.9803944 | -2.537606 | 1.06E-106 | 2.05E-106 |
| ACYP1 | 6.5628525 | 3.2241662 | -3.338686 | 4.97E-285 | 3.32E-282 |
| NCOA3 | 3.740531 | 5.8195085 | 2.0789774 | 1.52E-283 | 2.32E-281 |
| PCDHGC4 | 3.5760154 | 5.5764101 | 2.0003947 | 5.44E-137 | 1.24E-136 |
| AMOTL1 | 4.0351782 | 6.7270963 | 2.6919181 | 2.87E-283 | 3.57E-281 |
| PCDHB16 | 2.1005254 | 4.4259877 | 2.3254623 | 7.49E-267 | 8.52E-266 |
| CHST9 | 1.7053368 | 5.4257814 | 3.7204447 | 4.32E-249 | 2.81E-248 |
| PSMG4 | 5.8789381 | 3.5293335 | -2.349605 | 9.53E-282 | 6.26E-280 |
| RUNDC3A | 9.5288454 | 6.8705345 | -2.658311 | 3.40E-236 | 1.80E-235 |
| GNRHR2 | 4.3029978 | 6.4240141 | 2.1210164 | 1.01E-282 | 9.65E-281 |
| PRKCA | 5.879442 | 8.388978 | 2.509536 | 1.13E-268 | 1.41E-267 |
| EIF3CL | 6.5270029 | 9.4425555 | 2.9155526 | 2.67E-283 | 3.44E-281 |
| GLB1L3 | 3.7333913 | 1.2300253 | -2.503366 | 6.48E-247 | 4.04E-246 |
| SNX12 | 6.4023028 | 4.3619669 | -2.040336 | 1.59E-279 | 5.99E-278 |
| HAR1A | 4.3479877 | 2.234393 | -2.113595 | 1.34E-202 | 4.87E-202 |
| WNT10B | 4.4946775 | 2.1409813 | -2.353696 | 3.47E-93 | 6.25E-93 |
| ABTB1 | 7.4308174 | 4.8864474 | -2.54437 | 1.83E-271 | 2.70E-270 |
| PEBP4 | 4.5536607 | 2.5182099 | -2.035451 | 8.12E-202 | 2.92E-201 |
| HELZ | 3.9865792 | 6.4464631 | 2.4598839 | 3.24E-280 | 1.40E-278 |
| GJB6 | 6.0917407 | 2.5870106 | -3.50473 | 7.55E-187 | 2.39E-186 |
| SOX8 | 6.7074875 | 9.5831771 | 2.8756896 | 4.56E-170 | 1.28E-169 |
| BRSK1 | 8.3839 | 6.318326 | -2.065574 | 3.03E-242 | 1.75E-241 |
| MIA2 | 5.1065297 | 0.0889645 | -5.017565 | 3.50E-283 | 4.25E-281 |
| KIF12 | 2.9831102 | 0.5696101 | -2.4135 | 3.63E-222 | 1.61E-221 |
| KRT19 | 3.1518439 | 1.1405831 | -2.011261 | 3.75E-181 | 1.14E-180 |
| BHLHE22 | 3.6558887 | 1.6443562 | -2.011533 | 3.67E-159 | 9.54E-159 |
| HIPK2 | 7.0990063 | 9.7322444 | 2.6332381 | 1.80E-202 | 6.50E-202 |
| URB2 | 2.7086505 | 4.7352003 | 2.0265498 | 1.62E-283 | 2.40E-281 |
| VSX1 | 3.6326872 | 1.1596934 | -2.472994 | 7.07E-260 | 5.95E-259 |
| NCAM1 | 8.2925182 | 10.461451 | 2.1689326 | 6.83E-229 | 3.28E-228 |
| EDNRA | 2.3798733 | 4.8792353 | 2.499362 | 4.45E-257 | 3.46E-256 |
| MCM2 | 3.3682559 | 5.5496635 | 2.1814076 | 2.07E-246 | 1.28E-245 |
| APOL6 | 2.5436979 | 5.5258893 | 2.9821914 | 7.51E-273 | 1.22E-271 |
| NGB | 4.4756682 | 1.7418581 | -2.73381 | 2.92E-186 | 9.20E-186 |
| TXN | 8.2773258 | 6.1515766 | -2.125749 | 3.35E-265 | 3.50E-264 |
| SMN1 | 6.080332 | 1.1981476 | -4.882184 | 3.29E-282 | 2.52E-280 |
| PSMC1 | 7.6264444 | 4.996789 | -2.629655 | 7.98E-279 | 2.66E-277 |
| PSPN | 2.7629555 | 0.5814176 | -2.181538 | 2.96E-279 | 1.06E-277 |
| ARHGAP31 | 3.701249 | 7.3591909 | 3.6579419 | 1.90E-285 | 3.32E-282 |
| SPATA1 | 2.7278677 | 0.521459 | -2.206409 | 1.32E-267 | 1.56E-266 |
| CHKB | 7.3825922 | 3.8696924 | -3.5129 | 3.20E-281 | 1.80E-279 |
| PICK1 | 6.6763164 | 4.3571671 | -2.319149 | 1.46E-276 | 3.53E-275 |
| TRIM17 | 5.0212492 | 2.3116356 | -2.709614 | 1.31E-204 | 4.84E-204 |
| RGPD3 | 1.0979761 | 4.0575702 | 2.9595941 | 1.46E-277 | 4.07E-276 |
| SLC1A2 | 8.1258124 | 10.233624 | 2.1078119 | 1.30E-103 | 2.46E-103 |
| IQGAP2 | 2.0168816 | 4.1952058 | 2.1783242 | 1.78E-201 | 6.38E-201 |
| SFT2D1 | 7.2143515 | 5.0879624 | -2.126389 | 8.05E-275 | 1.55E-273 |
| SNORA70 | 2.618298 | 0.0680118 | -2.550286 | 2.12E-25 | 2.64E-25 |
| CBWD6 | 5.8150046 | 3.6351513 | -2.179853 | 1.20E-270 | 1.68E-269 |
| WASF2 | 5.7334556 | 7.8714752 | 2.1380196 | 9.06E-267 | 1.02E-265 |
| SLC16A8 | 3.6875067 | 1.5038153 | -2.183691 | 2.05E-255 | 1.53E-254 |
| TTC28 | 3.3072752 | 5.9442206 | 2.6369455 | 4.05E-284 | 9.59E-282 |
| COX8A | 9.9315093 | 7.6476235 | -2.283886 | 1.24E-271 | 1.84E-270 |
| LRRC7 | 5.0693667 | 2.734309 | -2.335058 | 1.07E-155 | 2.72E-155 |
| POLH | 2.9052149 | 5.1393131 | 2.2340981 | 9.45E-284 | 1.63E-281 |
| CYP46A1 | 7.1251331 | 5.1069739 | -2.018159 | 3.67E-144 | 8.69E-144 |
| PTPN13 | 4.4362614 | 6.5569839 | 2.1207225 | 1.11E-254 | 8.18E-254 |
| C5orf49 | 4.4469687 | 2.3678136 | -2.079155 | 1.09E-146 | 2.63E-146 |
| MARCKS | 7.1880809 | 10.475086 | 3.2870053 | 1.11E-283 | 1.86E-281 |
| CPLX2 | 9.0086581 | 6.7313707 | -2.277287 | 1.46E-87 | 2.56E-87 |
| BCAN | 8.0704849 | 12.196364 | 4.1258792 | 8.75E-247 | 5.44E-246 |
| SRCAP | 4.9168416 | 7.9174524 | 3.0006108 | 2.52E-283 | 3.31E-281 |
| RGMA | 6.6285497 | 9.050191 | 2.4216413 | 1.30E-235 | 6.79E-235 |
| DCX | 2.2353756 | 6.1844159 | 3.9490403 | 6.57E-235 | 3.41E-234 |
| SFTPC | 5.043429 | 2.0310471 | -3.012382 | 4.91E-271 | 7.07E-270 |
| C6orf89 | 5.9855511 | 8.1779167 | 2.1923656 | 3.43E-284 | 8.40E-282 |
| MYL3 | 4.5419579 | 2.1040218 | -2.437936 | 2.20E-264 | 2.21E-263 |
| LY6G5B | 5.2296844 | 3.2226912 | -2.006993 | 1.09E-249 | 7.16E-249 |
| BIRC5 | 1.2308284 | 3.5064623 | 2.2756339 | 2.69E-191 | 8.84E-191 |
| ALKBH6 | 6.2203741 | 3.5783203 | -2.642054 | 7.52E-285 | 3.49E-282 |
| PANX2 | 6.0289687 | 3.7568927 | -2.272076 | 4.22E-218 | 1.79E-217 |
| CXCR5 | 2.7234964 | 0.5105375 | -2.212959 | 6.15E-228 | 2.92E-227 |
| SDHAP2 | 0.5733019 | 3.6560767 | 3.0827748 | 2.43E-277 | 6.59E-276 |
| HNF1A | 0.1855529 | 2.3886303 | 2.2030774 | 1.50E-276 | 3.60E-275 |
| MRPL41 | 8.3045227 | 5.3630201 | -2.941503 | 6.64E-271 | 9.44E-270 |
| SLC6A12 | 5.8471797 | 3.7393399 | -2.10784 | 3.68E-244 | 2.19E-243 |
| WNT5A | 2.3316767 | 5.0979756 | 2.7662989 | 4.36E-277 | 1.13E-275 |
| VANGL2 | 4.4163637 | 7.5494414 | 3.1330777 | 7.75E-276 | 1.69E-274 |
| ZNF24 | 5.7005821 | 7.7581573 | 2.0575753 | 3.99E-282 | 2.97E-280 |
| RFX7 | 3.1841343 | 5.7948156 | 2.6106813 | 2.19E-279 | 7.99E-278 |
| YDJC | 5.5372061 | 3.1990788 | -2.338127 | 4.59E-280 | 1.93E-278 |
| AP3S1 | 7.8449547 | 5.6209403 | -2.224014 | 8.96E-279 | 2.97E-277 |
| MICAL2 | 7.2117515 | 4.8405516 | -2.3712 | 3.49E-144 | 8.26E-144 |
| MT1G | 7.9691941 | 3.4825005 | -4.486694 | 2.21E-248 | 1.42E-247 |
| CDKN2D | 7.5499713 | 4.6416175 | -2.908354 | 2.43E-272 | 3.82E-271 |
| MPEG1 | 1.8053162 | 4.4530139 | 2.6476977 | 1.97E-258 | 1.59E-257 |
| C1QC | 5.7670237 | 7.9859131 | 2.2188894 | 8.79E-131 | 1.94E-130 |
| CX3CR1 | 3.2916949 | 6.3249428 | 3.0332479 | 3.05E-193 | 1.02E-192 |
| SH3RF3 | 3.0673262 | 5.4872992 | 2.4199731 | 2.90E-274 | 5.30E-273 |
| CMKLR1 | 1.9668632 | 4.7333062 | 2.766443 | 1.03E-269 | 1.36E-268 |
| CALML6 | 2.5150665 | 0.401376 | -2.113691 | 5.58E-266 | 6.05E-265 |
| CYP2C8 | 3.282769 | 0.9344618 | -2.348307 | 1.00E-263 | 9.80E-263 |
| CDH2 | 5.6085238 | 7.7803002 | 2.1717764 | 1.16E-275 | 2.45E-274 |
| IPO9 | 5.8395111 | 7.9503519 | 2.1108408 | 3.33E-285 | 3.32E-282 |
| PTPRS | 6.4324726 | 8.5558425 | 2.1233699 | 1.90E-262 | 1.77E-261 |
| SLC2A11 | 6.2370761 | 4.1633345 | -2.073742 | 4.56E-281 | 2.46E-279 |
| POLR2I | 8.6127653 | 5.5293336 | -3.083432 | 1.04E-275 | 2.23E-274 |
| CHD9 | 4.6907044 | 6.8600653 | 2.1693609 | 3.89E-272 | 5.97E-271 |
| H3F3C | 0.3900757 | 4.6399082 | 4.2498324 | 8.57E-283 | 8.45E-281 |
| DNAJC25-GNG10 | 5.1744414 | 0.0696033 | -5.104838 | 9.46E-282 | 6.24E-280 |
| NOTCH1 | 4.5328562 | 8.4500203 | 3.9171641 | 8.28E-283 | 8.26E-281 |
| FADS2 | 7.8232552 | 10.182688 | 2.3594326 | 2.41E-232 | 1.21E-231 |
| RBM47 | 1.3427585 | 3.705978 | 2.3632195 | 1.57E-247 | 9.97E-247 |
| COL1A1 | 3.0484334 | 5.4206903 | 2.3722569 | 1.02E-110 | 2.00E-110 |
| STAG3 | 5.3243125 | 2.9153652 | -2.408947 | 7.45E-275 | 1.44E-273 |
| PFDN5 | 10.549579 | 8.3774505 | -2.172129 | 1.48E-263 | 1.43E-262 |
| ITPRIPL2 | 2.3535859 | 5.3409704 | 2.9873845 | 4.71E-283 | 5.12E-281 |
| CSMD2 | 3.1670683 | 5.1706539 | 2.0035856 | 3.79E-227 | 1.78E-226 |
| ANKRD29 | 4.693732 | 2.4855667 | -2.208165 | 8.60E-253 | 6.05E-252 |
| MEX3A | 1.7385949 | 5.9167597 | 4.1781649 | 3.13E-283 | 3.83E-281 |
| TMSB10 | 11.19846 | 9.0923804 | -2.106079 | 2.60E-173 | 7.45E-173 |
| ZNF609 | 4.7007242 | 6.783002 | 2.0822778 | 1.29E-268 | 1.60E-267 |
| FAM86B1 | 5.2610654 | 3.034437 | -2.226628 | 3.71E-270 | 4.98E-269 |
| CCK | 6.0802957 | 3.6171735 | -2.463122 | 1.95E-60 | 2.96E-60 |
| ASF1B | 0.8632644 | 3.500478 | 2.6372136 | 6.82E-266 | 7.36E-265 |
| RAB31 | 7.0244445 | 9.6930198 | 2.6685753 | 4.04E-270 | 5.41E-269 |
| RFPL2 | 3.838161 | 1.2498864 | -2.588275 | 3.46E-225 | 1.59E-224 |
| RPS27 | 12.244169 | 4.2046823 | -8.039487 | 1.24E-277 | 3.48E-276 |
| S100A1 | 9.6413405 | 5.8373365 | -3.804004 | 4.02E-218 | 1.70E-217 |
| DLL3 | 4.0501125 | 7.4005508 | 3.3504383 | 4.25E-182 | 1.30E-181 |
| HBB | 9.681406 | 7.0604247 | -2.620981 | 3.91E-126 | 8.38E-126 |
| CPNE9 | 4.3371984 | 1.7647183 | -2.57248 | 9.92E-165 | 2.67E-164 |
| CA11 | 9.5463175 | 6.3494369 | -3.196881 | 6.63E-244 | 3.93E-243 |
| SYNGR3 | 7.4911196 | 4.2101369 | -3.280983 | 9.94E-228 | 4.71E-227 |
| PCDHB9 | 2.0331598 | 4.924247 | 2.8910872 | 1.08E-278 | 3.50E-277 |
| XYLT1 | 3.8596344 | 5.9874599 | 2.1278255 | 2.39E-237 | 1.28E-236 |
| CNGA3 | 1.0890859 | 4.0168839 | 2.927798 | 3.81E-225 | 1.75E-224 |
| VSNL1 | 8.5386149 | 5.1277124 | -3.410903 | 1.11E-140 | 2.59E-140 |
| IKZF1 | 1.7128696 | 4.1457944 | 2.4329248 | 3.15E-245 | 1.90E-244 |
| C2orf74 | 7.322581 | 4.675905 | -2.646676 | 6.69E-278 | 1.97E-276 |
| TEAD1 | 4.3068494 | 7.3808149 | 3.0739655 | 2.00E-285 | 3.32E-282 |
| SRPX | 4.0813063 | 6.1452425 | 2.0639362 | 1.13E-167 | 3.10E-167 |
| RIMS2 | 6.0502226 | 3.6027052 | -2.447517 | 1.06E-173 | 3.04E-173 |
| ANKRD36B | 4.2997777 | 2.2139014 | -2.085876 | 2.84E-259 | 2.34E-258 |
| ADAM6 | 0.0393742 | 2.8657891 | 2.8264149 | 1.44E-280 | 6.91E-279 |
| GNG8 | 3.0660957 | 0.4188639 | -2.647232 | 2.60E-134 | 5.83E-134 |
| CDCP1 | 0.9201124 | 3.0779288 | 2.1578164 | 2.48E-254 | 1.81E-253 |
| ARHGAP30 | 2.4950602 | 4.6851776 | 2.1901174 | 9.76E-240 | 5.41E-239 |
| HAPLN1 | 1.8589925 | 4.7221392 | 2.8631468 | 6.46E-214 | 2.62E-213 |
| COL7A1 | 6.1660184 | 4.0384632 | -2.127555 | 1.06E-202 | 3.84E-202 |
| IFI27L2 | 8.3164568 | 6.0344771 | -2.28198 | 2.01E-259 | 1.67E-258 |
| RPS26 | 9.1878268 | 5.2156524 | -3.972174 | 2.92E-276 | 6.80E-275 |
| PIN1 | 8.7855871 | 6.5981602 | -2.187427 | 1.55E-273 | 2.64E-272 |
| SULT1A1 | 6.6141665 | 3.9505804 | -2.663586 | 2.45E-271 | 3.58E-270 |
| LCNL1 | 5.7065222 | 2.6262542 | -3.080268 | 1.31E-253 | 9.41E-253 |
| CPNE3 | 5.3501494 | 7.5933178 | 2.2431684 | 1.52E-282 | 1.34E-280 |
| CLPS | 2.1198704 | 0.0747417 | -2.045129 | 4.29E-127 | 9.24E-127 |
| NDUFS5 | 10.080593 | 7.2718695 | -2.808724 | 4.20E-277 | 1.09E-275 |
| NES | 5.3014783 | 10.04583 | 4.7443515 | 2.40E-281 | 1.39E-279 |
| BNC2 | 0.8396109 | 2.8915223 | 2.0519114 | 6.37E-261 | 5.60E-260 |
| HBQ1 | 3.6194004 | 1.095073 | -2.524327 | 9.87E-224 | 4.48E-223 |
| BACH1 | 3.5777936 | 5.6145434 | 2.0367498 | 2.16E-275 | 4.47E-274 |
| CCDC85B | 7.8744829 | 5.3253446 | -2.549138 | 1.90E-250 | 1.28E-249 |
| C4orf48 | 7.2484281 | 3.7375845 | -3.510844 | 4.38E-269 | 5.58E-268 |
| SEMA5A | 3.5221309 | 7.5618862 | 4.0397553 | 1.88E-281 | 1.12E-279 |
| SELL | 2.8719733 | 5.4904587 | 2.6184853 | 1.03E-163 | 2.75E-163 |
| HIGD1B | 6.1574756 | 3.1104839 | -3.046992 | 4.84E-275 | 9.51E-274 |
| TUBB1 | 0.5748014 | 3.1666259 | 2.5918245 | 8.34E-278 | 2.42E-276 |
| ADAMTS6 | 1.1251986 | 3.7853095 | 2.6601109 | 5.56E-275 | 1.09E-273 |
| COX5A | 9.0537136 | 6.5238451 | -2.529869 | 3.20E-278 | 9.87E-277 |
| LAT | 5.0786798 | 2.920044 | -2.158636 | 1.40E-261 | 1.26E-260 |
| CCDC50 | 4.8567329 | 7.381378 | 2.5246451 | 5.17E-285 | 3.32E-282 |
| RBM11 | 4.1035191 | 1.9651904 | -2.138329 | 3.56E-246 | 2.18E-245 |
| TNS1 | 5.7383108 | 7.910325 | 2.1720142 | 2.25E-245 | 1.36E-244 |
| COMMD1 | 6.3878324 | 4.274552 | -2.11328 | 3.31E-270 | 4.48E-269 |
| MT1F | 7.2772692 | 4.6592281 | -2.618041 | 4.68E-237 | 2.50E-236 |
| C2orf40 | 4.8680642 | 2.6567449 | -2.211319 | 3.04E-225 | 1.40E-224 |
| PWRN1 | 2.5457486 | 0.1979459 | -2.347803 | 9.02E-222 | 4.00E-221 |
| WASH5P | 6.3045878 | 4.1945714 | -2.110016 | 6.01E-262 | 5.49E-261 |
| CATSPER2 | 5.2574003 | 2.3804196 | -2.876981 | 2.19E-283 | 3.08E-281 |
| RIPPLY2 | 5.8469953 | 2.3063352 | -3.54066 | 8.60E-283 | 8.45E-281 |
| CPLX3 | 4.3456688 | 2.1957739 | -2.149895 | 1.08E-133 | 2.43E-133 |
| HEG1 | 4.3635647 | 6.7589429 | 2.3953782 | 1.55E-271 | 2.30E-270 |
| BMF | 2.0904739 | 4.2377844 | 2.1473105 | 6.97E-245 | 4.19E-244 |
| POLB | 6.7595416 | 4.4705323 | -2.289009 | 6.86E-274 | 1.21E-272 |
| HCFC1R1 | 8.8398178 | 5.8899255 | -2.949892 | 2.48E-275 | 5.08E-274 |
| GABRD | 6.8778679 | 4.3538215 | -2.524046 | 5.03E-121 | 1.05E-120 |
| A1BG | 6.1241737 | 3.3770086 | -2.747165 | 5.95E-269 | 7.54E-268 |
| CDK6 | 3.0892104 | 6.1377888 | 3.0485783 | 9.13E-271 | 1.29E-269 |
| ZBTB8OS | 6.3571503 | 3.4654463 | -2.891704 | 2.00E-282 | 1.70E-280 |
| CYP2D6 | 3.8093538 | 1.6448532 | -2.164501 | 2.21E-240 | 1.24E-239 |
| UQCRQ | 9.3597255 | 6.8456102 | -2.514115 | 6.84E-267 | 7.81E-266 |
| SLC39A14 | 4.6367781 | 7.0027438 | 2.3659658 | 5.05E-247 | 3.16E-246 |
| COL4A2 | 4.4096934 | 7.1331172 | 2.7234239 | 6.01E-200 | 2.13E-199 |
| PHLDA1 | 5.0297295 | 8.9438737 | 3.9141442 | 7.71E-280 | 3.11E-278 |
| OPHN1 | 4.4692205 | 6.8537374 | 2.384517 | 7.87E-229 | 3.78E-228 |
| CD86 | 2.2281734 | 4.3201436 | 2.0919702 | 2.35E-182 | 7.18E-182 |
| TNFRSF21 | 5.6957212 | 7.917355 | 2.2216338 | 6.58E-225 | 3.03E-224 |
| RAP2A | 6.0867398 | 8.2970414 | 2.2103016 | 1.68E-255 | 1.25E-254 |
| ANKFY1 | 5.7233352 | 8.2424597 | 2.5191245 | 1.06E-284 | 3.95E-282 |
| RND2 | 6.3222474 | 8.7084191 | 2.3861717 | 4.28E-214 | 1.74E-213 |
| STARD10 | 7.3956655 | 5.2556146 | -2.140051 | 4.84E-270 | 6.45E-269 |
| ADAMTS5 | 1.2126283 | 3.683656 | 2.4710277 | 1.28E-206 | 4.81E-206 |
| PTGS1 | 2.359659 | 4.9970167 | 2.6373577 | 2.20E-227 | 1.04E-226 |
| ANTXR1 | 5.3608256 | 8.4621011 | 3.1012755 | 2.59E-280 | 1.17E-278 |
| PHC3 | 3.6869171 | 6.1748687 | 2.4879517 | 3.12E-279 | 1.12E-277 |
| ALCAM | 6.0606452 | 8.1325654 | 2.0719201 | 4.18E-193 | 1.39E-192 |
| MGST3 | 8.8513907 | 6.7031183 | -2.148272 | 2.85E-273 | 4.79E-272 |
| AMY2B | 6.9901184 | 3.9534474 | -3.036671 | 1.15E-277 | 3.26E-276 |
| IGFBP6 | 5.9226506 | 3.3092829 | -2.613368 | 6.19E-235 | 3.21E-234 |
| TAF10 | 8.1296643 | 5.7260765 | -2.403588 | 1.01E-262 | 9.49E-262 |
| GSTT2 | 1.6043629 | 5.114945 | 3.5105821 | 2.77E-264 | 2.77E-263 |
| TNNC1 | 3.5227459 | 0.5936649 | -2.929081 | 1.42E-279 | 5.42E-278 |
| NTAN1 | 6.5058032 | 4.1940535 | -2.31175 | 3.40E-277 | 8.99E-276 |
| RANBP2 | 5.065732 | 7.2223926 | 2.1566606 | 8.14E-276 | 1.76E-274 |
| FAT4 | 1.5931717 | 4.1137085 | 2.5205367 | 4.46E-271 | 6.44E-270 |
| COX6A2 | 3.2792241 | 0.4795813 | -2.799643 | 6.41E-274 | 1.13E-272 |
| FLVCR2 | 2.0474129 | 4.2374075 | 2.1899946 | 3.44E-243 | 2.01E-242 |
| RASAL1 | 5.1053678 | 2.746345 | -2.359023 | 1.14E-142 | 2.68E-142 |
| LRRC58 | 4.3531336 | 6.8457108 | 2.4925771 | 2.20E-284 | 6.33E-282 |
| TLX1 | 0.0480668 | 2.0621718 | 2.0141051 | 8.50E-225 | 3.90E-224 |
| TP53 | 3.9570168 | 6.4320008 | 2.474984 | 1.91E-261 | 1.71E-260 |
| RMST | 4.1102773 | 1.9209206 | -2.189357 | 5.52E-213 | 2.22E-212 |
| CHAD | 4.7400498 | 1.4757904 | -3.264259 | 2.72E-256 | 2.08E-255 |
| RGMB | 4.9601362 | 7.1682946 | 2.2081584 | 2.46E-249 | 1.61E-248 |
| HAS2 | 0.9361007 | 3.4541728 | 2.518072 | 2.44E-250 | 1.63E-249 |
| NDUFAF2 | 6.6655147 | 3.9370659 | -2.728449 | 1.69E-281 | 1.03E-279 |
| SIGIRR | 6.6498679 | 3.6387815 | -3.011086 | 4.05E-266 | 4.45E-265 |
| SOX9 | 6.1445733 | 8.3233965 | 2.1788232 | 1.66E-220 | 7.25E-220 |
| POU3F2 | 4.2426885 | 6.5259527 | 2.2832643 | 5.88E-268 | 7.09E-267 |
| CPEB1 | 5.7726471 | 3.7482986 | -2.024349 | 1.85E-268 | 2.29E-267 |
| SIX3 | 3.005297 | 0.5810232 | -2.424274 | 7.67E-148 | 1.86E-147 |
| MAP7D2 | 5.5982152 | 3.1971048 | -2.40111 | 1.27E-159 | 3.30E-159 |
| ERLIN2 | 4.9994967 | 7.0760627 | 2.0765661 | 1.23E-278 | 3.97E-277 |
| FAM50B | 5.7991423 | 3.6273851 | -2.171757 | 1.45E-271 | 2.14E-270 |
| WDFY3 | 5.521156 | 7.5662833 | 2.0451273 | 8.97E-272 | 1.34E-270 |
| G0S2 | 4.0379509 | 2.0209038 | -2.017047 | 2.27E-154 | 5.70E-154 |
| HLA-DOA | 2.592767 | 4.81531 | 2.222543 | 5.94E-173 | 1.70E-172 |
| ZNF469 | 1.9352802 | 4.5533022 | 2.618022 | 2.79E-265 | 2.93E-264 |
| TMEM191A | 5.3062947 | 0.9275464 | -4.378748 | 8.31E-284 | 1.48E-281 |
| SETBP1 | 3.1669411 | 5.5249474 | 2.3580063 | 1.75E-260 | 1.51E-259 |
| SIX1 | 0.8468784 | 3.4350171 | 2.5881387 | 1.25E-248 | 8.08E-248 |
| HIF1A | 6.3657811 | 8.5867621 | 2.2209809 | 1.96E-272 | 3.11E-271 |
| NDUFB1 | 9.7139297 | 5.5383267 | -4.175603 | 2.04E-278 | 6.37E-277 |
| DSEL | 3.3851666 | 6.6247394 | 3.2395729 | 4.50E-284 | 1.02E-281 |
| NDST1 | 6.0067003 | 8.3456341 | 2.3389337 | 1.58E-273 | 2.71E-272 |
| ATOX1 | 8.1766174 | 5.8054809 | -2.371137 | 5.43E-270 | 7.21E-269 |
| DPP7 | 9.0744257 | 6.9875507 | -2.086875 | 5.47E-266 | 5.95E-265 |
| TAF1L | 0.0706488 | 2.1131762 | 2.0425274 | 4.99E-278 | 1.50E-276 |
| PFDN6 | 7.8975619 | 5.3642658 | -2.533296 | 6.26E-278 | 1.86E-276 |
| RPS29 | 11.52212 | 8.005618 | -3.516502 | 4.06E-275 | 8.07E-274 |
| NHSL1 | 3.8528362 | 6.0730289 | 2.2201927 | 1.38E-269 | 1.79E-268 |
| MMP16 | 2.9279333 | 5.0794307 | 2.1514974 | 1.77E-234 | 9.12E-234 |
| LAGE3 | 7.0915722 | 5.022158 | -2.069414 | 8.47E-267 | 9.58E-266 |
| URB1 | 3.9590755 | 6.5784245 | 2.6193491 | 2.30E-283 | 3.17E-281 |
| RBP1 | 7.422736 | 5.0009666 | -2.421769 | 1.13E-124 | 2.40E-124 |
| TMEM130 | 7.7162704 | 5.0965326 | -2.619738 | 4.23E-141 | 9.89E-141 |
| AURKB | 0.6332064 | 2.8613965 | 2.2281901 | 1.50E-206 | 5.61E-206 |
| FAM131C | 5.4617825 | 2.8366862 | -2.625096 | 4.00E-236 | 2.11E-235 |
| CCNI2 | 7.5971646 | 2.3868475 | -5.210317 | 7.27E-284 | 1.35E-281 |
| ENTPD1 | 4.5418145 | 6.5475694 | 2.0057549 | 7.40E-285 | 3.49E-282 |
| RGPD1 | 1.8056604 | 3.8479569 | 2.0422965 | 6.12E-207 | 2.30E-206 |
| PPP1R1B | 9.2310884 | 5.8942778 | -3.336811 | 3.87E-208 | 1.48E-207 |
| TAGLN3 | 9.2324337 | 6.0409397 | -3.191494 | 1.54E-240 | 8.64E-240 |
| OLIG2 | 6.0693356 | 8.6538659 | 2.5845304 | 2.19E-187 | 6.99E-187 |
| SSSCA1 | 6.564262 | 4.3404736 | -2.223788 | 1.10E-275 | 2.33E-274 |
| MYO15B | 7.3410555 | 4.8001878 | -2.540868 | 4.44E-258 | 3.54E-257 |
| RPL39 | 10.70068 | 4.2476741 | -6.453006 | 5.92E-278 | 1.77E-276 |
| TMEM100 | 3.9886433 | 6.252686 | 2.2640428 | 8.84E-139 | 2.04E-138 |
| RNF212 | 5.3432331 | 1.0669679 | -4.276265 | 6.35E-281 | 3.28E-279 |
| GPR34 | 3.0137878 | 5.5649236 | 2.5511359 | 1.04E-198 | 3.66E-198 |
| CBWD5 | 7.0976563 | 0.3844199 | -6.713236 | 2.24E-282 | 1.84E-280 |
| IFNAR1 | 5.0493098 | 7.1039568 | 2.054647 | 1.54E-283 | 2.32E-281 |
| FBXO2 | 8.7619241 | 5.7081121 | -3.053812 | 5.33E-259 | 4.37E-258 |
| CLEC2L | 5.6722313 | 2.4718109 | -3.20042 | 3.25E-230 | 1.59E-229 |
| PA2G4P4 | 0.6863814 | 2.9789728 | 2.2925914 | 1.83E-277 | 5.01E-276 |
| HTR5A | 3.851195 | 1.7130153 | -2.13818 | 2.43E-139 | 5.63E-139 |
| ASH1L | 5.3504557 | 7.3964273 | 2.0459717 | 1.19E-262 | 1.12E-261 |
| JAKMIP1 | 6.3324353 | 3.9619487 | -2.370487 | 7.57E-180 | 2.28E-179 |
| GNB4 | 3.8746907 | 7.2428569 | 3.3681662 | 7.97E-285 | 3.49E-282 |
| MRPL55 | 7.4701589 | 5.4014416 | -2.068717 | 7.59E-270 | 1.00E-268 |
| EGFR | 3.9988215 | 7.6942444 | 3.6954229 | 1.87E-245 | 1.13E-244 |
| ZNF264 | 3.2261014 | 5.3738462 | 2.1477448 | 2.09E-278 | 6.51E-277 |
| RPSAP58 | 6.7501247 | 9.3782284 | 2.6281036 | 2.37E-271 | 3.48E-270 |
| ACOX1 | 5.2003899 | 7.2357325 | 2.0353425 | 1.39E-283 | 2.18E-281 |
| MRPL48 | 6.836162 | 4.7710738 | -2.065088 | 1.42E-279 | 5.42E-278 |
| BMPR2 | 5.1090971 | 7.5781422 | 2.4690452 | 9.78E-282 | 6.39E-280 |
| CYB5R2 | 5.1485348 | 2.9616569 | -2.186878 | 2.20E-177 | 6.49E-177 |
| LGI1 | 6.6542795 | 4.1231123 | -2.531167 | 6.26E-230 | 3.04E-229 |
| SAMD9 | 1.343582 | 4.4581863 | 3.1146043 | 2.38E-275 | 4.90E-274 |
| METTL7B | 2.546261 | 4.6862298 | 2.1399688 | 9.36E-110 | 1.83E-109 |
| TNNT3 | 2.1730644 | 0.1615337 | -2.011531 | 9.72E-261 | 8.46E-260 |
| CAMK2B | 8.4575286 | 6.0513231 | -2.406206 | 2.17E-169 | 6.04E-169 |
| MAP3K13 | 6.0190333 | 2.3319459 | -3.687087 | 5.09E-285 | 3.32E-282 |
| SLC35F3 | 4.4856381 | 2.2499874 | -2.235651 | 9.96E-218 | 4.21E-217 |
| CD24 | 4.3047306 | 6.809575 | 2.5048444 | 3.29E-132 | 7.30E-132 |
| GSDMB | 5.5485191 | 2.8823826 | -2.666136 | 3.29E-272 | 5.09E-271 |
| TAC1 | 5.6664087 | 2.5506692 | -3.11574 | 1.35E-126 | 2.91E-126 |
| SULF2 | 6.4710119 | 8.7002271 | 2.2292152 | 2.04E-201 | 7.30E-201 |
| DMRTC1B | 5.6256821 | 3.2142806 | -2.411402 | 1.64E-248 | 1.05E-247 |
| TMEM91 | 6.100213 | 3.4441552 | -2.656058 | 1.53E-262 | 1.43E-261 |
| CD44 | 4.6056366 | 8.4958477 | 3.8902111 | 2.00E-217 | 8.43E-217 |
| SNX32 | 5.9651034 | 3.6044989 | -2.360604 | 2.58E-245 | 1.56E-244 |
| IQCA1 | 5.2070251 | 3.0596458 | -2.147379 | 4.25E-164 | 1.14E-163 |
| BUB1B | 1.2759415 | 3.3313236 | 2.0553821 | 1.90E-196 | 6.52E-196 |
| TBX15 | 1.3167829 | 3.3677691 | 2.0509862 | 9.55E-247 | 5.94E-246 |
| CDHR2 | 2.9076887 | 0.848919 | -2.05877 | 2.41E-217 | 1.01E-216 |
| HYI | 6.7976854 | 4.4817989 | -2.315886 | 2.62E-274 | 4.81E-273 |
| THBS2 | 4.1925792 | 6.7308698 | 2.5382905 | 1.84E-248 | 1.18E-247 |
| ENAH | 5.3997281 | 8.459174 | 3.0594459 | 6.94E-284 | 1.30E-281 |
| ZGLP1 | 5.366513 | 2.9164296 | -2.450083 | 5.95E-268 | 7.15E-267 |
| NRIP1 | 3.5481425 | 6.0483798 | 2.5002373 | 5.43E-280 | 2.23E-278 |
| DNAJA4 | 7.6814455 | 4.9400918 | -2.741354 | 1.95E-261 | 1.75E-260 |
| RPL39L | 4.6730029 | 2.5331741 | -2.139829 | 1.18E-230 | 5.76E-230 |
| PDCD5 | 8.1595541 | 5.5401651 | -2.619389 | 8.29E-277 | 2.07E-275 |
| ABCC4 | 2.1716566 | 4.9152202 | 2.7435636 | 2.79E-280 | 1.24E-278 |
| NLGN4X | 4.364499 | 6.3987369 | 2.0342379 | 5.06E-261 | 4.47E-260 |
| CNFN | 3.3783701 | 1.1315097 | -2.24686 | 5.33E-243 | 3.10E-242 |
| PRSS1 | 2.1710503 | 0.0475158 | -2.123534 | 1.24E-76 | 2.05E-76 |
| OCIAD2 | 6.2592247 | 4.009001 | -2.250224 | 7.55E-150 | 1.85E-149 |
| RPS27A | 10.876597 | 8.7957331 | -2.080864 | 1.22E-269 | 1.60E-268 |
| IPO7 | 6.0749358 | 8.226483 | 2.1515471 | 2.47E-283 | 3.27E-281 |
| OSBPL11 | 4.3716323 | 6.6012262 | 2.2295939 | 3.14E-260 | 2.68E-259 |
| EPHB6 | 7.133048 | 4.6151245 | -2.517923 | 1.61E-149 | 3.94E-149 |
| PCP4L1 | 6.1385117 | 2.864756 | -3.273756 | 2.43E-212 | 9.71E-212 |
| PLEC | 6.4957778 | 9.6551663 | 3.1593885 | 2.16E-273 | 3.66E-272 |
| COX6C | 10.216298 | 7.1644727 | -3.051825 | 6.16E-278 | 1.84E-276 |
| STK17B | 2.9028716 | 5.6912359 | 2.7883642 | 1.39E-278 | 4.43E-277 |
| DEGs: Differentially expressed genes; Control: normal tissue and adjacent tissue; Treatment: tumor tissue (glioma) | | | | | |
